# Integrated mapping of lymphatic filariasis and podoconiosis: lessons learnt from Ethiopia

**DOI:** 10.1186/1756-3305-7-397

**Published:** 2014-08-27

**Authors:** Heven Sime, Kebede Deribe, Ashenafi Assefa, Melanie J Newport, Fikre Enquselassie, Abeba Gebretsadik, Amha Kebede, Asrat Hailu, Oumer Shafi, Abraham Aseffa, Richard Reithinger, Simon J Brooker, Rachel L Pullan, Jorge Cano, Kadu Meribo, Alex Pavluck, Moses J Bockarie, Maria P Rebollo, Gail Davey

**Affiliations:** Ethiopian Public Health Institute, Addis Ababa, Ethiopia; Brighton and Sussex Medical School, Falmer, Brighton, United Kingdom; School of Public Health, Addis Ababa University, Addis Ababa, Ethiopia; Federal Ministry of Health, Addis Ababa, Ethiopia; Armauer Hansen Research Institute/ALERT, Addis Ababa, Ethiopia; RTI International, Washington, D. C USA; Faculty of Infectious and Tropical Diseases, London School of Hygiene & Tropical Medicine, London, United Kingdom; Neglected Tropical Diseases Support Center (formerly Lymphatic Filariasis Support Center), The Task Force for Global Health, Atlanta, Georgia USA; Center for Neglected Tropical Diseases, Liverpool School of Tropical Medicine, Liverpool, UK

**Keywords:** Integrated, Mapping, Lymphedema, Elephantiasis, Lymphatic filariasis, Podoconiosis, Ethiopia

## Abstract

**Background:**

The World Health Organization (WHO), international donors and partners have emphasized the importance of integrated control of neglected tropical diseases (NTDs). Integrated mapping of NTDs is a first step for integrated planning of programmes, proper resource allocation and monitoring progress of control. Integrated mapping has several advantages over disease specific mapping by reducing costs and enabling co-endemic areas to be more precisely identified. We designed and conducted integrated mapping of lymphatic filariasis (LF) and podoconiosis in Ethiopia; here we present the methods, challenges and lessons learnt.

**Methods:**

Integrated mapping of 1315 communities across Ethiopia was accomplished within three months. Within these communities, 129,959 individuals provided blood samples that were tested for circulating *Wuchereria bancrofti* antigen using immunochromatographic card tests (ICT). Wb123 antibody tests were used to further establish exposure to LF in areas where at least one ICT positive individual was detected. A clinical algorithm was used to reliably diagnose podoconiosis by excluding other potential causes of lymphoedema of the lower limb.

**Results:**

A total of 8110 individuals with leg swelling were interviewed and underwent physical examination. Smartphones linked to a central database were used to collect data, which facilitated real-time data entry and reduced costs compared to traditional paper-based data collection approach; their inbuilt Geographic Positioning System (GPS) function enabled simultaneous capture of geographical coordinates. The integrated approach led to efficient use of resources and rapid mapping of an enormous geographical area and was well received by survey staff and collaborators. Mobile based technology can be used for such large scale studies in resource constrained settings such as Ethiopia, with minimal challenges.

**Conclusions:**

This was the first integrated mapping of podoconiosis and LF globally. Integrated mapping of podoconiosis and LF is feasible and, if properly planned, can be quickly achieved at nationwide scale.

**Electronic supplementary material:**

The online version of this article (doi:10.1186/1756-3305-7-397) contains supplementary material, which is available to authorized users.

## Background

The neglected tropical diseases (NTDs) are a group of more than 17 mostly chronic infectious diseases and related conditions that represent the most common illnesses of the world’s poorest people [1]. Through continued advocacy the significant burden and impact of NTDs has been gaining global attention in recent years [1–3]. International donors, development partners and endemic country governments are allocating resources for the prevention, treatment and elimination of NTDs. To benefit from these resources, endemic countries and implementing partners are required to develop evidence-based plans, which would allow to track progress and show the effectiveness of programme implementation [4, 5].

WHO, international donors and partners have emphasized the importance of integrated --rather than vertical-- control of NTDs [6, 7]. Disease mapping is the systematic collection of georeferenced data to visualise the distribution and prevalence of a disease in space and time [8]. It provides clear information on the geographical distribution of diseases and the population at risk, both of which are important pre-requisites for determining the areas and population to be targeted for treatment and control of NTDs [8]. Integrated mapping of NTDs is a first step for integrated planning of programmes, efficient resource allocation and monitoring progress and impact of control [4, 9–11]. Previous integrated mapping efforts focused on a range of diseases, including trachoma, onchocerciasis, schistosomiasis, soil-transmitted helminthiases and lymphatic filariasis (LF) [9, 10, 12, 13]. Integrated mapping has several advantages over standalone surveys: costs can be reduced by coordinated use of personnel and transport, and co-endemic areas can be more precisely identified than through disease-specific mapping [4, 9, 10, 14, 15]. However, integrated mapping may be logistically intensive and methodologically difficult, because of differences in the target groups to be mapped and sites to be selected according to the ecology of each disease.

There are two principal causes of elephantiasis, or lymphoedema, in the tropics [16]. The most common cause is LF due to the parasitic nematode *Wuchereria bancrofti* (and, in Asia, *Brugia malayi* and *B. timori*), which is transmitted by blood-feeding mosquitoes [17, 18]. The second principal cause is podoconiosis, a form of elephantiasis arising in barefoot subsistence farmers who are in long term contact with irritant red clay soil of volcanic origins [19]. Podoconiosis has significant economic impact; a study in Ethiopia found that podoconiosis halves an individual’s productivity [20]. In addition, the disease is known to be stigmatised with significant social exclusion [21]. It has been estimated that 1 million cases of podoconiosis exist in Ethiopia, but the nationwide distribution has not been clearly defined.

The overall distribution of LF in Ethiopia is not well established. According to a recent review, approximately 30 million people are thought to be at risk of LF and Ethiopia bears 6-9% of the LF burden in sub-Saharan Africa [22]. Despite these huge burden estimates for both LF and podoconiosis in Ethiopia --but for isolated studies and historical market and school surveys-- no nationwide podoconiosis mapping data existed, and only 112 of the country’s 817 districts had been mapped for LF [23]. Ethiopia launched its integrated National NTD Master Plan in May 2013 [24], and LF and podoconiosis were identified as priority diseases along six other NTDs (i.e. trachoma, onchocerciasis, schistosomiasis, soil-transmitted helminthiases, leishmaniasis and dracunculiasis) [24]. Mapping was identified as a critical first step before implementing any operational programming for the control and elimination of these NTDs.

In recent years advances in technology, such as the availability of remote sensed data, application of geographic information systems and mobile technology has enabled rapid mapping of NTDs [4, 25–27]. We conducted the first ever integrated mapping of podoconiosis and LF in Ethiopia using mobile-based technology.

The aims of this paper are to describe the methodology used for integrated mapping of podoconiosis and LF, document the lessons learnt, and to provide recommendations for future, similar mappings efforts.

## Methods

Two focus group discussions (FGDs) of six individuals each and four in-depth interviews (IDIs) were conducted to assess the challenges faced by mapping team members, including enumerators, team leaders and supervisors. All the data were collected by one of the investigators (KD). Flexible interview guides were used to conduct IDIs and FGDs. Audiotapes were transcribed anonymously, and interviews were conducted in Amharic and were translated into English. Data were analyzed manually. Interpretation of the data was informed by experience during implementation of the survey as well as from the analysis of the qualitative data. By providing these details, we hope that investigators in other endemic countries will benefit and adapt the approach to their local context. Beyond disease-specific lessons learnt, we hope the broader issues that arose in our mapping efforts may be helpful to investigators planning similar large scale integrated projects.

### Ethics approval and consent

Ethics approval was obtained from the Institutional Review Board of the Faculty of Medicine, Addis Ababa University (090/11/SPH) and the Research Governance & Ethics Committee of Brighton & Sussex Medical School (11/116/DAV) for podoconiosis mapping, and from Ethiopian Public Health Inistitute (the then EHNRI) and Liverpool School of Tropical Medicine (LSTM) (12.22) for LF mapping. Once the decision to do integrated mapping was made, amendments were requested and approved by each of these committees. Clearance to conduct the surveys was obtained from the Ministry’s of Health Regional Health Bureaus, followed by Zonal Health Offices. The study was explained to each village leader and written consent was obtained to conduct the study in each village. The purpose of the study was explained to all individuals gathered, and the inclusion criteria were explained. The study was then explained to each individual that met the inclusion criteria, and each was asked for consent. Those who provided consent were registered and requested to sign or fingerprint the consent form. Individual written informed consent was obtained from each participant (≥18 years of age). Additionally for those less than 18 years old, consent was obtained from their parents/guardian and the participant themselves provided informed assent. Confirmed *W. bancrofti* infection was treated by co-administration of one tablet of albendazole and ivermectin, as indicated by a dose-pole according to WHO recommendations [28]. For those with lymphoedema, education was given about morbidity management. As part of the LF elimination and podoconiosis control programs, Ethiopia will put in place a morbidity management and disability prevention plan that will include provision of care for patients suffering from lymphoedema and hydrocele [24].

## Results

### Integration of LF and podoconiosis mapping protocols

The mapping was conducted by a consortium of universities and institutions, including the Ethiopian Public Health Institute (EPHI, previously called EHNRI), Brighton and Sussex Medical School (BSMS), the Centre for Neglected Tropical Diseases (CNTD) at LSTM and the Global Atlas of Helminth Infections (GAHI) at the London School of Hygiene & Tropical Medicine (LSHTM). Initially, the mapping of these two diseases was planned separately. BSMS and GAHI-LSHTM were working on mapping podoconiosis and the EPHI and the CNTD/LSTM were working on mapping LF. However, through discussion with the Federal Ministry of Health of Ethiopia, the possibility of integrated mapping was raised. Experts from both mapping groups held a meeting and identified the advantages and disadvantages of integrated versus disease-specific mapping. Reasons in favour of integrated mapping included i) both LF and podoconiosis had been identified as priority NTDs in the National NTD Master Plan of Ethiopia (2013–2015) [24]; ii) the two conditions have similar clinical manifestations and the same target age group; iii) diagnosis of podoconiosis requires exclusion of LF; iv) analysis of the data from the 112 districts already mapped for LF indicated potential distribution overlaps in areas between 1225 and 1698 meters above sea level [29]; v) information from other countries indicated that integrated mapping would lead to cost savings compared to multiple disease-specific mapping exercises [4]; vi) the recently-launched WHO morbidity management guideline recommends the integration of management of these two diseases [30]; and vii) both conditions are known to exist in other countries including Cameroon, Kenya, Uganda and Tanzania, so development of a protocol for integrated mapping might have application beyond Ethiopia.

The challenges identified were i) that experts preferred targeted rather than nationwide mapping for LF, mainly due to resource constraints, ii) the lack of existence of a guideline for integrated mapping of LF and podoconiosis; iii) the absence of clear diagnostic criteria for podoconiosis; iv) the logistical challenges of large field teams; and v) that the institutions had no history of working together.

**Figure 1:**
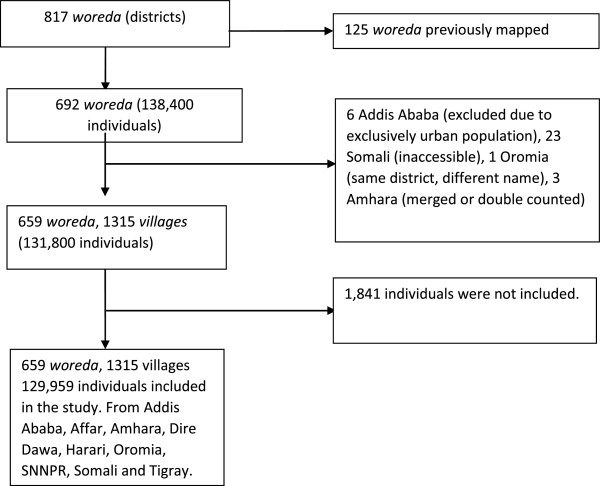
**Sampling framework for LF and podoconiosis mapping in Ethiopia,** **2013.**

Through further discussion, two of these challenges were ameliorated by deciding to use the WHO guideline for LF mapping [11] as the basis for integrated mapping, and through the development of an algorithm for the diagnosis of podoconiosis (Figure 1), which was accepted by both mapping groups. Preparation for mapping then proceeded on an integrated basis.

### Preparatory phase

#### Development of contracts

Contracts were developed between the EHNRI (now the EPHI) and both disease-specific partners (BSMS and CNTD). These set out roles and responsibilities on each side, timelines and budgets. In addition, a Data Sharing Agreement was developed between BSMS and CNTD to delineate the ownership, analysis and publication of data arising from the mapping.

### Development of mapping protocol

Initially, disease-specific mapping protocols were developed. Experts in mapping and epidemiology were involved in harmonizing the two protocols and resolved differences between the two approaches. The planning drew on experience from previous large scale studies in the country such as the Malaria Indicators Survey [31, 32], previous LF mapping [23] and the national tuberculosis survey [32]. After the final protocol was developed, disease specific training manual and standard operating procedure (Additional file 1) for conducting the mapping were developed.

### Procurement and storage of supplies

Supplies and consumables required for the mapping were procured from local markets, while smartphones and immunochromatographic card tests (ICT) were procured internationally. The ICTs (BinaxNOW® Filariasis, Alere, Massachusetts, U.S.) were stored at a central warehouse in EPHI according to the manufacturer’s guidelines, and then transported to the field following the appropriate cold chain procedure. Each team was provided with a cold box and ice packs. On arrival at each district, the team was able to exchange the ice packs with deep-frozen ones at the respective health facility. Subsequent batches of ICTs were distributed to each team during supervisory visits. Due to limited availability of ICT cards at manufacturer level and the incertitude that the project could be accomplished on time, shipment of the ICTs occurred in four batches; this required repeated clearance through customs, and resulted in additional costs and supply chain breakdown.

“*The custom clearance took a lot of time*, *the ICTs were sent in four batches and we have to pass through the clearance process four times*, *each taking 3*–*4 weeks. If all the ICT were shipped together*, *the cost of transport and storage would have been reduced. We were paying for customs on daily basis. Because of the ICT shortage there was a one week interruption of the mapping which incurred cost. I think sending the ICTs in one batch would have avoided all these problems*”. [Coordinator].

### Transport

One local supplier was identified following a competitive bid process. In total, 34 vehicles were hired for the entire project period and 4 additional vehicles for supervision. Each team was provided with one vehicle and travelled together during the data collection period. The supplier was responsible for covering vehicle maintenance and drivers’ allowances. One focal person from the supplier was identified, similarly one person responsible for coordination of the transport was identified on the mapping team. Any communications regarding vehicle and transport issues were dealt with by the focal persons on each side. As far as possible, the same vehicles were used throughout the mapping process, enabling each team to develop a relationship with the driver. Some of the drivers were not willing to drive in challenging areas nor to include individuals selected from the community among their passengers, thinking that it is not their duty.

“*Transportation is key for the success of large scale surveys such as this and the driver is a key person. To reduce disharmony*, *it is important to clearly indicate what is expected from the drivers and include this in the agreement with the suppliers*.” [Team leader].

“*Clear agreement should be signed with car suppliers because some of the drivers were not willing to go to some difficult places. In my view*, *incentivizing drivers could be helpful to get their maximum support*”. [Enumerator].

“*The payment for the vehicle rental was a flat rate*; *all were paid the same price. The payment should be context specific and should consider the distance from the capital and the topography*”. [Team leader].

### Smartphone data collection

Motorola Atrix HD smartphones with an android application were used for data collection, each costing $136 (unit price) [33]. Four forms - ‘Community’, ‘ICT result’, ‘Podoconiosis questionnaire’ and ‘LF questionnaire’ - were interlinked using a unique identifier. The questionnaire interface was developed by experts from the Taskforce for Global Health. The smartphones had touch screen display and exchangeable batteries, which served for 4–5 hours. They used local Wifi and mobile internet services and were linked to a server in Atlanta. After the application and questionnaires were installed, pilot testing was conducted and changes made before the start of the actual work. An internal GPS allowed the direct capture of geographic coordinates.

Data for this study were collected using the LINKS system [33], a mobile application (app) which allows data to be entered on mobile devices running Android and sent through a 256-bit encrypted connection to a centralized cloud-based database server. Eighty Motorola Atrix HD mobile phones used for this project; two per team and 12 spare for any emergency replacements. Hierarchal data were collected using separate surveys for community level information and for individual level information. These surveys could later be linked together to produce a full analytic dataset. The community survey focused on collecting information on the site (region, zone, w*oreda* (district), and *kebele* (sub-district)), but also included population counts and information about community-wide treatment of LF and other deworming activities in the past year. The individual and examination survey included information regarding general demographics as well as an assessment of LF morbidity. The perceptions of the data collectors on smartphone-based data collection are presented in Table 1. A major concern was data ownership, as well as lack of technical expertise at local level to deal with technical and operational challenges of the android smartphones and the LINKS system.

**Table 1:** **Data collectors**’ **perceptions of data collection using smartphones**

Aspect	Perception
Time	Saves time during data collection through automated skip patterns.
	Saves time during entry: paper-based data collection requires double data entry.
	Writing on a smartphone is easier than writing on paper.
Data quality	Some restrictive rules reduced error. For example, it was impossible to enter age less than 15 years.
	The skip pattern reduced error in entering irrelevant data.
Transport and logistics	Easy to carry compared with thousands of questionnaires.
	Reduces duplication, stamping and transportation. Smartphones are handy and easily portable.
Data storage	Send data instantly. However in case of lack of network access data must be stored and could be lost.
	Paper based data are difficult to keep clean.
Communication	Unless you explain to the respondents, they may think that you are playing a game or not fully attending when you are entering data onto a smartphone.
	People are more familiar with paper and would be more comfortable to respond to questions.
Feedback mechanism	Feedback is received on a regular basis, since the data managers at central level have access to the completed data instantly. In paper-based data collection you have to wait until a supervisor comes and collects the questionnaire.
Other concerns	Charging in areas where there is no electricity is difficult.
	Smartphone are costly and may attract robbery.
	Once data are sent there is no room to correct, unless you contact people in the central level.

“*Currently we have an agreement with the server owner about data use. But I would prefer if the server was under our control*….*In the future if we get capacity building training and if we administer the server in country it would be more secure and preferable. In the current situation you feel that you can*’*t control your data*.” [Coordinator].

### Data collector training

Recruitment of the data collectors was conducted through a formal procurement procedure, with clear and specific job advertisements placed in a national newspaper; job requirements included hands-on experience of data collection and previous use of ICT or other rapid test. A training of trainers (TOT) workshop was organized by the BSMS and EPHI team for six trainers. A participatory approach was used to train the trainers on the smartphones, training manuals and testing procedures. Subsequently, a total of 136 health providers were given two days’ classroom-based training (on the mapping protocol, how to operate the smartphones and how to collect data using the android application) and one day field practice in a nearby community. On the first day, all data collectors attended a common plenary session and then breakout session according to their specialty. These individuals were formed into 34 teams each including a health officer, two nurses and a laboratory technician. The TOT training was instrumental in bringing all the trainers on to the same level.

“*The TOT training was important*: *we had thorough discussion among ourselves* [*trainers*], *and this helped us to clear some ambiguities. During the training every trainer was talking the same language*.” [Coordinator].

### The mapping process

Each team was provided with supplies and assigned a vehicle for the entire mapping period. Four days were assigned for mapping two sub-districts. On the first day the district health offices were contacted, the mapping explained, permission obtained and suspected high-risk communities were identified based on review of the health records. An additional four community health workers were recruited in each district to serve as community mobilizers and translators. On the second and third days, data collection was carried out in each sub-district, and the fourth day was used to travel to the next district. In practice, three days per district was usually found to be sufficient, despite the mapping exercise being carried out during the rainy season, which often severely restricted travel.

### Sampling

Two-stage cluster convenience sampling was used. The primary sampling unit for the survey was the *kebele* (lowest level administrative structure, population approximately 5000) (Figure 1). Two *kebele* were selected from each *woreda* (district) based on reported history of lymphoedema cases collected through interviewing the *woreda* health officials, health providers and village leaders one day prior to the survey. Villages within each *kebele* were also purposively sampled. The secondary sampling unit was individuals selected within each village using systematic sampling from a random start point. Mobilization was conducted one day prior to the survey using Health Extension Workers (HEW, community health workers with an average of two attached to each *kebele*). Every adult in the community was informed through house-to-house visits that a survey was to be conducted, and were invited to participate. On the day of the survey, all persons aged 15 years and above living in the selected communities were invited to gather at a convenient point. The study objectives were then explained in the local language, and those willing to participate were asked to form two lines, one of men and the other of women. Fifty individuals were selected from each line using systematic sampling from a random start point, resulting in an overall sample of 50 males and 50 females. Two hundred individuals were therefore tested in each *woreda*. In most villages, it was possible to mobilize all adults in the community and obtain appropriate samples. Individuals were excluded from the study if they had not lived in the *woreda* for at least 10 years, had left the *woreda* for more than 6 months in the year prior to the survey, or did not provide informed consent.

### Data collection

**Figure 2:**
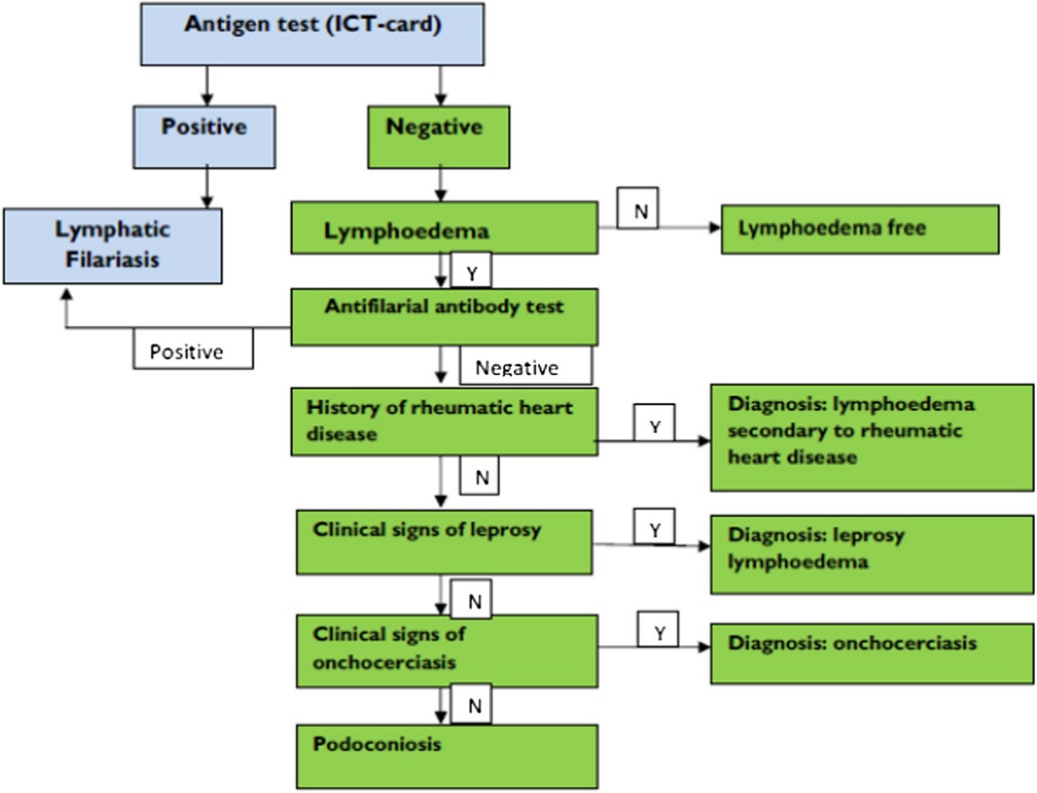
**Clinical algorithm for podoconiosis diagnosis.** There is no point-of-care diagnostic tool for podoconiosis. Currently, podoconiosis is a diagnosis of clinical exclusion based on history, physical examination and certain disease-specific tests to exclude common differential diagnoses. All individuals included in the survey were tested for circulating *W. bancrofti* antigen using an ICT. Those found to be positive, regardless of the presence or absence of lymphoedema, were excluded from further clinical examination for podoconiosis. The common differential diagnoses of podoconiosis are lymphoedema due to LF, systemic disease and leprosya. The differentiation of podoconiosis from LF used a panel approach, including clinical history, physical examination, antigen and antibody tests. The swelling of podoconiosis starts in the foot and progresses upwards, whereas the swelling in LF starts elsewhere in the leg. Podoconiosis lymphoedema is asymmetric, usually confined to below the knees, and unlikely to involve the groin. In contrast, lymphoedema due to LF is commonly unilateral and extends above the knee, usually with groin involvement. In addition to the clinical history and physical examination, an antigen-based ICT was used to distinguish between the two causes of lymphoedema, although the majority of LF patients are also negative for the antigen-based test. To distinguish between podoconiosis and leprosy, clinical history and physical examination was used. Patients were asked if they had been diagnosed with leprosy and physical examination was conducted to exclude signs of leprosy including sensory loss. Onchocerciasis has clear clinical features which can easily be distinguished from podoconiosis. All lymphoedema cases were examined for signs of onchocerciasis. Systemic causes of lymphoedema were ruled out by examination of other organ systems. Hereditary causes of lymphoedema were excluded since they occur at birth or immediately after birth, whereas podoconiosis requires extended exposure to red clay soil.

**Figure 3:**
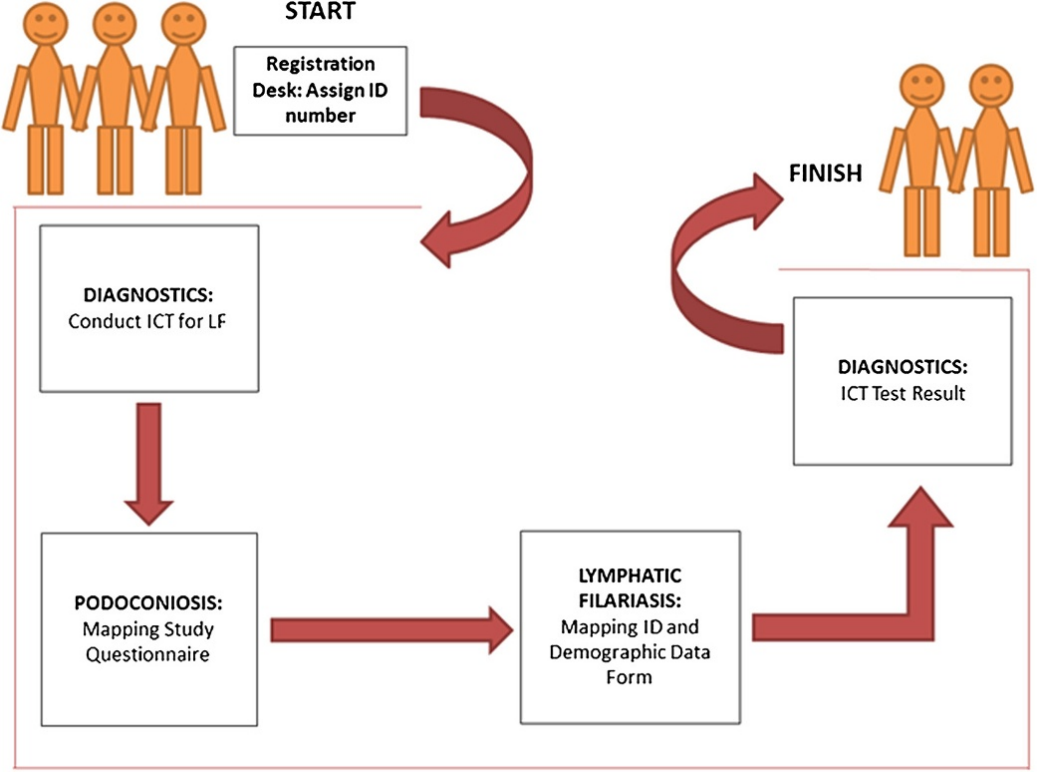
**Mapping survey setup.** Each individual participating in the survey was registered and gave informed written consent. Then they were assigned an in individuals ID and were given a card. The participants retained the card throughout the survey. Then ICT test were conducted, followed by podoconiosis and LF questioner. Finally ICT test results were provided to each individual.

Participants were requested to provide a finger-prick blood sample to be tested for circulating *W. bancrofti* antigen using ICTs. All the participants were tested for ICT, and results were recorded with the individual’s ID number both on the card, and on the smartphone proforma after 10 minutes. In villages where there was at least one ICT-positive individual, all ICT-negative people with lymphoedema were asked to provide 5 ml blood for antifilarial antibody (Wb123 assay) testing at the central laboratory in Addis Ababa. The clinical algorithm used in the mapping process was found to be easily understandable by the data collectors. For individuals with lymphoedema, an algorithm was used to identify possible differential diagnoses of podoconiosis (Figure 2). In this study, a confirmed podoconiosis case was defined as a person residing in the study *woreda* for at least 10 years, with lymphoedema of the lower limb present for more than 3 months for which other causes (i.e. LF, onchocerciasis, leprosy, Milroy syndrome, heart or liver failure) had been excluded. A diagram showing the order of stations used during data collection is shown as Figure 3. In those individuals clinically confirmed to have podoconiosis, duration of illness, family history of similar illness among blood relatives, and disease stage according to the validated podoconiosis staging system [34] were recorded.

“*The algorithm was very clear and there was nothing difficult about it. We were given intensive training before we departed to the field. Because of these reasons it was not difficult to use the algorithm. In case there were some uncertainties*, *the team had a discussion to arrive at a diagnosis*.” [Team leader].

“*The algorithm was supplemented by pictorial presentation of different stages of podoconiosis. Any health workers who had never heard of podoconiosis could easily use the algorithm after the training*.” [Enumerator].

### Field supervision

A team from EPHI, BSMS, CNTD and other partners supervised the data collection. The supervision was intensive during the first two weeks of the start of the project. Experts experienced in both diseases and who had participated in TOT training participated in supervision. During supervision, adherence to the protocol and standard operating procedures were checked. Given the extended experience of the data collectors, adherence to the protocol was found to be very high.

“*The supervision was very important. First the data was collected by smartphone*; *although we have demonstrated the use of smartphone to everyone there were some practical challenges in the field which needed immediate solutions. There were also some practical challenges regarding standard operating procedures*, *which were given solutions at the field level. Particularly during the first three days there were some problems*, *but in subsequent days the mapping continued very smoothly. Personally*, *I was checking adherence on the standard operating procedures*, *I was giving them practical solutions in the field. For example*, *some of the sites were not accessible since the data collection coincided with the rainy season. So we facilitated the use of motorbikes*, *boats and horses*”. [Supervisor].

### Data flow and monitoring

Data were uploaded in real time to an Amazon Elastic Compute Cloud (Amazon EC2) hosted central database which is managed by the Taskforce for Global Health using mobile internet services. Data summarized by district and village could be monitored by two experts given authorized access. This enabled rapid consultation and correction of data in consultation with the field teams. Additionally the national NTD control team leader at the Federal Ministry of Health had access to the interface to observe progress, though this access did not permit data editing. The data collection was conducted between June and October 2013. In total 129,959 individuals in 1,315 communities in 659 districts were mapped over a period of just three months.

### Costs of the survey

According to the planning budgets, the cost of LF-only mapping covering 659districts was $1,212,209, while the budget for podoconiosis-only mapping covering 659 districts was estimated at $1,211,664. The actual financial cost of the integrated mapping of LF and podoconiosis was $1,291,400 – a significant cost reduction through savings in the areas of team training, ICT and supplies, and travel, as described in Table 2. Overall the individual survey costs 1.9 times as high as the integrated survey approach.

**Table 2:** **Budgets and actual costs of LF and podoconiosis mapping in Ethiopia**

Item	LF only mapping (originally budgeted)		Podoconiosis only mapping (originally budgeted)		Integrated mapping (actual expenditures)	
	Description	Amount(US$)	Description	Amount(US$)	Description	Amount(US$)
Training	For 102 data collectors (without 34 podoconiosis nurses)	12,958	For 102 data collectors	12,958	For 102 data collectors + 34 Nurses	17,278
Personnel	For 102 data collectors(central), supervisors, drivers, and local data collectors (without 34 podoconiosis nurses)	350,376	For 102 data collectors(central), supervisors and drivers, and local data collectors ( without 34 nurses for LF)	350,376	For 102 data collectors(central), supervisors and drivers, and local data collectors	419,793
Vehicle rent	For each team (34 teams)	269,100	For each team (34 teams)	269,100	For each team (34 teams)	269,100
Fuel		27,961		27,961		27,961
ICT cards		399,800		399,800		399,800
Mobile phones	44 mobile phones (without 40 mobile phone for podoconiosis data collection)	6,000	40 mobile phones	5,454	84 mobile phone	11,454
Other mapping supplies		81,045		810,45		81,045
Data management		1,667		1,667		1,667
Result dissemination and project execution		18,302		18,302		18,302
Bench Fee		45,000		45,000		45,000
**Total cost**		**1** **,212** **,209**		**1,** **211** **,664**		**1,** **291** **,400**

### Summary of the results

Individual level data were available for 129,959 individuals from 1,315 communities in 659 districts. A total 8,110 individuals with lymphoedema of the lower limb were identified. A total of 139 individuals were found to be positive for *W. banchrofti* antigen with ICT. At least one ICT positive case was found in a total of 89 sub-districts in 75 districts.

## Discussion

We present here detailed practical information on integrated mapping of LF and podoconiosis in Ethiopia. By presenting our approach, we hope to provide important guidance for future integrated mapping of these and other NTDs. Notable features of the work were that we were able to implement a first integrated mapping of LF and podoconiosis as planned without any major challenges. The project received support from the community, district, regional and federal officials. The approach enabled extensive geographical coverage at relatively low cost, which would have been difficult to attain using disease-specific mapping. We hope that our approach may be implemented in other countries where both diseases are endemic. In countries where LF mapping has already been conducted, applying our approach is likely to be beneficial in monitoring LF intervention progress. In countries where there is no LF, but podoconiosis is suspected to be endemic, the approach could be applied to identify areas requiring intervention.

The mapping data were formally presented to the Federal Ministry of Health within one year of the start of the project. The results were disseminated at national level in the presence of stakeholders. The maps generated will be used to inform LF elimination and podoconiosis control programs in the country. The endemic districts identified will be the bases of scaling up interventions, while the population at risk estimations serves as the basis for expanding preventive interventions.

The integrated mapping approach clearly indicated how integrated mapping, international collaboration and mobile technology together enable the conduct of large surveys for NTDs control and elimination. There were several factors that contributed to the success of the project: firstly, the mapping was needs-based - both the national program, international donors and partners needed the data urgently; secondly, the Federal Ministry of Health was an integral part of the planning and implementation of the mapping, which greatly facilitated collaboration from district health offices and other stakeholders; and thirdly, other recent national surveys [26, 35] meant that officials on the ground were familiar with the importance of large scale surveys.

The integrated mapping proved to be an appropriate, well-received alternative to individual disease surveys. It is probable that resources were saved because the cost for adding podoconiosis to the LF mapping only added one more smartphone per team and one more person for data collection and associated tests, such as collection of serum for the antibody test. Parallel efforts and duplication of training, transport, community mobilization, ICT testing, and a database server of two independent disease-specific surveys were avoided. The resources saved from such duplication allowed more districts to be covered, which enabled mapping of all the districts of Ethiopia. In the initial plan, both disease-specific mapping projects were targeting smaller areas where the diseases were suspected. There are several other advantages of the integrated mapping approach beyond those of cost. Many logistic matters were addressed together, such as signing agreements, procurement of supplies, vehicle rental and data management. All these activities would have been duplicated if the individual surveys had been conducted separately. Importantly, the NTD experts were taken away from their daily activities for shorter periods of time.

The application of mobile technology for mapping has shown that it is a highly effective tool in a resource-constrained setting such as Ethiopia. Previous studies have documented that the time spent and the cost of mobile-based data collection was much lower than paper-based data collection [26]. Our planning budget suggested that the cost of data collection and entry was reduced by half when moving from paper-based to mobile-based data collection. In addition, the data were available in real time allowing experts to give prompt feedback to field teams. To overcome the challenges of power shortages, a car adapter was procured and each team was able to charge their smartphones from the assigned vehicle. In addition the mobiles were kept on flight mode during data collection to save power. In areas where there was no mobile network coverage, the smartphones were able to store data form up to 10,000 individuals, making data collection extremely flexible. Throughout the process, only one mobile phone became nonfunctional, and this was because the enumerators mistakenly uninstalled the program. Each team was provided with 500 paper based questionnaire in case of any problems with the smartphones.

Strong community mobilization was another important component of the mapping. The support obtained from federal, regional and district level officials was instrumental in implementing the project without significant challenge. The involvement of village level support team, including HEWs and *kebele* leaders, was vital for community mobilization. Given the strong link and trust built between the HEW and the community, mobilization was achieved in a relatively short period of time. Identifying community level personnel such as HEWs is important to build trust between data collectors and the community, and to achieve adequate mobilization and consent for participation. HEWs are uniquely positioned to estimate the number of adults in the community and conduct house to house mobilization [36, 37]. The consent process is also worthy of a mention: written consent was thought to be appropriate by community leaders before the start of the community mobilization. In some communities, community leaders were consulted in the decision to participate or not. Community leaders were found to be catalysts for participation in the study.

Despite initial concerns surrounding the podoconiosis algorithm used in the current study, it was easily understood by the enumerators. Supervisors witnessed a high level of accuracy of the use of the algorithm by the data collectors. In cases where there were doubts, discussion was held among the team. Previous studies have indicated that health workers can easily identify podoconiosis from other causes of lymphoedema in endemic districts [38]. However, in the current study, the clinical algorithm was used in areas where podoconiosis is not endemic or potentially co-endemic with LF. The combination of clinical history, physical examination and blood tests were used to reach a diagnosis. Although easily understood by the enumerators, at times the procedure was found to be lengthy and tedious: further refinement of the algorithm is important. This could be achieved through evaluating the predictive diagnostic performances of individual signs and symptoms of podoconiosis.

The skills acquired by through the integrated mapping of these two diseases are highly transferrable to other disease mapping exercises. The smartphones used for the mapping were provided to EPHI, and are currently being used for mapping STH and schistosomiasis. We have trained some 136 health workers to use smartphones in mapping and these skilled enumerators are available for future mapping activities. Integrated mapping has led to further integration of these two diseases, for example the development of an integrated morbidity management guideline for LF and podoconiosis, and the inclusion of an indicator (the number of lymphoedema cases segregated by cause and age) in the routine national health management information system (HMIS).

### Challenges

Although the mapping project was successfully completed in a very short period of time, it was not without obstacles. First, bringing two independently planned projects together resulted in several challenges, since the two projects were initially intended to cover different geographical areas, and the planned sample size and methodology were different. This was partly due to the absence of an integrated mapping guideline for these two diseases, and partly to this being the first nationwide mapping conducted for podoconiosis. Challenges also arose in relation to contracts and agreements: organizational cultures differ, as do expectations and formats for such agreements, so harmonizing these multi-organizational agreements took considerable time (a minimum of 3 to a maximum of 8 months). In addition, funding was not available at same time among different partners. Second, although the use of smartphones was a key strength of the mapping, technical support was provided remotely by a team from Task Force for Global Health. During the pilot phase some inconsistencies in the flow of the questionnaires were identified which needed immediate solutions. Solving simple technical problems took time due to the limitations of virtual communication. Third, the server for the data was hosted outside the country, initially giving rise to concerns over data ownership (Table 3). Fourth, the ICTs were shipped in four batches, which led to unnecessary cost, waste of time and interruption of data collection for a week.

**Table 3:** **Challenges and solutions taken during the mapping**

Challenges	Solutions taken
Batteries running out of charge	Charge all the chargers after work and prepare them for the next day. Use of car charger in areas where there is no electricity. In a few cases where charging was not possible, paper based data collection were conducted for one day, while the other team members charged the smartphones in nearby towns.
Inability to edit once data is submitted in the smartphone.	Communicate with the central team to discuss any errors and edit the data promptly.
Lack of network	Store the data in the smartphone and transfer when there is access for internet.
Community mobilization	Discuss the best time for the community for mass gathering, such as early in the morning or late in the afternoon. Whenever appropriate, use holidays.
Inaccessibility (some districts during rainy season)	Use alternative transport such as motorbikes, boat or horses. In areas where no other possibilities existed, walking was the last resort.

## Conclusion

To achieve the London Declaration of 2020 targets and the WHO road map for NTDs [7], rapid mapping is very important. Integrated mapping of podoconiosis and LF in Ethiopia was conducted at a large scale in a short period of time. The approach is the first of its kind and provides important lessons for co-endemic or podoconiosis-endemic countries. Strong in-country leadership, international collaboration and use of mobile technology contributed to the success of the exercise. The approach reduced costs, expanded geographical coverage and sped up the availability of data for decision makers. Data were formally presented to the Ministry of Health within one year of the start of the project, and will be used to inform national control and elimination programs.

## Authors’ information

Joint senior authors “Maria P. Rebollo, Gail Davey”.

## Electronic supplementary material

Additional file 1: **Study protocol and survey manual.** (PDF 3 MB)
