# Supplementary material for: Integrated mapping of lymphatic filariasis and podoconiosis: lessons learnt from Ethiopia
Source: Parasit Vectors. 2014 Aug 27;7:397. doi: 10.1186/1756-3305-7-397 (PMC4153915; doi:10.1186/1756-3305-7-397)
Supplement: Supplementary file 1 — Additional file 1: Study protocol and survey manual. (PDF 3 MB) [file 13071_2014_1565_MOESM1_ESM.pdf]

Mapping of Lymphatic Filariasis and Podoconiosis in Ethiopia Field Protocol

2013

Federal Democratic Republic of Ethiopia
Ministry of Health

wellcome trust

UKaid from the British people

THE END FUND ENDING NEGLECTED DISEASES

Ethiopian Public Health Institute (EPHI), Brighton and Sussex Medical School(BSMS), Addis Ababa University, Armauer Hansen Research Institute (AHRI), the Liverpool School of Tropical Medicine

And

The Ethiopian Federal Ministry of Health

Contents

Title: Mapping of Lymphatic Filariasis and Podoconiosis in EthiopiaError! Bookmark not defined.

The Need for LF and Podoconiosis Mapping 8

Objective of the Study 9

Study Area 10

Materials and Methods 12

Sampling strategy 15

Study participants 18

ICT-Card: Diagnosis of *W. bancrofti* infection 19

GPS data: 20

Data entry and analysis: 21

Ethical considerations: 21

Expected output of the Study 22

Significance of the study 22

Dissemination of Results and Ensuring the Implementation 22

Monitoring/Evaluation of the studies and Action plan with Time. 23

Research Team 24

Reference 26

Annex 1: tentative time line and deliverables 28

Annex 2: Information sheet 28

Annex 3: Healthy volunteer consent form 28

Annex 4: Questionnaire 28

Annex 5: Training manaul LF 28

Annex 6: Training manaul Podoconiosis 28

Summary

Lymphatic filariasis (LF) and podoconiosis are Neglected Tropical Diseases, these two cause tropical lymphoedema that results in range of temporary and permanent disabilities. Both of the diseases are associated with the disfigurement of grossly swollen limbs and results in stigma for people suffered from it.

The World Health Assembly (WHA) passed a resolution in May 1997 calling for ‘the elimination of lymphatic filariasis as a public health problem’. In consequence, the WHO started a global program, Global Alliance for the Elimination of LF (GPELF). The GPELF initiated by the WHO recommending that all of those who live in at risk communities be treated orally, once a year, with an appropriate two-drug combination.

Early 2012 International Podoconiosis Initiative was launched with the vision of “elimination of podoconiosis in our life time”. In Ethiopia prevention of the disease through promotion and distribution of shoe for school age children and simple lymphoedema managements are showing promising results. To kickoff a global elimination of podoconiosis identifying endemic countries and mapping the distribution of the disease is a pre requisite.

While efforts to eliminate LF and control of podoconiosis are continuing, few African countries have yet to complete mapping the geographical distribution of the LF and none of podoconiosis endemic countries completed mapping. Both LF and podoconiosis are endemic in Ethiopia and were recently included in the National Master Plan (2012-2015) for NTDs, yet control of these diseases at national or sub national level is still at its infancy. The Ethiopian Federal Ministry of Health (FMoH) has recognized LF and podoconiosis as priorities for control, and indicated that a coordinated nationwide mapping is the first step. So, this survey aims to generate a complete map of LF and podoconiosis distribution in Ethiopia.

The project is funded by DFID, The Wellcome Trust and The End Fund.

Introduction

There are two principal causes of elephantiasis, or lymphedema, in the tropics[1] . The most common cause and a significant public health problem is lymphatic filariasis due to the parasitic nematode Wuchereria bancrofti (and, in Asia, Brugia malayi and B. timori), which is transmitted by mosquitoes[2]. The second principal cause is podoconiosis: a form of elephantiasis arising in barefoot subsistence farmers who are in long term contact with irritant red clay soil of volcanic origins [3].

LF is endemic in 73 countries with an estimated 120 million infected and 1.34 billion live at risk of infection. Approximately 40 million people suffer from the stigmatizing and disabling clinical manifestation of the disease, of which 15 million with lymphedema (elephantiasis) and 25 million men with urogenital swelling, principally scrotal hydrocele [2,4].The estimated disability life year's burden due to LF is 5.55 million; in Africa alone, LF causes almost US $ 1 billion in yearly losses, of which more than 80% is due to disability in men with hydrocele. Due to the fact that the lag time between infection and clinical symptoms can be more than 10 years, new manifestations of LF will appear, even when transmission is eliminated[5]. Indeed, it probably causes the loss of more disability-adjusted life years (DALY) than any other communicable parasitic disease that has a major social and economic impact[6].

Podoconiosis (endemic non-filarial elephantiasis) is a non-infectious geochemical disease arising in barefoot subsistence farmers who are in long-term contact with irritant red clay soil of volcanic origins. The disease causes progressive bilateral swelling of the lower legs. Mineral particles absorbed through skin are taken up into macrophages into the lymphatic system and result in an inflammatory process leading to fibrosis and obstruction of the vessels. This leads initially to swelling of the foot and the lower leg, which progresses to elephantiasis: gross lymphoedema with mossy and nodular changes of the skin[7]. Podoconiosis affects some 4 million people in Africa, Latin America, and a few areas of Asia. It is found in more than ten countries across tropical Africa where irritant soils have been generated by environmental conditions of high altitude (>1,000m)

and high annual rainfall (>1,000mm), and are farmed by very poor people who cannot afford shoes or water[7].

Podoconiosis has significant economic impact, according to a study in Ethiopia it was found out that the disease results in productivity loss. Total direct costs of podoconiosis amounted to the equivalent of US$ 143 per patient per year. Total productivity loss for a patient amounted to 45% of total working days per year, and in a zone of 1.5 million people, the total overall annual cost of podoconiosis was calculated to exceed US$ 16 million per year[8]. In addition the disease is known to be stigmatized with significant social exclusion[9].

LF is still on the rise with continued increases in infection rates in tropical and subtropical areas; the prime contributing factor being change in environment and unplanned rapid urbanization which creates breeding sites of Vectors[10]. The overall prevalence of LF in Ethiopia is not well established. According to a recent review it is estimated that 30 million people are at risk for LF and Ethiopia bears 6-9% of the LF burden in Sub-Saharan Africa[11]. Data from sporadic field surveys in endemic focal areas and from hospitals as self-reported cases indicate that the prevalence of LF seems much more than in any other African country. Prevalence from 112 woredas predominantly endemic for LF was 3.7% with high geographical clustering and variation in prevalence (ranging from 0% to more than 50%). According to the study 34 (30.4%) of the 112 *woreda*s, with a population of 1,547,685 in 2007, were found to be endemic for LF [12]. A survey based on night blood to confirm infection in two communities adjacent to Baro River near Gambella[13] found an overall prevalence of 20.7%. This is an average of the male rate (23.7%) and the female rate (18.5%). The study covered 90% of the population in Tektak and Ketch. Infection densities varied between 40 and 1540 microfilariae (mf) per ml of blood among the infected, giving a geometric mean intensity of 309 mf/ml of blood which was much more pronounced in females than in males. In males, 20.3% had hydrocoele and this condition was noted above the age of 35 years. About 40% of those with hydrocoele had microfilaremia. Groin gland enlargement was

recorded in 40.0% of the examined. Hailu, A[14] attempted to piece together information based on the few studies on lymphatic filariasis in Ethiopia. He indicated that the first written report on LF relates to a patient who resided in "…an area endemic for onchocerciasis [thus], diminishing the reliability of the report". Studies point to the existence of the disease in the former provinces of Keffa, Illubabor, Wellega and Gamo Gofa. They all concluded that LF is endemic to western and south western Ethiopia. "This conclusion was based on the documentation of clinical cases described as Elephantiasis scrota (scrotal elephantiasis), Elephantiasis vulvae (vulval elephantiasis), Elephantiasis penis (elephantiasis of the penis) and Elephantiasis mammae (breast elephantiasis)".

Morrone etal[15] conducted an epidemiological survey of LF in the northern region of Ethiopia, between January 2005 and January 2007 and found that 23 patients observed in the Italian Dermatological Centre (IDC) in Mekelle, capital of Tigray. All patients presented acute or chronic episodes of lymphadenitis of the groin or irreversible elephantiasis of the lower limbs or scrotum. Bloods film evidenced the presence of microfilaraemia, indicating possible complications that need a further closer examination. This underlines that LF is not restricted to the western and south western parts only. And recently mapping on the distribution of the disease is done in south western, western and north western part of the countries and about 25% of the districts surveyed were identified endemic for LF

Table 1. Summary of studies conducted on lymphatic filariasis epidemiology in Ethiopia

**Study**	**Year of publication**	**Sample size**	**Area of study**	**Percentage of positives or cases found**
Jemaneh L & Kebede D.	1995		Tektak and Ketch, Gambella	20.7%
Shiferaw et al	2012	11,685 individuals	125 villages (112 *woreda*s) of western Ethiopia	3.7% (ranging from 0% to more than 50%)
Morrone et al.	2007	23 cases in hospital	Mekele	23 cases
Urge et al.	2005 (unpublished)	1114	Nanfilo *woreda*	7.5%

Table 2. Podoconiosis prevalence surveys conducted in Ethiopia between 1969 and 2012

**Publication year**	**Place of study**	**Number of individuals sampled (prevalence)**	**Data points**	**Type of survey**
1969 [16]	Multiple sites	247,908(2.72%)	56	Market survey(visible leg swelling)
1973 [17]	Multiple sites	13,138(2.77%)	26	School enquiry
1987 [18]	Ocholo, Southwest Ethiopia	2689(5.4%)	1	Community based(physical examination)
1992 [19]	Gera & Didessa, Western Ethiopia	416(7.5%)	2	Community based(Physical examination)
1997 [20]	Pawe Northwest Ethiopia	1,900(7%)	1	Community based(Physical examination and microscopic examination of midnight sample)
2003 [21]	Wolita zone, Southern Ethiopia	33,678(5.46%)	7	Community based(physical examination)
2011 [22]	Gulisso west Ethiopia	38,420(5.2%)	1	Community based(physical examination)
2012 [23]	Midakegni	1,656(7.4%)	1	Community based(physical examination)
2012 [24]	Debre Elias and Dembecha Northern Ethiopia	50,620(3.3%)	2	Community based(physical examination)
2012 [25]	Western Ethiopia in 112 Woredas	11,249(4.6%)	133	Community based(ICT card)
Total		401,646(3.4%)	229	

It is estimated that Ethiopia bears one fourth (1million) of podoconiosis cases in the world[3]. According to a recent review the prevalence of podoconiosis stands at 3.4 %(predominantly data from high prevalence areas) in the country with varying prevalence ranging from 0 to 48%. The disease is widely distributed in the highlands of Ethiopia extending from north to south of the country. High prevalence areas are characterized by mean altitude >1500masl, temperature between 19-21°C, mean annual rain fall >1500mm and mean annual precipitation >130mm[26].

GIS and Remote Sensing is becoming very important for capturing landscape epidemiology in cost effective and efficient manner, because it enables high order

analysis of environmental variables related to the diseases incidence. Disease mapping has been greatly enhanced by the use of geographical information systems (GIS) and remote sensing (RS) over the past two decades. GIS has enabled data to be georeferenced, stored, extracted, integrated in new ways and displayed by the user [27]. The availability of accurate and current maps with clear geographical distribution will leads to cost-effective interventions[28].

The Need for LF and podoconiosis Mapping

Podoconiosis and LF are examples of NTDs with the greatest potential for elimination as a public health problem [29]: Podoconiosis is preventable if shoes are consistently worn, and early stages can be successfully treated using a simple lymphoedema regimen [30]. While one million people are estimated to be affected with podoconiosis in Ethiopia, and a further 19.2 million at risk[11], control efforts are hampered by a lack of information on geographical distribution – aside from recent isolated studies, the only previous mapping of this disease was based on podoconiosis cases from market and school surveys in 1970s [31].

LF is a major cause of permanent long-term disability affecting 120 million people in Asia, Africa, the Western Pacific and some parts of Americas. A single dose of diethylcarbamazine (DEC) and ivermectin, have been shown to be effective in reducing microfilaraemia [2]. Before mass drug administration (MDA) can be launched, a country must demonstrate that the disease threshold for public health intervention, as established by the World Health Organization (WHO), has been surpassed. This can be achieved through reviewing the available data and conducting surveys to fill in the gaps. In Ethiopia only a few studies have been conducted in the western part of the country. More recently, mapping of LF has been conducted in the western part of the country. No systematic surveys on LF have been conducted in other parts of the country. Moreover, LF was identified by the Ethiopian NTD Master Plan as one of the diseases targeted for elimination. Current WHO policy is to achieve elimination of infection in humans mainly through combination drugs in large populations, complemented by vector control and

morbidity management and disability prevention. Mapping is therefore an essential 1st step toward elimination planning.

Mapping distribution of LF and podoconiosis is crucial for a number of reasons. Areas without risk of the two diseases need to be identified, while endemic areas need to be delineated and integrated into the elimination and control program. It is also important to define the size of the population at risk of LF and podoconiosis in the country, both enabling effective planning and careful use of scarce resources.

The need for coordinated mapping

Both LF and podoconiosis are identified as priority NTDs in the national NTD master plan of Ethiopia (2012-2015), together with other six diseases, indicating the attention given to these two forms of elephantiasis. The master plan identified mapping of the two diseases as a major priority for decision making in control of the diseases. The other important reason for coordinated mapping of the two diseases is that they have similar clinical features and diagnosis of one would require exclusion of the other. Preliminary analysis of the existing data on LF and podoconiosis indicated that there are potential overlaps of the two diseases between the altitudes of 1225 to 1698 masl[26]: in these areas, there is a need to confirm the cause of lymphoedema. Studies conducted in LF endemic countries have indicated that a considerable proportion of lymphoedema cases test negative by antigen test, suggesting that further investigations will be necessary to distinguish the two main causes of lymphoedema. Both of the diseases have the same target group (individuals >15 years old) for mapping, and both are co-endemic in a number of tropical African countries including Cameroon, Uganda, Kenya and Tanzania, so the lessons learnt in joint mapping of the two disease in Ethiopia may serve other endemic countries. Conducting multiple surveys in the same country can be costly and burdensome to national disease programs.

Objective of the Study

- To map the distribution of LF in unmapped districts in Ethiopia

- To map the distribution of podoconiosis in districts covered by the LF study.
- To develop a endemicity map of LF in Ethiopia
- To develop a endemicity map of podoconiosis in Ethiopia
- To estimate the population at risk for LF in Ethiopia
- To estimate the population at risk for podoconiosis in Ethiopia

Study Area

The study will cover those unmapped woredas (districts) indicated in the map below **(Fig.1).** Ethiopia has a federal system of administration with nine regional states and two city administration councils. The Regional states/city administrations are sub divided into Zones, districts (Woredas) and kebeles (subdistrict) hierarchically. In total there are 817 woredas in Ethiopia. A woreda/district is the country's basic decentralized administrative unit and has an administrative council composed of elected members. The 817 woredas are further divided into about 16,253 *kebeles*. There are three broad ecologic zones in Ethiopia, that follow the topography: the "kola" or hot low lands are found approximately below 1500 meters, the "weyna dega" between 1500-1750 meters and the "dega" or the cool temperate highlands above 1750 meters. It is estimated that about 75% of the land in Ethiopia is malarious and 65% of the population is living in this area.
112 woredas were mapped for LF in 2008 and the results are shown in the map below. 13 districts are planned to be mapped by Addis Ababa University.

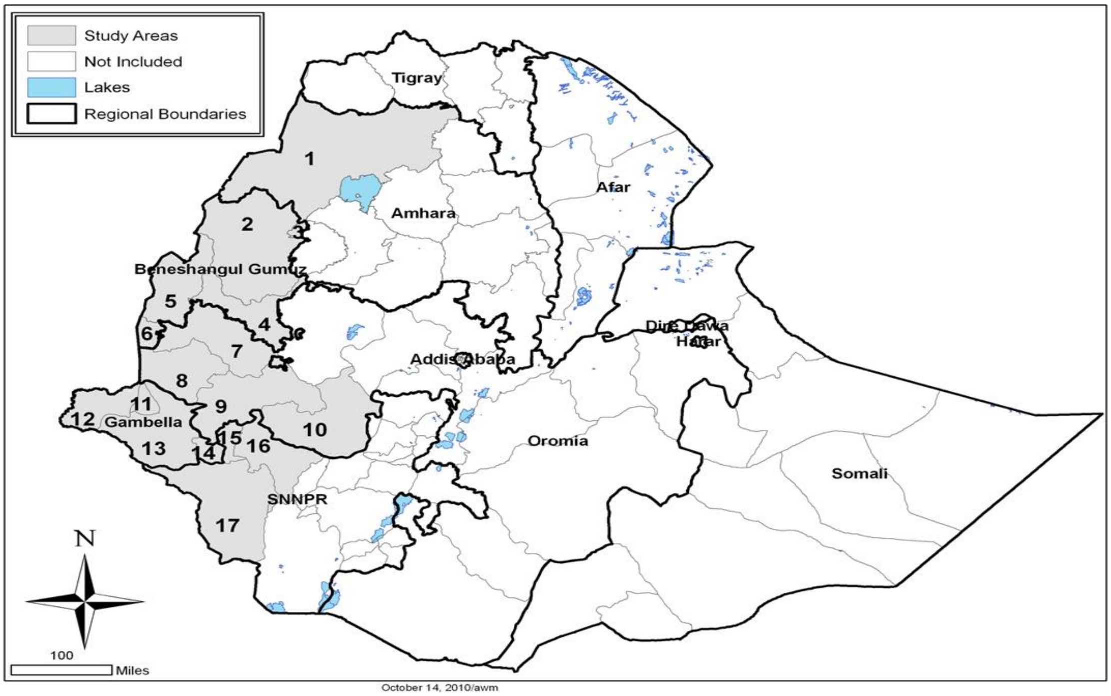

Figure 1 District level administrative division and status of LF mapping in Ethiopia

/

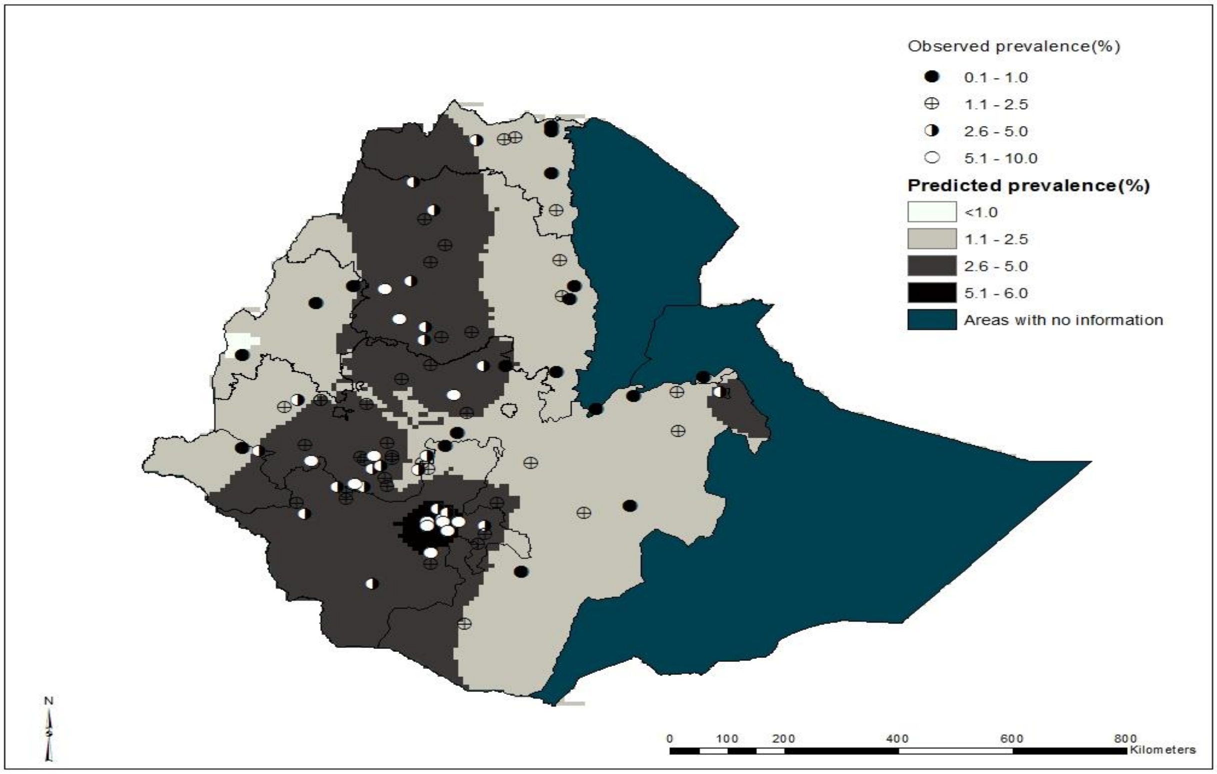

Figure 2 Map of estimated prevalence of podoconiosis in Ethiopia using kriging. The map shows the presence of a spatial pattern which correlates with environmental factors.

Materials and Methods

1. Study area and period

This study aims to complete the already started mapping of LF in Ethiopia. The study will cover Afar, Amhara, Tigray, Oromia, Somali, Dire Dawa, SNNPR and Harari regions of Ethiopia. The study will be conducted from January – June 2013.

Table 3. The woredas and Zones of Ethiopia and the number of woredas mapped for lymphatic filariasis

Study zones	Number of Woredas	No of Woredas mapped	Remark
**Tigray**	**47**	**0**	
North Western	8	0	
Central Tigray	13	0	
Eastern Tigray	9	0	
Southern Tigray	11	0	
Western Tigray	4	0	
Mekele Special	2	0	
**Afar Region**	**33**	**0**	
Zone 1	8	0	
Zone 2	8	0	
Zone 3	7	0	
Zone 4	5	0	
Zone 5	5	0	
**Amhara Region**	**153**		4 woredas were mapped (Metema, Quara, Tacharmach ihoo and West Armachiho)
North Gonder	23	4	
South Gonder	12	0	
North Wello	12	0	
South Wello	22	0	
North Shewa	24	0	
East Gojam	18	0	
West Gojam	15	0	
Waghimra	7	0	
Awi	11	0	
Oromia	7	0	

**Study zones**	**Number of Woredas**	**No of Woredas mapped**	**Remark**
Bahir Dar Special	1	0	
Argoba Special Woreda	1	0	
**Oromia Region**	**308**	**54**	
East Wollega	18	0	
Ilubabor	24	19	19 woredas were mapped
Jimma	19	13	13 woredas were mapped
West Shewa	19	0	
North Shewa	15	0	
East Shewa	15	0	
Arsi	26	0	
West Harerge	16	0	
East Harerge	22	0	
Bale	20	0	
Borena	14	0	
South West Shewa	13	0	
Guji	15	0	
Adama Special Zone	1	0	
Jima Special Zone	1	0	
West Arsi	14	0	
KelemWollega	11	10	10 woredas were mapped
Horogudru	10	0	
Finfine Zuriya	14	0	
West Willega	21	12	12 woredas were mapped
**Somali Region**	**72**		
Shinile	7	0	
Jijiga	8	0	
Gegehabur	10	0	
Warder	5	0	
Korahe	6	0	
Fik	10	0	
Gode	10	0	
Afder	10	0	
Liben	6	0	
**Benshangul G Region**	**21**	**17**	
Metekel	7	4	4 woredas were mapped
Assosa	8	7	7 woredas were mapped
Kamashi	5	5	all woredas were mapped

Study zones	Number of Woredas	No of Woredas mapped	Remark
Mao Komo special wereda	1	1	one/only woreda was mapped
**SNNPR**	**158**	**26**	
Gurage	15	0	
Hadiya	11	0	
Kembata	8	0	
Sidama	22	0	
Gedeo	8	0	
Wolayita	15	0	
South Omo	9	0	
Sheka	5	5	all woredas were mapped
Keffa	11	10	10 woredas were mapped
Gamogofa	17	0	
Bench Maji	11	11	all woredas were mapped
Special woredas	5	0	
Dawro	6	0	
Silti	9	0	
Hawassa city	1	0	
Segen	5	0	
**Gambella Region**	**13**	**11**	
Agnewak	6	5	5 woredas were mapped
Nuwer	5	5	all woredas were mapped
Mezenger	2	0	
Etang Woreda	1	1	one/only woreda was mapped
**Harari All Woreda**	**9**	**0**	
**Dire Dawa**	**8**	**0**	
**Addis Ababa**	**10**	**0**	
**Total**	**833**	**112**	

1.1. Study design

The survey is a population based cluster cross sectional study

1.2. Source population

The source population constitutes all individuals living in the selected woredas/districts of Ethiopia

1.3. Study population

Individuals > 15 years old randomly selected from the residents in the selected village will be the study population

1.4. Inclusion and exclusion criteria for study subjects

1.4.1. **Inclusion criteria:** To be included in the sample, people should have lived in the area for at least ten years, and be greater than or equal to 15 years old

1.4.2. **Exclusion Criteria:** individuals who lived in the area for less than 10 years, or who left the area for at least 6 months will be excluded from the study. The following will be excluded from the study:

- Terminally ill patients who cannot respond for the interview.
- Patients with a mental health condition that makes interview difficult
- Severely sick (eg. high fever)

Sampling strategy

All woredas that have not been mapped previously or planned to be mapped will be included in the study. In total 692 woredas will be mapped.

The methods that are going to be applied for the project will be composed of two broad categories. Namely: data collection phase and data analysis phase.

Figure. Sampling framework

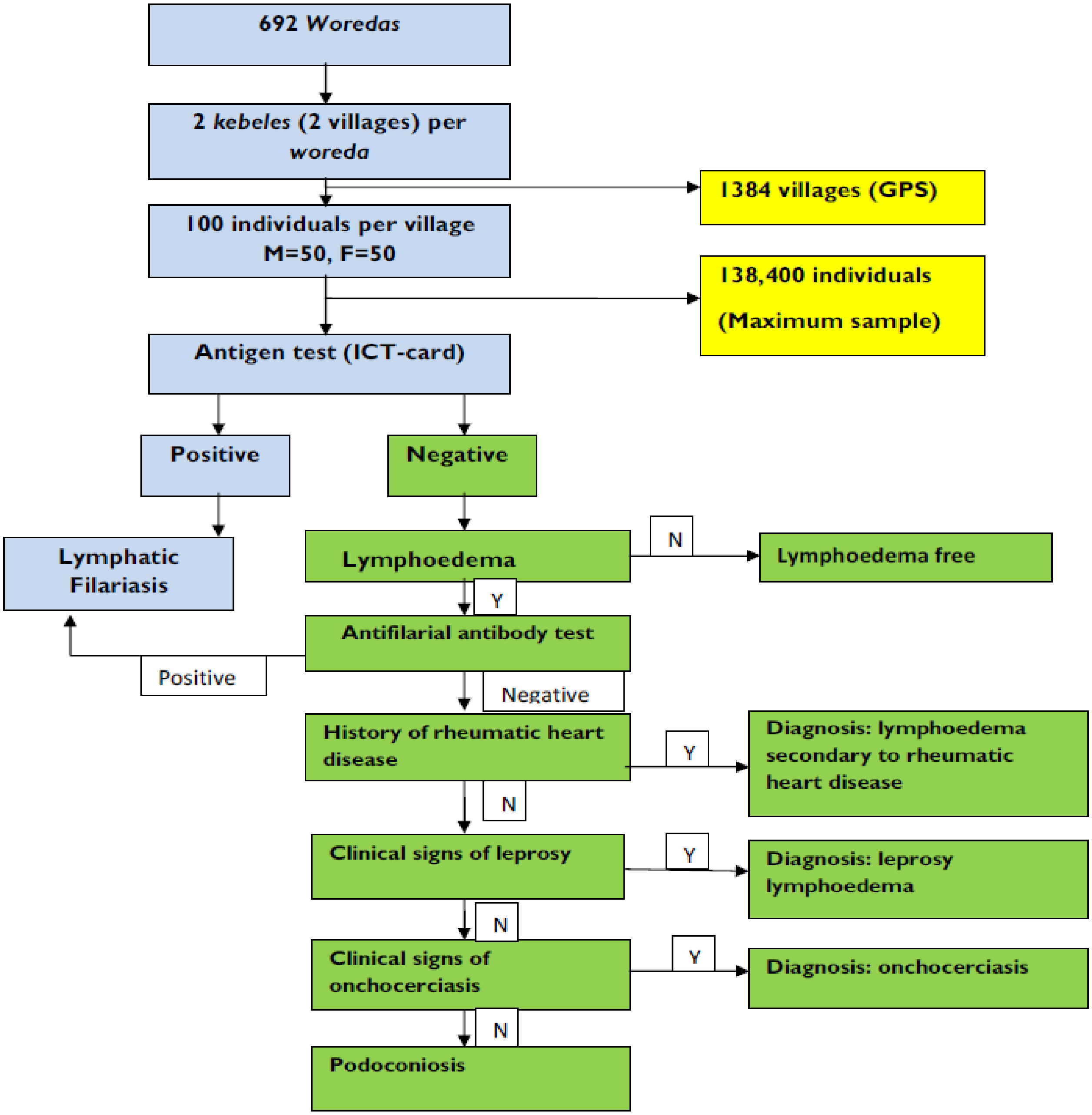

The data collection phase will include:

- All districts (Woreda) that have not been mapped before will be included making a total of 692. From each woreda, 2 kebeles will be selected. The selection of the kebeles will be guided by the health care facilities based on the presence of clinical cases (hydrocele or lymphoedema) from the selected Kebeles;

communities (gotts) will be selected based on the previous knowledge clinical cases.

- Sensitization training will be given to the *gott* representatives and health extension workers for the selected gotts.
- At least 2 communities will be selected in each Woreda. 100 volunteers will be included (50 women and 50 men) older than 15 years old. 100 participants will be tested using ICT-Card in each gott. When conducting the survey if 20 positives are found after testing the first 50 individuals the survey can stop and the gott will be consider to be endemic.

- In some *woreda*s, the *Woreda* Health Office may have additional information, such as frequent reports of hydrocoeles or lymphoedema that strongly suggests that filariasis or podoconiosis is endemic in specific villages. Before undertaking the survey, the survey team will visit the *Woreda* Health Office and seek information, if available, on the villages which are most likely to be endemic. The 2 villages most likely to be endemic according to the *Woreda* Health Office will be selected. Initially we thought adaptation of the WHO 2000 mapping guideline through selection of one village randomly and the other as a check village, however through discussion with experts from WHO and we opted to select two villages with anecdotal cases of hydrocoeles or lymphoedema. This method will increase the yield of detecting high-risk communities for both LF and podoconiosis. .

Ta*ble 4.* Administrative structures and numbers in Ethiopia

SN	Regions	Number of Zones	Number of Woredas	Number of Woredas Mapped for LF	Woredas not map*ped*
1.	Addis A*baba*	*1*	10	0	10
2.	Afar	5	33	0	33
3.	Amhara	11	153	4	149
4.	BenishangulGumuz	3	21	17	4
5.	Dire Dawa	2	8	0	8
6.	Gambella	3	13	11	2
7.	Harari	1	9	0	9
8.	Oromia	18	308	54	254
9.	SNNPR	15	158 (5sp)	26	132
10.	Somali	9	72	0	72
11.	Tigray	5	47	0	47
	Total	70*	833**	112	721

* *Rece*ntly, most of the large cities in the country have been delineated as special zones, increasing the total number of zones in the country to 94.

** Some woredas ha*ve rec*ently split, leading to a slight increase in total number of woredas from 817 in official reports in 2011

For podoconiosis, Ethiopia will be divided into three strata: high risk, medium risk and no information (low risk). To detect a prevalence of 0.5% with 0.25% precision (i.e. to produce a 95% confidence interval between 0.25% and 0.75%) with 80% power, and assuming a design effect of 2 and 10% non-response rate, we calculated that in each stratum, 6,728 individuals from 135 villages should be included, thus a total of 20,184 individuals from 405 villages will be included in the survey. Based on this sample size, the LF sampling will also be sufficient to address the objectives of podoconiosis mapping.

Study participants

After explaining the purpose of the study to the village leaders and obtaining their permission, all resident adults of the village (over 15 years old) will be asked to gather at a convenient point. The study will be explained in local language, and those willing to

participate will be asked to form two lines, one o males and another of females. Fifty individuals will be randomly selected from each line using systematic sampling, resulting in an overall sample of 50 males and 50 females. Data will be recorded on the data sheets as soon as the participant is selected. Data sheets have been developed by CNTD for this activity.

Identification of participants

A simple questionnaire will be used to record age, sex, education, occupation, place of residence and the test result of ICT of all individuals involved in the study. For those individuals with lymphoedema, a simple checklist will be used to examine for possible differential diagnosis of podoconiosis. In those clinically confirmed to have podoconiosis, duration of illness, shoe wearing practice and disease stage will be recorded. The tool will assess the progression of the swelling, and check for a history of rheumatic heart disease. In addition, information from physical examination including preservation of sensation in the toes, clinical signs of leprosy or onchocerciasis, and groin involvement will be recorded.

Screening of the study population for the main signs and symptoms of LF, i.e. elephantiasis or lymphoedema in both sexes and hydrocele in men, will be performed in a health facility setting (health center/health post) by trained nurses. All men who reported having genital symptoms of LF and agreeing to genital examination will be assessed by a nurse.

Diagnosis of *W. bancrofti* infection

A. **ICT-Card:**

On arrival of the ICT cards in Ethiopia the stock will be tested using LF antigen.

After obtaining informed consent from the participant, the test will be performed by trained laboratory technicians, according to the manufacturer's instructions (SOP in annex). The patient's third or fourth finger will be cleaned with 70% alcohol and punctured using a sterile lancet. The initial sample of blood will be removed using a cotton swab, and sufficient fresh blood will be obtained to fill a 100-µl capillary tube.

The blood will be transferred from the capillary tube to the pad on an immuno-chromatographic test (ICT) card and the card will be sealed.

The result of each ICT card will be read at 10 minutes exactly, not before, and not after. A positive result shows two pink lines which appear in the card's window, and a negative result will show a single line. The test will be repeated if the control line is not shown and if both the control and test line show color other than pink. Test results with the individual's ID number will be recorded both on the card, and on each individual's data sheet.

B. Diagnosis of podoconiosis

In areas where there is at least one positive immuno-chromatographic test (ICT), all patients with lymphedema but a negative immuno-chromatographic test (ICT) will be asked to provide 5ml of blood for antifilarial antibody (Wb123 assay) testing according to the suppliers instruction using ELISA. In total, a maximum of 3000 lymphedema cases are estimated to require this additional diagnostic test. Those lymphedema cases with negative antifilarial antibody assay will undergo further physical examination and history. In addition, information from physical examination including preservation of sensation in the toes, bilateral/unilateral, clinical signs of leprosy or onchocerciasis, and groin involvement will be recorded. History of rheumatic heart disease will also be asked.

GPS data:

Community GPS coordinates will be collected using Handheld Global Positioning System Geographical Positioning System (GPS-GarmineeTrex®).GPS coordinates will put in to a map using GIS software, to display the distribution of LF in Ethiopia.

Data collection and management

After the training, a pre-test of field procedures will be conducted. This research project will be managed by a team from EPHI, BSMS, AHRI and LSTM. An assigned person will be responsible for overall technical leadership, management and liaison, as well as the overall compliance of the project. The team will identify key personnel and form teams at zonal and regional level. Designated regional coordinators will be responsible for the regional level coordination and zonal coordinators will be responsible for locating

the identified clusters and making the necessary pre-survey arrangements including community sensitization and mobilization. The logistics aspect of the project will be coordinated by the field logistics coordinator: these include transport, laboratory tests, and other supplies. A training manual will be prepared by the team. Training on survey methods, interview techniques, the rapid diagnostic test for LF and the use of portable Geographical Positioning System (GPS-Garmin eTrex®) devices will be given to all members of the survey team. Initial training will be given in Addis Ababa with the aim of sensitizing regional leaders, explaining the objectives of the survey, and identifying and training potential survey team members. After the training, a pre-test of field procedures, including use of portable GPS devices, the LF rapid diagnostic test and podoconiosis disease staging, will be conducted. Based on the pre-test results, problems will be identified and corrected. In addition, the training will help the regional coordinators to select and make initial plans with the zonal coordinators. Following this, regional and zonal coordinators will organize training for data collectors in their respective areas. The standard training guide will be the source document for all training.

The actual field data collection date will be selected with input from regional and zonal coordinators, taking into account the heavy rainy season that can potentially hamper data collection and make some remote clusters inaccessible. The teams, consisting of the zonal coordinator, the interviewer and the village guide, will visit each cluster before the actual day of the survey to accomplish the following tasks: to inform local leaders and community representatives about the objectives of survey and get permission; to identify individuals to be included in the survey as per the protocol, to record geo-coordinates using a GPS device, to select a suitable day for the survey in consultation with the community members, and to record travel time and directions to the villages. The team will supervise data collection region by region, ensuring adherence to protocol and responding rapidly to problems encountered. There will be continuous communication with regional and zonal coordinators through mobile phones, so that problems arising during data collection are communicated for prompt action.

All forms completed at the field level will be received at the survey coordination office, manually checked for completeness and then systematically filed by village. Data from

the village forms, individual questionnaires, and LF examinations will be entered onto computer by experienced data entry clerks using Microsoft Access. Skip patterns and error checking will be integrated in the data entry forms. A regular backup system will be created in order to ensure no loss of data during the data entry process. By linking the village number (three digits), and individual code (English alphabet from A to P) a unique identification number for each individual in the survey will be established to facilitate data analysis. Data entry will be supervised routinely by an experienced data manager and regularly by the investigators.

The team will work closely with the Ministry of Health throughout the project, which will benefit from use of resources including vehicles and personnel from regional and zonal health offices. To establish smooth implementation of the research project, the national Neglected Tropical Disease lead at the Federal Ministry of Health will be involved during the planning and implementation of the survey.

Data entry and analysis:

Each completed data sheet will be checked by the field coordinator at field level. The collected data will be double entered in Access Data Base. The data manager will verify, clean the data and prepare the analysis plan with the PIs. Data verification, cleaning and analysis will be done using SPSS and STATA statistical Software and ARC- GIS software packages.

Ethical considerations:

Ethical approval need to be obtained from EPHI and national Ethical board. Appropriate support letters will be obtained from responsible administrative bodies and informed consent will be sought from participants. Confidentiality will be maintained using ID codes. Names of participants will not be recorded. Individual informed consent will be obtained from each participant (≥18 years of age). Additionally and for those 15 to 18 years old consent will be obtained from their parents/guardian (see attached document) and the participant themselves will provide informed assent (15-18 years old). Patient information sheet and assent and consent forms will be in Amharic languages, but as the

need arises, these forms will be translated to local language of the specific population and/or ethnic group. Forms are prepared in Amharic and English.

The confirmed *W. bancrofti* infection will be treated by co-administration of one tablet of albendazole 400mg and the required dose of ivermectin, as indicated by a dose-pole according to WHO recommendations. Furthermore if any positives are found in the village the entire district will be consider endemic and all eligible population will receive annual MDA for at least 5 years until LF is eliminated. The population will receive information on that.

Ethical approval was obtained from Liverpool School of Tropical Medicine for the LF mapping and from the School of Public Health, Addis Ababa, and the Research Governance & Ethics Committee of Brighton & Sussex Medical School (BSMS) for podoconiosis mapping.

As part of the LF and Podoconiosis elimination programs, Ethiopia will put in place a morbidity management and disability prevention plan that will include provision of care for the patients suffering from lymphoedema and hydrocele.

Expected output of the Study

1. Develop a distribution map of the LF and define endemic districts (≥1% positives found in at least one of the villages) and non-endemic districts. The percentage of positives will be estimated in each community as a proxy of level of endemicity in the district.
2. Develop a distribution map of Podoconiosis (the endemicity classification will be defined based on international consensus among experts in the field).
3. Give an estimate of population at risk for LF and Podoconiosis in Ethiopia.

Significance of the study

The results of the study will benefit the Federal Ministry of Health (MOH) and other stakeholders to develop an efficient and cost effective LF elimination and Podoconiosis

Control Programs by precisely locating endemic districts and affected and at risk communities.

Dissemination of Results and Ensuring the Implementation

The operational research outlined in the proposal will provide important data to provide key knowledge and fills the information gaps that are crucial for the development of LF elimination strategies and Podoconiosis control in Ethiopia. After completion of the analysis of the intended study, the results will be disseminated to all partners in a workshop that would be organized at national level. All inputs and comments from potential partners (Federal Mministry of Health, Regional Health Bureau and local communities) will be taken care of and the study results will be refined and distributed to all Stakeholders and international scientific communities through publications.

Monitoring/Evaluation of the studies and Action plan with Time.

The following Monitoring and Evaluation Plans are to measure the progress of the proposed activities. Deliverables and activities will be used as milestones to monitor and evaluate the study progress and achievements. See Annex I for the details of the activities)

TO CHANGE

S. No	Major Activities	Deliverables	Time frame	Responsible
1	Establish Partnership & Coordination	Finalize the study protocol with the participation of in-country stakeholders	Month 1	Partners
2		Micro-planning workshop will be conducted before kickoff		
3	Capacity Building	training workshop in LF and Podoconiosis mapping, survey implementation and study protocol will be conducted for project staff	Months 1	Partners
4	Implementation Research Activities	LF and Podoconiosis map project completion as per study protocol: collection of data and data entry into study database as well as analysis	Months 2-10	FMoH, RHB & participant investigators
5	Interim report and evaluation	Evaluation of project results and interim reporting	Months 11	Stakeholders and invited experts
6	report for Stakeholders	Submission of Draft report to stakeholder	Months 11	FMoH/Funding agency

7	Dissemination of LF and Podoconiosis Operational Research Results	Dissemination workshop (for stakeholders and publishing in peer-reviewed Journals.	Months 12-18	Partners

Research Team

Team composition:

- **From central level**

1 Nurse for a bleeding and clinical data recording.

1 Nurse for podoconiosis

1 Lab technician for reading ICT cards and record results.

1 Coordinator to organize the activity and collect the GPS coordinates and provide informed consent to the community

- **From local level**

2 local data recorder (data form, stick ID number)

1 local translator in some place

1 local community kebele leader for social mobilization

No	Professionals Required	Condition
1	Nurse	bleeding and clinical data recording
2	Nurse	Podoconiosis
3	Lab technician	reading ICT cards and record results
4	Coordinator	organize the activity and collect the GPS coordinates and provide informed consent to the community
4	Translator	
5	Data recorder	data form, stick ID number
6	Social mobilizer	

References

1. Molyneux DH (2012) Tropical lymphedemas--control and prevention. N Engl J Med 366: 1169-1171.
2. Addiss DG (2010) Global elimination of lymphatic filariasis: addressing the public health problem. PLoS Negl Trop Dis 4: e741.
3. Davey G, Tekola F, Newport MJ (2007) Podoconiosis: non-infectious geochemical elephantiasis. Trans R Soc Trop Med Hyg 101: 1175-1180.
4. Molyneux DH, Malecela MN (2011) Neglected tropical diseases and the millennium development goals: why the "other diseases" matter: reality versus rhetoric. Parasit Vectors 2011 Dec 13: 234.
5. Fan PC, Peng HW, Chen CC (1995) Follow-up investigations on clinical manifestations after filariasis eradication by diethylcarbamazine medicated common salt on Kinmen (Quemoy) Islands, Republic of China. J Trop Med Hyg 98: 461-464.
6. Zagaria N, Savioli L (2002) Elimination of lymphatic filariasis: a public-health challenge. Ann Trop Med Parasitol 96: S3-13.
7. Davey G (2009) Recent advances in podoconiosis. Ann Trop Med Parasitol 103: 377-382.
8. Tekola F, Mariam DH, Davey G (2006) Economic costs of endemic non-filarial elephantiasis in Wolaita Zone, Ethiopia. Trop Med Int Health 11: 1136-1144.
9. Davey G, Burridge E, (2009) Community-based control of a neglected tropical disease: the mossy foot treatment and prevention association. PLoS Negl Trop Dis 3: e424.
10. WHO (2010) Working to overcome the global impact of neglected tropical diseases: first WHO report on neglected tropical diseases. World Health Organization, Accessed on 25 July 2012. Available at [http//:whqlibdoc.who.int/publications/2010/9789241564090_eng.pdf].
11. Deribe K, Meribo K, Gebre T, Hailu A, Ali A, et al. (2012) The burden of Neglected Tropical Diseases in Ethiopia, and opportunities for integrated control and elimination. Parasit Vectors 5: 240.
12. Shiferaw W, Kebede T, Graves PM, Golasa L, Gebre T, et al. (2012) Lymphatic filariasis in western Ethiopia with special emphasis on prevalence of Wuchereria bancrofti antigenaemia in and around onchocerciasis endemic areas Trans R Soc Trop Med Hyg 106: 117-127.
13. Jemaneh L, Kebede D (1995) Clinico-epidemiological study of lymphatic filariasis southwestern Ethiopia. Ethiop Med J 33: 145-153.
14. Berhane Y, Hail Mariam D, Kloos H (2006) (eds). The Ecology of Health and disease in Ethiopia. Shama books , Addis Ababa. 1-308.
15. MORRONE A, TERRANOVA M, PADOVESE V (2007) Lymphatic filariasis in Tigray, Ethiopia. 21 world Congress of Dermatology Buenos Aires, 30 September - 5 October
16. Oomen AP (1969) Studies on elephantiasis of the legs in Ethiopia. . Trop Geogr Med 1969 3.

17. Price EW (1974) Endemic elephantiasis of the lower legs in Ethiopia an epidemiological survey. . Ethiop Med J 12: 77-90.
18. Mengistu G, Humber D, Ersumo M, Mamo T (1987) High prevalence of elephantiasis and cutaneous leishmaniasis in Ocholo, south-west Ethiopia. . Ethiopian Medical Journal 25: 203-207.
19. Kloos H, Kello AB, Addus A (1992) Podoconiosis (endemic non-filarial elephantiasis) in two resettlement schemes in western Ethiopia. . Tropical Doctor 22: 109-112.
20. Birrie H, Balcha F, Jemaneh L (1997) Elephantiasis in Pawe settlement area: podoconiosis or Bancroftian filariasis? . Ethiopian Medical Journal 35: 245-250.
21. Desta K, Ashine M, Davey G (2003) Prevalence of podoconiosis (endemic non-filarial elephantiasis) in Wolaitta, Southern Ethiopia. . Tropical Doctor 32: 217-220.
22. Alemu G, Tekola Ayele F, Daniel T, Ahrens C, Davey G (2011) Burden of podoconiosis in poor rural communities in Gulliso woreda, West Ethiopia. . PLoS Negl Trop Dis 5: e1184.
23. Oli GG, Tekola Ayele F, Petros B (2012) Parasitological, serological, and clinical evidence for high prevalence of podoconiosis (non-filarial elephantiasis) in Midakegn district, central Ethiopia. Trop Med Int Health.
24. Molla YB, Tomczyk S, Amberbir T, Tamiru A, Davey G (2012) Podoconiosis in East and west gojam zones, northern ethiopia. PLoS Negl Trop Dis 6: e1744.
25. Shiferaw W, Kebede T, Graves PM, Golasa L, Gebre T, et al. (2012) Lymphatic filariasis in western Ethiopia with special emphasis on prevalence of Wuchereria bancrofti antigenaemia in and around onchocerciasis endemic areas. Trans R Soc Trop Med Hyg 106: 117-127.
26. Deribe K, Brooker SJ, Pullan RL, Davey G, . (2012) Spatial distribution of Podoconiosis in Ethiopia: Results from historical maps and their implication on contemporary disease control. Unpublished data.
27. Brooker S, Utzinger J, (2007) Integrated disease mapping in a polyparasitic world. Geospat Health 1: 141-146.
28. Kabatereine NB, Standley CJ, Sousa-Figueiredo JC, Fleming FM, Stothard JR, et al. (2011) Integrated prevalence mapping of schistosomiasis, soil-transmitted helminthiasis and malaria in lakeside and island communities in Lake Victoria, Uganda. Parasit Vectors 13: 232.
29. Molyneux DH (2012) Tropical lymphedemas--control and prevention. N Engl J Med 366: 1169-1171.
30. Davey G (2010) Podoconiosis, non-filarial elephantiasis, and lymphology. Lymphology 43: 168-177.
31. Deribe K, Meribo K, Gebre T, Hailu A, Ali A, et al. (2012) The burden of Neglected Tropical Diseases in Ethiopia, and opportunities for integrated control and elimination. Parasit Vectors (in press).

Annex I: tentative time line and deliverables

**Activities**	2012		2013												2014		
	**Nov**	**Dec**	**Jan**	**Feb**	**Mar**	**Apr**	**May**	**Jun**	**Jul**	**Aug**	**Sep**	**Oct**	**Nov**	**Dec**	**Jan**	**Feb**	**Mar**
Identify stakeholders & partners that are either sources of data or technically important																	
Establish working group that involves in the project day to day activities & administrative links																	
Development of working protocol																	
Preparation of SOP for fieldwork																	
Procure supplies for mapping																	
Sign MOUs among partners																	
Identify data collectors and team leaders																	
Training of Trainers																	
Training of project staffs																	
Field data collection																	
Data entry																	
Development of geo-data base																	
Carryout spatial analysis																	
Producing LF Map																	
Interim report and evaluation																	
Consolidation of map																	
Final validation base on expert group comments																	
Dissemination of the final result to wider group																	
Drafting and submitting for publication																	

Annex 2- Information sheet
Title of Project: Mapping of Lymphatic Filariasis and Podoconiosis in Ethiopia

Lay Title: Mapping of Lymphatic Filariasis and Podoconiosis in Ethiopia
Name of Investigators:

Information Sheet

My name is …………………, and I am working with Addis Ababa University, BSMS,EPHI and AHRI. You are invited to take part in this research study, which we hope will yield valuable information on geographical distribution of elephantiasis. Before you decide whether to take part it is important for you to understand why we are collecting this information and what it will involve. Please take time to read this paper carefully and discuss it with friends and relatives if you wish to. Ask us if there is anything that is not clear or if you would like more information.

Background to the study.

We are mapping elephantiasis in Ethiopia. Through this study we identify the geographical distribution of the disease and environmental factors affecting the distribution. We hope that this will help us in scaling up prevention and treatment of elephantiasis throughout the country. With your permission, we intend to:

1. Ask you a series of questions about you and your family, the way you live and work, and in particular, the contact you have with the red soil. If you have elephantiasis, we will also ask questions related to the disease and how you have managed it.

2. We would like to take a sample of blood. For this we prick your finger lightly with a fine needle and take a drop of blood. The blood sample will help to see if either of you have LF or not. The samples will be analysed here onsite. We will not test for any other diseases with this blood sample.

3. In areas in which LF is discovered, if you have elephantiasis, we would like to double check the fingerprick test by taking a second sample of blood into a small tube. The amount of blood is small (about 5ml or what is held on a small teaspoon). We will store this so we can do further checks to distinguish the type of elephantiasis you have." And "If I have elephantiasis, I understand that the investigators will take samples of blood to distinguish which elephantiasis it is.

Possible harms. We do not anticipate any harm to you from asking the questions or collecting the blood samples. The questions will take a maximum of 30 minutes of your time.

Benefits. At the end of the questions, we will explain to you more about the condition and how to prevent and treat it. If appropriate, we will put you in touch with a treatment site if there is one nearby.

Confidentiality. All information which is collected about you during the course of the research will be kept on a password protected database and is strictly confidential. Any information about you which leaves the research unit will have your name and address removed so that you cannot be recognized from it.

Autonomy. If you wish to discontinue the questionnaire or the sample collection at any time, you may, however, all the information you give us is highly valuable to the study. If you decide to take part you will be given this information sheet to keep and be asked to sign a consent form. If you decide to take part you are still free to withdraw at any time and without giving a reason. If you decide not to participate, the treatment you or your family receives in future at government or NGO treatment sites will not be affected.

If something goes wrong. If a problem arises, you can report it to one of the project staff, your *kebele* head, or the study coordinators at the address given below.

What will happen to the research? We anticipate that the results of this immediate study will be available next year, and we hope to publish the results. You will not be identifiable in any publication.

Who is organizing and funding the research? The research has been funded by the Wellcome Trust and DFID, a UK-based funding bodies dedicated to improving human and animal health through research. The research is organized jointly by researchers in Addis Ababa and the UK. The research has been reviewed by the Institutional Review Board of the Faculty of Medicine, Addis Ababa University and the National Ethical Review Committee in Ethiopia, and by Brighton & Sussex Medical School Ethics Committee and Liverpool School of Tropical Medicine.

Contact Address:__________________ or __________________

Cell Phone: ____________________ __________________

Thank you in advance for considering taking part in this additional study!

የመረጃ ቅጽ

የኘሮጀክቱ አርዕስት

በ ኢትዮጵያ የዝሆኔ በሽታን ስርጭት በካርታ ለማሳየት የሚደረግ የዳስሳ ጥናት

መ□በኛ ርዕስ: ***የዝሆኔ በሽታን ስርጭት ካርታ***

የአጥኝዎች ስም፡-

የመረጃ ቅጽ

ስሜ -------------------- ሲሆን የምሰራውም ከአዲስ አበባ ዩኒቨርሲቲ ጋር ነው። በዚህ ጥናት የዝሆኔ በሽታን ስርጭትና ካርታን ይነደፋል። ይህ ጥናት ጠቃሚ መርጃ ይሰበስባል ተብሎ ይታመናል። በዚህ ጥናት እን□ሳተ□ ተጋብዘዋል።ለመሳተፍ ከመወሰኖ በፊት ጥናቱ ለምን እንዳስፈለገና ምንምን እንደሚያጠቃልል ማዋቅ ይኖርቦታል። እባክዎን ጊዜ ወስደው የሚከተለውን መግለጫ በጥንቃቄ ያንብቡት ካስፈለጉትም ከጋደኞችዎና ከዘመዶችዎ ጋር ይወያዩበት:: ግልጽ ያልሆነ ነገር ካለና ተጨማሪ ማብራሪያ ከፈለጉ ሊጠይቁን ይችላሉ።ተሳትፎዎን ለመወሰሰን ጊዜ ይውሰዱ።

የጥናቱ መሰረት

እኛ አሁን የምናጠናው የዝሆኔ በሽታን ስርጭትና ካርታን መንደፍ ነው። በተጨማሪም የ በሽታውን ስርጭት የሚወስኑ አከባቢያዊ መክንያቶችን ያጠናል። የዚህም ጥናት ውጤት የዝሆኔ በሽታን ለማከላከልና ለማከም በሚደረገው ጥረት ጉልህ ድርሻ ይኖረዋል። በእርሰዎ ፈቃድ አሁን

1. *ስለ እርሰዎ እና ስለ ቤተሰብዎ ቃለ መጠይቅ እንጠይቃለን። የዝሆኔ በሽታ ካለብዎ ተጨማሪ ጥያቄዎችን እንጠይቆታለን።*
2. *ስለዚህም የእርስዎን ደም ናሙና ለመውሰድ እንፈልጋለን።ናሙናው የሚወሰደው በትንሽ ፖላስቲክ መያዥ ሲሆን መጠኑም በጣም አነስተኛ ነው። አላማውም እርሶ የዝሆኔ በሽታ እንዳቦትና እንደሌሉበት ለማወቅ ነው። ናሙናው እዚሁ ምርመራ ይካሄድበታል።*
3. *ሊንፋቲክ ፊላሪያሲስ በሚገኝበት አካባቢ የእግር እብጠት ካለብዎት ተጨማሪ የደም ምርመራ በማድረግ ማረጋገጥ እንፈልጋለን። ስለዚህም ተጨማሪ የደም ምርመራ* 5 *ሚሊ ሊትር የደም ናሙና በትንሽ ብልቃጥ እንወስዳለን።የተወሰደውም የደም ናሙና በ ላቦራቶሪ ይቀመጣል። የደም ናሙናው የትኛው የዝሆኔ በሽታ እንዳለብዎት ለማረጋገጥ ለሚደረግ ምርመራ ብቻ እንጠቀምበታለን። የደም ናሙናው ለአገልግሎቱ ከዋለ በሁዋላ ለሌላ አገልገሎት አይውልም።*

ሊከሰቱ የሚችሉ ጉዳቶች፡- *የደም ናሙና በመውሰድ የሚደርስ ምንም አይነት ጉዳት አይኖርም።ይህ ቃለመጠይቅ ከ* 30 *ደቂቃ የማይበልጥ ጊዜ ይወስዳል።*

ጥቅሞች፡- *በጥናቱ መጨረሻ ስለ በሽታው መተላለፊያና መከላከያ ትምህርት ይሰጥዎታል። በሽታው ከለብዎ እና በ አቅራቢያ የህክምና ጣቢያ ካለ ወደ ጣቢያው እንመርዎታለን።*

የሚስጥር አጠባበቅ:*ማንኛውም ስለእርስዎ በጥናቱ የሚሠበሰብ መረጃ ሚስጥርነቱ በተጠበቀ መልኩ ይቀመጣል። ማንኛውም እርስዎን የሚመለከት መረጃ ከጥናት ክፍል ሲወጣ ስምን አድራሻዎ እንዳይኖረው ይደረጋል።*

በራስ መወሰን፡ በማንኛውም ጊዜ ቃለመጠይቅ ስለማድረግ የደም ናሙና ላለመስጠት ከፈለጉ ማቋረጥ ይችላሉ ነገር ግን የሚሠጡን መረጃ ለጥናቱ በጣም ጠቃሚ መሆኑን አይዘንጉ በጥናቱ ስለመሣትፍና ስለመሣትፎ ወሣኝ እርስዎ ኖት ለመሳተፍ ከወሱኑ ይህንን የመተማመኛ ቅጽ እንዲፈርሙ ይጠየቃሉ።አሁን ለማዳተፍ ፍቃደኛ ቢሆኑም ስለማስተፉ ቢወስኑ እርስዎ ሆነ ቤተሰብዎ ከ ጤና ጣቢያና ሆስፖታል እንዲሁም መንግስታዊ ካለሆኑ ድርጅቶች በሚወስዱት አገልግሎት ላይ ምንም አይነት ተጽእኖ አይኖርም።

አንደንድ ችግሮች ቢያጋጥሙ: ችግር ቢያጋጥም ለኘሮጀክቱ ሰራተኞች ለቀበሌዎ ኃላፊዎች ወይንም ለጥናቱ አስተባባሩ ከዚህ በታች በተፃፈው አድራሻ ሪፖርት ማድረግ ይተላሉ።

የጥናቱ መጨረሻ ምንድነው? የዚህ የጥናት ውጤት በሚመጣው አመት እንደሚታወቅ እንገምታለን:: ስለዚህም ውጤቱን ለማሰማት እንጥራለን። በሚታተምበት ወቅት እርሶን መለየት አይቻልም።

ማንነው ጥናቱን የሚያስተባብረው እና በገንዘብ የሚደገፈው? ይሄ ጥናት ዌልካም ትረስት እና ዲፍአይዲ በሚባል እንግሊዝ ሃገር በሚገኙ የበጐ አድራጐት ጅርጅቶች በገንዘብ የሚደገፍ ሲሆን የ ድርጅቶቹ ዓላማ የሰዎችንና የእንስሳቶችን ጤንነት በምርምር እና በጥናት ማሻሻል ነው። ይህ ጥናት አዲስ አበባና እንግሊዝ ሀገር በሚገኙ አጥኚዎች ትብብር የሚሠራ ነው። ጥናቱ በ አዲስ አበባ ዩንቨርሲቲ የህክምና ትምህርት ቤት የስነምርምር አርታኢ በሀገር አቀፍ የስነምርምር አርታኢ ኮሚቴ በኢትዮጵያና በ ብራይተን እና ሰሴክስ ዩንቨርሲቲ የህክምና ትምህርት ቤት የስነምርምር አርታኢ ኮሚቴ እና በሊቨርፑል ስኩል ኦፍ ትሮፒካል ሜዲስን ታይቷል።

በዚህ ጥናት ላይ ለመሳተፍ ፈቃደኛ በመሆንዎ አስቀድሜ ላመሰግኖት እፈልጋለሁ።

Annex 3- Healthy volunteer consent form

Reference ID Number | | | | |

Title of Project: **Mapping of Lymphatic Filariasis and Podoconiosis in Ethiopia**
Name of Investigators:

Healthy Volunteer's Consent Form

Please read this form and sign it once the above named or their designated representative, has explained fully the aims and procedures of the study to you

- I voluntarily agree to take part in this study.
- I confirm that I have been given a full explanation by the above named and that I have read and understand the information sheet given to me which is attached.
- I understand that the investigators will ask a series of questions about me and my work.
- I understand that the investigators will take samples of blood to test for LF.
- I have been given the opportunity to ask questions and discuss the study with one of the above investigators or their deputies on all aspects of the study and have understood the advice and information given as a result.
- I agree to comply with the reasonable instructions of the supervising investigator and will notify him immediately of any unexpected unusual symptoms or deterioration of health.
- I authorize the investigators to disclose the results of my participation in the study but not my name.
- I understand that information about me recorded during the study will be kept in a secure database. If data is transferred to others it will be made anonymous. Data will be kept for 7 years after the results of this study have been published.
- I understand that I can ask for further instructions or explanations at any time.
- I understand that I am free to withdraw from the study at any time, without having to give a reason.
- I confirm that I have disclosed relevant medical information before the study.
- I understand that if something goes wrong I can report it to one of the project staff or the study coordinators at the address given below.

Name: ……………………………………………………………………………………

Address: …………………………………………………………………………………..

Telephone number: ……………………………………………………………………….

Signature: …………………………………………. **Date:** ……………………………….

I confirm that I have fully explained the purpose of the study and what is involved to:

……………………………………………………………………………………………….

I have given the above named a copy of this form together with the information sheet.

Investigators Signature: ……………………….. **Name**: ………………………………

<u>የጤነኛ ፈቃደኞች የስምምነት ቅጽ</u>

የኘሮጀክቱ አርዕስት

<u>በ ኢትዮጵያ የዝሆኔ በሽታን ስርጭት በካርታ ለማሳየት የሚደረግ የዳሰሳ ጥናት</u>

መ□በኛ ርዕስ: **የዝሆኔ በሽታን ስርጭት ካርታ**

የአጥኚዎች ስም፡-

<u>የጤነኛ ፈቃደኞች የስምምነት ቅጽ</u>

ከላይ ከተጠቀሰው ሰዎች መካከል ወይም የእነርሱ ተዋካይ የጥናቱን አላማና ሂደት በዝርዝር ካስረዳዎት በኃላ የሚከተለውን ቅጽ በጥንቃቄ ይፈርማሉ፡፡

- ❖ በሙሉ ፈቃድ የጥናቱ ለመሳተፍ ወስኛለሁ፡፡
- ❖ ከዚህ ጋር የተያያዘውን የመግለጫ ቅጽ በትክክል አንብቤ ተረድቻለሁ፡፡ በተጨማሪም አስፈላጊውን ገለፃና ማብራሪያ ከላይ በተጠቀሰው ሰው ተደርጐልኛል፡፡
- ❖ ጥያቄ የመጠየቅ መብት የመወያየት እድል ከላይ ከተጠቀሱት አጥኚዎች ወይዋ ከነሱ ተወካይ ጋር ተሰጥቶኝ በጥናቱ ላይ በቂ ምክርና ውይይት አድርጊያለሁ፡፡
- ❖ በአጥኚው ሱፐርቫይዘር የተሰጠኝን መመሪያና ምክር ለመቀበል ዝግጁነኝ፡፡ ማንግውምንም ያልተጠበቁ ውጤት የበሽታ ምልክትና የጤና ችግር በቶሎ ለሱ ለመናገር ዝግጅ ነኝ፡፡
- ❖ በተመራማሪዎቹ የጥናቱን ውጤት ይፋ እንዲያደርጉ እፈቅዳለሁ፡፡ ነገር ግን ስሜ መጠቀስ የለበትም፡፡
- ❖ ተመራማሪዎች በጤናዬ ላይ ያለን ችግር እንዲነግሩኝ ፈቅጃላቸዋለሁ /ይህ የማይሰራ ከሆነ ይሰረዝ/
- ❖ በማንኛውም ጊዜ ተጨማሪ ማብራሪያ ገለጻዎችን መጠየቅ እንደምችል አውቂያለሁ፡፡
- ❖ በማንኛውም ጊዜ ያለምክንያት ከጥናቱ ራሴን ማግለል እንደምችል አውቂያአለሁ፡፡
- ❖ ስለ እኔ እና ስለ ቤተሰቤ ቃለ መጠይቅ እጠየቃለሁ፡፡
- ❖ የደም ናሙና ለዝሆኔ በሽታ ምርመራ እንደሚወሰድ ተነግሮኛል፡፡
- ❖ በዚህ ጥናት እኔን በሚመለከት የሚወሰዱ መረጃዎች በተጠበቀ የመረጃ ማዕከል ለ 7 ዐመታት ይቀመጣል፡፡ የህ መረጃ ለሌላ ወገን ከተላለፈ የእኔ መንነት አይገለጸም፡፡
- ❖ በጥናቱ ሂደት ያልተጠበቀ ነገር ቢከሰት ለፕሮጀክቱ አባላት ወይንም ለጥናቱ አስተባባሪቆች ከታች በተጠቀሰው አድራሻ አሳውቃለሁ፡፡
- ❖ ከጥናቱ በፊት ስለጤናዬ በቂ መረጃ መስጠቴን አረጋግጣለሁ፡፡

ሥም -- አድራሻ ---------------------------------

የስልክ ቁጥር ------------------------------ ፊርማ ------------------------ ቀን ---------------------

የጥናቱ አላማና በውስጡ የተጠቃለሉ ሂደቶች ሁሉ በዝርዝርና በግልጽ ተነግረውኛል፡፡

ከላይ ስማቸው ለተጠቀሰው ግለሰብ የዚህን ቅጽ ኮፒና የመረጃ ቅጽ ሰጥቻቸዋለሁ፡፡

የተመራማሪው ሥም --------------------------- ፊርማ ---------------------

Annex 4- Questionnaire

Podoconiosis mapping study questionnaire

Date	(DD-MMM-YYYY)	|___|___|-|___|___|___|-|___|___|___|___|	Record Taker's Initials	|___|___|___|
Name of Community			Community Code	|___|___|___|
Participant Identification number		|___|___|-|___|___|___|-|___|___|___|		

Section I Demographic and Socioeconomic Information			
**SN**	**Questions and Filters**	**Response & Coding Categories**	**Skip**
101	Region name		
102	Zone Name		
103	Woreda code		
104	Kebele Code		
105	Community Code		
106	Sex Check box (✓)	☐ 1 = Male ☐ 2 = Female	
107	How old are you?(years)	>15 years of age	
108	Religion	☐ 1 = Muslim☐ 2 = Christian ☐ 3 = Animist ☐ 4 = Other	
109	Ethnic group	_______________	
110	Where is your permanent residence?	☐ 1 = Rural ☐ 2 = Urban	
111	How long you lived in the current location?		
112	What is your major occupation currently?<br>(Whatever you do to earn money)?	☐ 1 = Employed<br>☐ 2 = Businessman/women<br>☐ 3 = Farmer<br>☐ 4 = Housewife<br>☐ 5 = Daily laborer<br>☐ 6 = Student<br>☐ 7 = Have no Job<br>☐9 = Other specify ____________	
113	Are you able to read and write in any language?	☐ 1 = Yes ☐ 2 = No<br>Grade completed ________	
114	What is the monthly income (on average) of your household including your own?	Birr/month____________________	
115	What type of floor does your house have?	☐1=Mud/earth ☐2=Wood ☐3=Cement<br>☐9=Other	
116	What is your current marital status?<br>Check box (✓)	☐1=Single ☐2= Married<br>☐3 = Divorced ☐4 = Widowed	
**Section II Shoe wearing and foot care practice**			
201	Have you ever worn shoes?	☐ 1 = Yes<br>☐ 2 = No →	Go to Q206
202	How old were you when you first got shoes?		
203	Is the person wearing shoes at the time of the interview?	☐ 1 = Yes<br>☐ 2 = No→	Go to Q205
204	Describe the shoes the person is wearing.	☐1= Hard plastic ☐2= Open sandal<br>☐ 3=Leather ☐ 4=Shera ☐ 5=other	
205	When do you wear shoes?(multiple answers possible)	☐1= At home<br>☐ 2=During rainy season<br>☐3= On market days	

		☐4= On the field<br>☐5= On Sundays<br>☐ 6=When walking far	
206	How long (in min) does it take you to go to the nearest water source?		
207	When do you wash your feet?	☐1=Whenever they are dirty<br>☐2=Before sleeping<br>☐3=Before prayer<br>☐9=Other (specify)________	
208	How often do you wash your feet very carefully so that they are very clean?	☐1= More often than once a day<br>☐2= Daily<br>☐ 3=Less often than daily, but more often than weekly<br>☐4= Weekly or less often	
**Section III Leg swelling history ICT Card Results and physical examination**			
301	Do you have leg swelling?	☐ 0 = No ⟶<br>☐ 1= Yes (verify by observation)	End the interview
302	Do you have any family member (living or dead) with history of leg swelling?	☐ 0 = No ⟶<br>☐ 1 = Yes	Go to Q 304
303	How many people in your family (living or dead) have leg swelling?		
304	How old were you when you first noticed this swollen leg?		
305	Where did the swelling start from?	☐1= from high up<br>☐2= From the foot or lower leg	
306	Do you have history of rheumatic heart disease?	☐ 0 = No ☐ 1 = Yes	
307	Do you have swelling in the groin area?	☐ 0 = No ☐ 1 = Yes	
308	Are you diagnosed as a leprosy patient?	☐ 0 = No ☐ 1 = Yes	
309	Is there preservation of sensation in the toes? (Physical examination)	☐ 0 = No ☐ 1 = Yes	
310	Podoconiosis diagnosis established	☐ 0 = No ☐ 1 = Yes	
311	Podoconiosis disease stage	☐ 1 = Stage 1 ☐ 2 = Stage 2<br>☐ 3 = Stage 3 ☐ 4 = Stage 4<br>☐ 5 = Stage 5	
312	Antifilarial antibody test blood sample collected?	☐ 0 = No<br>☐ 1 = Yes	

The end! Thank you for giving us your time and answers to many questions. We hope this will help the work in the future.

The end!

Thank you for giving us your time and answers to many questions. We hope this will help the work in the future.

Questionnaire

Code No: ………………………….

Date: ……….. / ……….. /…………

CRF for Lymphatic Filariasis Mapping Survey

Identification & Demographic Data

Administrative Region:……………… Zone ……………..…………District……………………………

Kebele: ………………………………Village:……………………………………………………………

Name:…………………………… Age:…… ….Sex:

Marital Status:……………………………Occupation:……………………………Religion:……………………………

Marital Status:………………………Occupation:………………… ……. Religion:……………………………….

Ethnic Group:………………… Place of Birth:……………Duration of Stay at Residence:………………………

History

Major Complaints:………………………………………………………………………………………

Describe:………………………………………………………………………………………………..

Previous History of Filariasis Tx:……………………………………………………………………

Previous History of Onchocerciasis Tx :…………………………………………………………….

Physical Signs/Symptoms

Pain: Yes ☐ No ☐ Site:…………………………….. Note:…………………………………………

Swelling: Yes ☐ No ☐ Site:………………………………Note:………………………………………….

Redness: Yes ☐ No ☐ Site:……………………………… Note:……………………………………………

Abscess: Yes ☐ No ☐ Site:……………………………… Note:…………………………………………..

Fever: Yes ☐ No ☐ Note:…………………………………………………………………………….

Nausea: Yes ☐ No ☐ Note:……………………………………………………………………………

Vomiting: Yes ☐ No ☐ Note:…………………………………………………………….……..

Lower Limbs: Yes ☐ No ☐ Note:…………………………………………………………

Breasts: Yes ☐ No ☐ Note:………………………………………………………….

Scrotum: Yes ☐ No ☐ Note:…………………………………………………………

Penis: Yes ☐ No ☐ Note:…………………………………………………………

Vulva: Yes ☐ No ☐ Note:………………………………………………………….

Chyluria: Yes ☐ No ☐ Note:…………………………………………………………….

Heamaturia: Yes ☐ No ☐ Note:………………………………………………………….

Others (If any): ……………………………………………………………………………….

Bed Net Utilization: Yes ☐ No ☐ Note:…………………………………………………………

Laboratory Findings

ICT skin test:..…………………………………………………………………………………….

Microfilarial Density: …………………………………………………………………………………….

Other blood parasites:…………………………………………………………………………………….

Intestinal parasites:…………………………………………………………………………………….

Other tests (if any):……………………………………………………………………………………

Examined by:…………………………………….Signature:……………………………Date: …..…………..

Annex 5.

Mapping for Lymphatic filariasis in Ethiopia

This is a practical training course for the Ethiopian Federal Ministry of Health (FMOH) field teams conducting mapping surveys for lymphatic filariasis (LF) and podoconiosis in Ethiopia.
The course will cover the following topics:

1. Introduction to Lymphatic Filariasis
2. Purpose of Mapping Survey
3. Guidelines for Field Mapping Activities
4. Standard Operating Procedure for ICT cards
5. Data Collection Using Mobile Phones
6. Checklist for Conducting Survey
7. Frequently Asked Questions
8. Appendices

At the end of the course you should be equipped with all the information required to conduct mapping for the above mentioned diseases.

1. Introduction to Lymphatic Filariasis

Commonly known as elephantiasis, LF is a painful disease, which can cause profound disfigurement and is classified as a neglected tropical disease (NTD). The infection is usually acquired during childhood but visible manifestations do not appear till later on in life and can cause temporary or permanent disability. The disease is caused by a thread like worms known as filariae – *Wuchereria bancrofti.* These worms form “nests” within the human lymphatic system and can cause a variety of clinical manifestations including lymphedema of the limbs, genital disease (hydrocele, chlyocele and swelling of the penis and scrotum).

The disease is transmitted when mosquitoes with the infective stage larvae take a blood meal from a human host. The parasites enter the body through the skin, migrate to the lymphatic vessels and develop into adult worms, causing damage and dilation to the lymphatic vessels. The adult stage of the parasite lives inside the human host for several years producing millions of immature microfilariae (mf) that circulate in the peripheral blood vessels.These mf are ingested by mosquitoes that bite the infected human and after further development inside the mosquito are passed again onto another human.

The occurrence of LF in Ethiopia was first documented in 1971. The FMOH in collaboration with Addis Ababa University, Carter Centre-Ethiopia, and the World Health Organisation (WHO)-Ethiopia initiated the mapping of LF in the country in the following five regions: Benishangul-Gumuz, Gambella, SNNPR (Keffa, Sheka, and Benchmaji zones), Oromia (i.e. West Wollega, zone), and Amhara (i.e. North Gondar zone). The results of this survey indicated that LF is endemic in 34 districts of the 5 regions. Furthermore, a few pocket areas were identified that needed to be surveyed.

2. Purpose of mapping Survey

The FMoH is looking to eliminate LF from Ethiopia. In order to initiate the elimination programme, the first step is to identify areas where active transmission of LF is occurring. This is assessed by mapping the disease distribution using quick and easy diagnostic tools and identifying physical signs and symptoms. Once this information has been collected, the second step is to deliver treatment to all eligible individuals living in areas endemic for the disease,

through annual mass drug administration (MDA). Treatment is free and will consist of two drugs: albendazole and ivermectin. The drugs kill circulating mf within humans infected with the disease and will be distributed every year until transmission has been interrupted LF is eliminated from Ethiopia.

3. Guidelines for field mapping activities

Mapping will include collection of a variety of information including; community information, socio-demographic data, signs and symptoms caused by LF, detection of the parasite and utilisation of bednets.

Equipment

The mapping surveys require minimal equipment and aim to be rapid. Each mapping team will be provided with the following items for field surveys:

- Immuno-chromatographic test(ICT) cards
- Single Use Sterile Disposable Safety Lancets
- Alcohol
- Cotton
- Gloves
- Hand sanitizers
- Soap
- Bleach
- Insecticide
- Safety Box
- Plastic waste bags
- Plastic sheet
- Stationary
- Mobile Phones for Data Collection
- Informed Consent Forms

N.B. Item quantities may vary dependent on the number of sites the team will survey

Method

In each district, the mapping survey will be conducted in two sites that are selected by the **FIELD TEAM** based on the district/Woreda health data and reports on hydroceles and lymphodemas. In **EACH OF THESE SELECTED SITES,** information must be collected for a total of **100 RANDOMLY SELECTED INDIVIDUALS,** aged 15 years or above.

For true representation of the disease within the community and ethical reasons, not all individuals are eligible to participate in the mapping survey and it is important to adhere to the exclusion criteria below:

- Does not meet the minimum age requirements
- Has not lived in the community for a minimum of 10 years (as the individual may not be representative of the local population)
- Has not provided consent
- Is severely sick

To collect the data, the field teams will be given mobile phones with a set of questions that the data collection members (Team Leader and Nurses) will have to enter information on.

Community and Demographic Information

COMMUNITY INFORMATION: This should be collected prior to the start of the field surveys by the Team Leader. This will collect basic information of the community in the following areas; such as the GPS location of the site, community name, population size etc. The unique coding and ID system used in these forms will be pre-set and provided to the Team Leader by FMOH.

DEMOGRAPHIC INFORMATION: This should be collected during the field survey by the LF nurses. Each individual participating in the survey will be asked a number of questions about themselves such as gender, occupation, bed net usage, physical signs or symptoms of LF.

Please refer to the Data Collection Using Mobile Phones section for more information regarding all data collection and entry.

Diagnostics - ICT Cards

Presence of the LF parasite within individuals will be determined by using the ICT card, which is a rapid diagnostic tool, used commonly in LF elimination programmes. The test will be conducted by the Laboratory Technician within the field team and the results entered into the phone by the Nurses.

It is essential for the Laboratory Technician to follow general health and safety practices as they will be dealing with biological material which could potentially be hazardous. Gloves must be worn at all times and all laboratory supplies used for each test must be discarded safely. New swabs and fresh alcohol must be used to clean the skin before using a lancet to puncture the skin. The lancets are single use only and therefore must be discarded into the safety boxes after use. Once the results have been recorded, ICT cards should be disposed of in waste bags and incinerated.

Please refer to the Standard Operating Procedure for ICT section for further details on how to conduct the diagnostic test.

Ethical Considerations

- Participation in the survey is **VOLUNTARY** and it is imperative that nobody within the community feels forced to take part or concerned that not participating may affect any future health benefits they receive.
- Selection of individuals to take part in the survey must be **RANDOM** and there should be no bias in selecting one individual over another.
- To ensure **ANONYMITY**, individuals will be identified by a unique identity number only and personal details such as name or address must not be requested during the mapping process.
- It is important to get written approval from the community leaders prior to starting the field surveys. This will confirm that they are informed about the survey happy for it to be conducted.
- All participants will be requested to provide **WRITTEN CONSENT** that they understand the purpose of the survey and voluntarily are happy to take part in the survey. Individuals who are under the age of 18 years, they will require additional consent from their parents or legal guardian.
- Individuals who are positive should be provided **TREATMENT**. Treatment will not be administered as part of the mapping survey and participants,who are tested positive, will be advised to visit their local health facility, where they will receive medications for free. The national programme will also distribute drugs through the MDA strategy shortly after the mapping results have been analysed.

Additional Notes:

(Write below any additional notes from the facilitator)

………

………

………

………

………

………

………

………

………

………

………

………

………

………
………
………
………
………
………
………
…………………………………………………………

Field Teams

As an integral member of a small field team for conducting the mapping surveys, team work and coordination is essential. Field teams will be composed of individuals with a variety of skill sets each of who have specific responsibilities as outlined below:

**Team Member**	**Quantity**	**Key Responsibilities for LF Mapping**<br>***(Key responsibilities for podoconiosis will be outlined in the podoconiosis training)***
Team Leader	1	Community<br>• Communicate with community leaders to obtain support and consent for mapping<br>• Communicate with community members to answer questions regarding LF, mapping and the LF elimination programme<br>• Point of contact for community during mapping<br>Field Team<br>• Lead the field team to conduct mapping surveys<br>• Oversee surveys and supervise field team activities, ensuring all team members are following the mapping protocol<br>• Point of contact for EPHI/FMOH<br>• Monitor all field data collection<br>• Manage time of field activities and time spent on each mapping site<br>Data Collection<br>• Collect GPS points for mapping site<br>• Record LF community form information<br>• Responsible for safe keeping of mobile phones and data at all times<br>• Inform participant of ICT result and record data into data collection form for result<br>Other<br>• Advise on treatment and answer further questions regarding ICT result<br>• Have knowledge on how to conduct all aspects of the mapping survey, including the ICT card<br>• Activities associated with mapping e.g. assisting other field team members<br>• Ensure local health facilities have drugs available for treatment referral or

Team Member	Quantity	Key Responsibilities for LF Mapping<br>*(Key responsibilities for podoconiosis will be outlined in the podoconiosis training)*
		inform where FMOH where treatment cannot be provided
Nurse	2	Communication<br>• Communicating with members of the community regarding LF, mapping and the LF elimination programme<br>• Conducting physical examination of individuals participating in the mapping<br>Data Collection<br>• Enter all information accurately into data collection systems using mobile phones<br>Other<br>• Activities associated with mapping e.g. assisting other field team members<br>*(One nurse will focus on conducting the LF mapping, whist the other will focus on podoconiosis mapping.)*
Laboratory Technician	1	Data Collection<br>• Prepare field lab for conducting LF diagnosis (ICT card)<br>• Collect blood sample from participant<br>• Ensuring correct allocation of ID number for participant and test<br>• Conduct ICT card test and determine ICT result<br>• Time management so ICT cards are read within the required time according to ICT card instructions<br>• Deliver ICT card results to LF Nurse and ensure capture correctly in data collection form<br>• Ensure good health and safety practices are followed for sample collection and disposal of all ICT card test material.<br>• Inform participant how the test will be conducted, what samples will be taken and what the result will show.<br>• Answer any questions associated with LF, mapping, the LF elimination programme and the ICT card test<br>Other<br>• Activities associated with mapping e.g. assisting other field team members
Translator	1	Communication<br>• Sensitise and inform the local community about LF, the mapping survey and the FMOH LF elimination programme<br>• Translate communication by mapping team member<br>• Translate communication by participant<br>• Ensure communication is sensitive to local cultural and behavioural settings<br>Other<br>• Activities associated with mapping e.g. assisting other field team members

Team Member	Quantity	Key Responsibilities for LF Mapping<br>*(Key responsibilities for podoconiosis will be outlined in the podoconiosis training)*
Field Worker (Local)	2	Communication<br>• Obtain written consent from participant<br>• Sensitise and inform the local community about LF, the mapping survey and the FMOH LF elimination programme<br>• Motivate community members to participate in the survey<br>• Allocate identification (ID) number for participant<br>• Time slot allocation to ensure participants are not waiting for long periods of time unnecessarily<br>Other<br>• Activities associated with mapping e.g. assisting other field team members
Driver	1	• Safely transport all field team members and equipment from between mapping sites<br>Other<br>• Activities associated with mapping e.g. assisting other field team members

On the day activities

Generally it is expected that mapping surveys for one site should take no longer than 1.5 days. On day 1, it is anticipated that there will be no field work and main activities will include sensitising community leaders and obtaining written approval that the survey can take place - this should be half a day's work. Field work should usually be conducted on day 2; participant recruitment, data collection and diagnostic testing.

AT EACH NEW DISTRICT/WOREDA, the field team will need to visit the district/Woreda level health centre first to obtain health records and review lymphedema and hydrocele information; to identify **TWO** suitable sites for conducting the mapping survey.

Day 1

Activities require: **Team Leader, Translator, Field Workers** and **Driver**

1. Team members travel to site within district
2. Meet with Community Leaders and introduce team
3. Team leader to discuss with community leaders the following areas;
    a. **LF:** What LF is and how it is transmitted?
    b. **FMOH Plans:** Initiation of national LF elimination programme
    c. **Mapping LF Distribution:** Aim and purpose of mapping, methodology, information collected, diagnostics used, benefits from participation, ethics and time required to conduct mapping
    d. **Treatment for LF:** If individuals are positive what they should do? What treatment should they seek?

   e. **Result of LF Mapping:** What will happen next? Analysis of results, findings translated into treatment strategy for national elimination programme
   f. **Consent to Map:** Discuss conducting the mapping survey in their community and obtain written consent that the mapping survey can be conducted there
   g. **Location for Mapping:** Discuss where the mapping survey can be conducted (central meeting point)
   h. **Community Involvement:** Request and motivate leaders to conduct **Community Sensitisation And Social Mobilisation**; to inform their community to participate in the mapping survey and requirements from local health facilities
   i. **Time:** Discuss time schedule of mapping survey
   j. **Other:** Any further site specific requirements
4. Team to identify location for conducting the mapping with Community Leader
5. Team Leader to collect Community information on mobile
6. Team to inform local health facilities of mapping survey
   a. If no drugs are available the Team Leader should contact FMOH and inform them that treatment is required for individuals who are positive in the specific mapping site.
7. Put up sensitisation material in places where community will see to inform them of upcoming survey (e.g. schools, local health facilities and community meeting place)
8. Return to rest of field team and prepare for mapping survey the next day

Additional Notes:

(Write below any additional notes from the facilitator)

……

……

……

……

……

……

……

……

……

……

…………………………

Day 2

Activities require **ALL MEMBERS OF THE FIELD TEAM**

1. Team members travel to site within district
2. Team meet with community leader and introduce team
3. Team members go to selected location and set up field mapping laboratory
   a. **Laboratory Technician** to set up LF diagnosis station

   b. **Nurses** to set up for data collection stations for LF and podoconiosis
4. **Community Leaders** assisted by local **Field Workers** round up community members to mapping laboratory
   a. **Community Leaders** must lead the social mobilisation and community sensitisation showing their support of the mapping survey in the community and to highlight the benefits of participating in the survey
   b. **Field Workers** should provide specific sensitisation on:
      i. LF – what it is and how it is transmitted
      ii. Aim and purpose of mapping
      iii. Methodology and information collected
      iv. Diagnostics used
      v. Benefits from participation (including availability of treatment from local health facility)
      vi. Ethical considerations and selection criteria
      vii. Time required to conduct mapping
   c. Answer questions from community with support from **Team Leader, Translator**
   d. Recruit and direct participants who voluntarily agree to take part to field laboratory
      i. It may be time efficient to allocate time slots for individuals to come back to the mapping survey laboratory rather than waiting to be tested. An example of how the grouping system could work can be seen below:

TIME SLOTS: *Participants are divided in 25 people groups. Eachgroup is given a time slot for when the participants should to return to the mapping survey laboratory.*
The first group should be individuals who are not requested to return but will be tested there and then. In the time slots below, 9am should be regarded as the group that are not requested to come back later.

- **Group 1 – 9am**
- **Group 2 – 11am**
- **Group 3 – 1pm**
- **Group 4 – 3pm**

5. Conduct Mapping Survey (testing 100 eligible individuals)
6. End mapping
7. Inform community leaders that the mapping has been completed (if required)

Additional Notes:

(Write below any additional notes from the facilitator)

………

………

………

………

………

....
....
....
....
....
....
....
....

Mapping Survey

After the initial community sensitisation, the **Team Leader**, **Translator** and one **Field Worker** should return to the field laboratory to carry out their duties during the mapping survey.

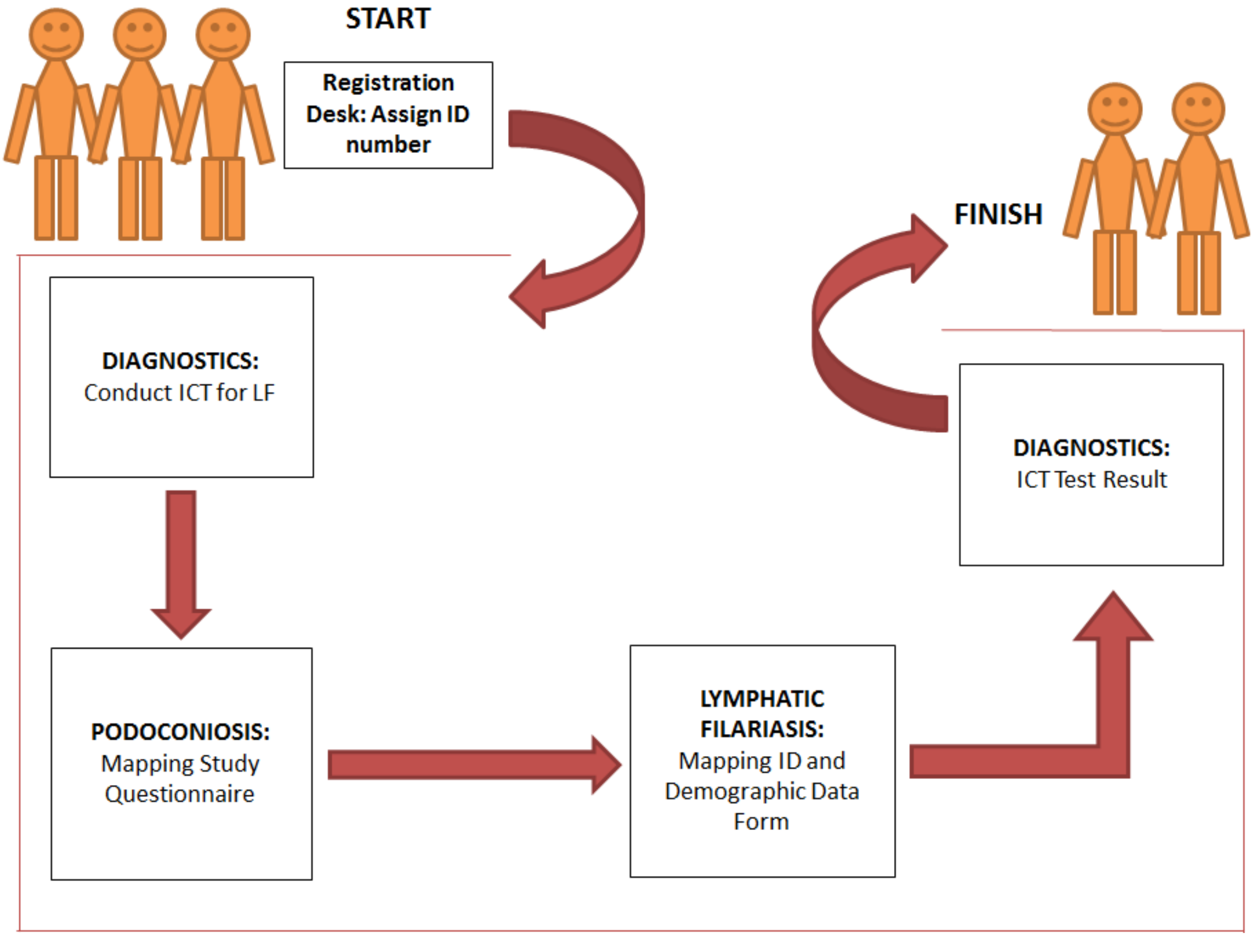

Figure 1: How the Mapping Survey should be set up

1. One**Field Worker** must be stationed at the registration desk as the first point of contact during the mapping survey.

Their main role will be to ensure participants;
- Meet the eligibility criteria
- Are able to provide full signed consent (adult/ adult + assent)
- Are allocated a unique id number
    - The last three numbers of the ID number should be on a piece of paper or the participant's hand for them to keep hold of and present at each data collection point (ICT card, LF mapping survey and podoconiosis survey)
- Further questions regarding the mapping are answered fully

N.B. Participants should wait at registration until they are able to proceed in the mapping process. It is advisable not to register too many participants at once as this can increase the potential for errors in data collection. The second **Field Worker** should try to recruit individuals and encourage them to be patient whilst waiting to be seen.

2. After registration, the participants will visit the **Laboratory Technician**.
   It is essential that the following steps are followed:
    - Provide an overview of what the test is and how it will be conducted, showing all equipment that will be used to conduct the test
    - Answer any questions regarding the test or the survey (participants will be fully informed about the survey prior to reaching the ICT card stage so general questions are not expected)
    - Follow the standard operating procedure for ICT cards to collect the blood sample and conduct the test
    - Write the ID number of the participant on the ICT card
    - Provide a cotton wool to apply pressure on the puncture site (if required/requested by participant) after the blood has been collected
    - Write on the ICT card the time when the test is started (when the blood reaches the pink part of the test). The test will be ready to be inspected after 10 minutes
    - Inform the participant that they will be told the result by the **Team Leader** and it will take 10 minutes for the result to become clear

3. Participants will then move onto the **LF Nurse** to answer a series of pre-set questions. The Nurse must also conduct a physical assessment of the individual to detect physical signs and symptoms of LF.

4. Following the LF survey, the participant will move to the **podoconiosis Nurse** to answer a further series of pre-set questions. *(Further details on this section of the survey will be provided in the podoconiosis training).*

5. The ICT card result requires 10 minutes and it is anticipated that the test results will be available after **BOTH** questionnaires are complete. The **Laboratory Technician** conducting ICT cards should write the result of the test on the ICT card as soon as 10 minutes have passed. The **Team Leader** should collect the results and speak to all participants individually to deliver the results.

6. The **Team Leader** will discuss the results of the diagnostic test and provide further information if the test is positive.

- Individuals who are **POSITIVE**should be given a standard letter which states their result. They should be directed to the local health facility where they can receive free treatment, upon presenting the letter.
- Individuals who are **NEGATIVE** will require no further medical attention or treatment and are free to leave the mapping survey laboratory.

IN ABSENCE OF TREATMENT IN SITE: *If the local health centre does not have any treatment available for individuals who test positive, then the team leader must speak with the person in charge of the health centre to ensure individuals can be treated at a later date. The health centre will be requested to collect participants' names to ensure that treatment is allocated appropriately. If participants are not happy to provide their names, they should then be advised to bring the letter provided by the mapping survey team when they come to the health centre at a later date.*

The Team Leader should contact the LF Programme Manager at FMOH immediately and inform them that treatment is not available at the health centre and there are individuals who have tested positive by ICT card (Treatment is required if a minimum of 1 out of 100 people are found to be positive). The FMOH will be responsible for arranging transportation of drugs to the health facility and the individual in-charge of the health centre should be contacted by FMOH with further information regarding delivery.

The Team Leader must ensure the following information is provided to the FMOH LF Programme Manager:

- *Name of the Health Centre*
- *Province, Woreda and Kebele of the Health Centre*
- *Name of person in charge of the Health Centre*
- *Contact details of the person in charge of the Health Centre*
- [illegible]

7. Once the participant knows their ICT card result, they are free to leave the mapping survey laboratory as their assessment has been complete.

Participants may have questions at any stage of the survey and all field team members must be prepared to answer them fully. If they do not know the answer, then the participant should be referred to the **Team Leader** who will be able to answer it.The **Team Leader** must also oversee all activities taking part in the mapping, ensuring the mapping protocol is followed and results are accurately captured.

The **Translator** must assist in all parts of the data collection where there is a language barrier. They may be required to translate disease specific information which must be translated accurately whilst remaining sensitive to cultural and language differences.

The **Field Workers** should work together; one bringing people from the community into the mapping survey laboratory, whilst the other registers them to conduct the testing. These roles are interchangeable and Field Workers may consider changing positions during the mapping.

Additional Notes:

(write below any additional notes from the facilitator)

………
………
………
………
………
……………………………………………………………………………………

4. Standard Operating procedure for ICT Cards

The ICT card test is a widely used sensitive tool for the detection of *W.bancrofti*antigen.

These tests are simple to use but require training to reduce the variability between observers and any misreading of the cards resulting in false positive results.

For the purpose of this mapping survey, only the Laboratory Technician in the field team will be conducting the LF diagnostic tests.

Guidelines
Storage and Transportation

1. At optimal storage conditions (4^0C), cards have an approximate shelf life of 9 months. However when stored at 30^0C the shelf life decreases to 3– 6 months.
2. When transporting cards to the study locations, it is advisable not to expose them to extreme heat for prolonged periods of time as this will rapidly decrease the shelf life.

Sample Collection

1. Put on a fresh pair of gloves.
2. Clean the site of the finger prick on the participant using a disinfectant wipe.
3. Using a lancet to prick the participant's finger, draw a small volume of blood.
4. From this prick, collect 100μL of blood using a capillary tube (supplied with ICT card).

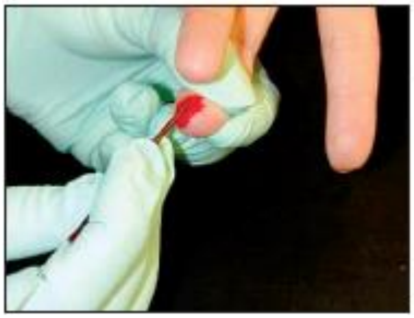

Collect 100 μl blood finger prick using a calibrated capillary tube OR measure 100 μl of blood from a microcentrifuge tube using a micropipettor. DO NOT add blood directly from the finger to the card.

5. Add the collected blood sample to the white portion of the sample pad.
    - DO NOT add blood directly to the pink portion.

- DO NOT close the card before the sample migrates to the pink portion; takes roughly 30 seconds.
- Record the time when the blood reaches the pink portion on the card.
    - The test takes 10 minutes, starting from when the blood reaches the pink portion.

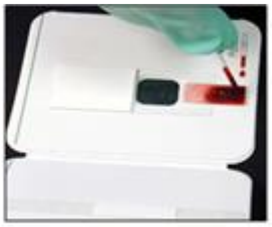

Add blood sample slowly to the white portion of the sample pad

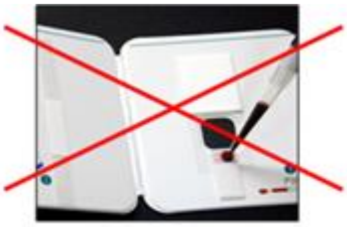

DO NOT add blood directly to the pink portion of the sample pad

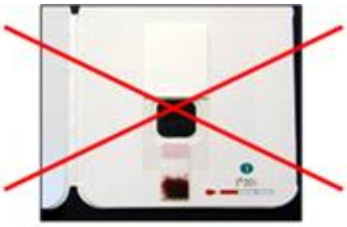

DO NOT close the card before the sample migrates to the pink portion of the sample pad (takes approximately 30 seconds after adding blood)

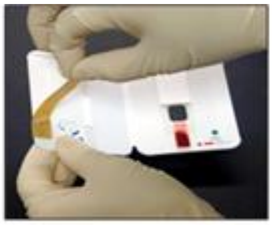

Remove adhesive liner and close card. Start timing.

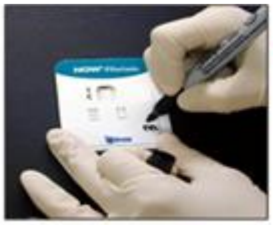

NOTE: It is helpful to record the starting time on the front of the card

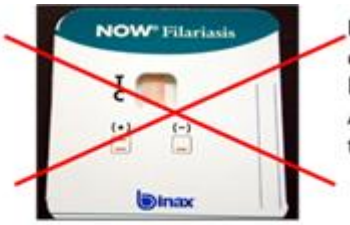

DO NOT read cards if the plasma has not flowed ALL the way down the strip.

NOTE: If plasma fails to migrate completely past the bottom of the window, a false positive result can occur

6. Read the results after 10 minutes and record the result by marking the card as positive or negative
    - DO NOT read the results at any other time as it can increase the chance of false positives
    - Ensure cards are read in a well-lit location, faint lines can be difficult to read if lighting is poor.
7. Safely dispose of the ICT card, capillary tube with any remaining blood found in the capillary tube
    - ICT card: ICT waste bag
    - Capillary Tube with any remaining blood: Sharps bin

Examples:
Below are example results from ICT cards showing the various results that can be seen on the card.

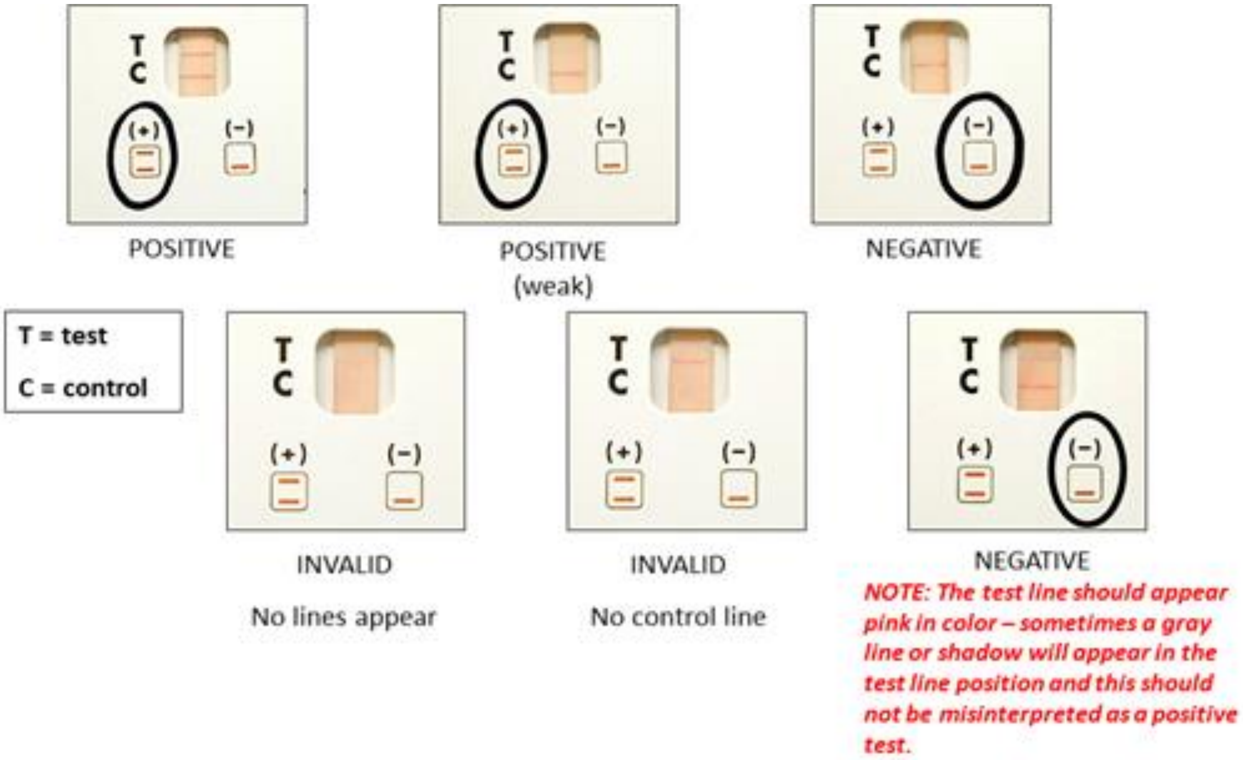

Reference:
World Health Organization, **2011**; *Global Programme to Eliminate Lymphatic Filariasis: Monitoring and Epidemiological Assessment of Mass Drug Administration*

5. Data Collection using mobile Phones (to be updated once forms finalised)

SELECTING FORMS

To fill in each form the team leader/nurse will open the Task Force LINKS app on the android phone and click on the **'Fill Blank Form'** option and then the correct form; the community form, LF form, Podoconiosis form or the ICT result form.

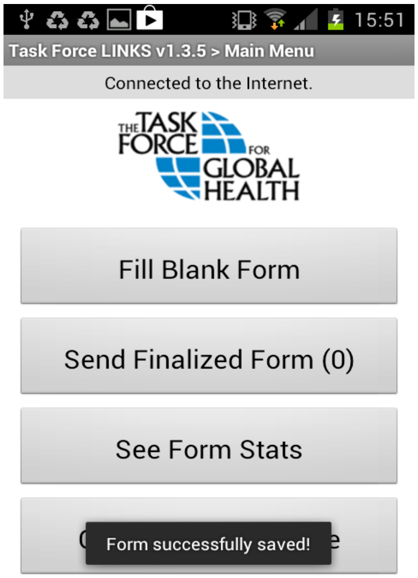

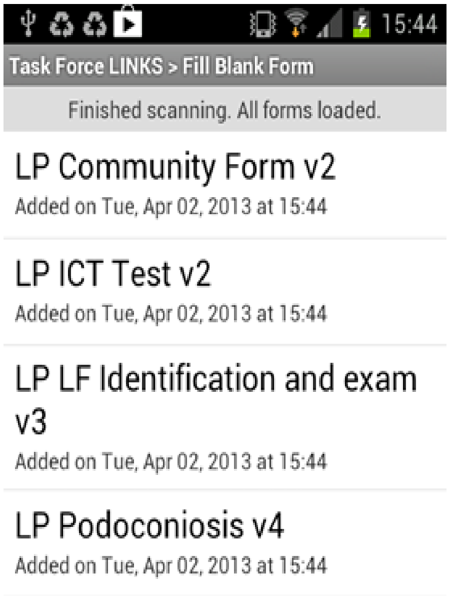

COMMUNITY FORM

The community form is used to collect all the relevant community information ranging from the name and GPS location to the population and community leaders name. This form should be collected by the field team leader upon the arrival at the survey community when they speak with the community leader prior to the survey start.

To fill in this form the community leader will open the Task Force LINKS app on the android phone and click on the **'Fill Blank Form'** option and then the **'Community Form'**. This will open the community form with the following screen:

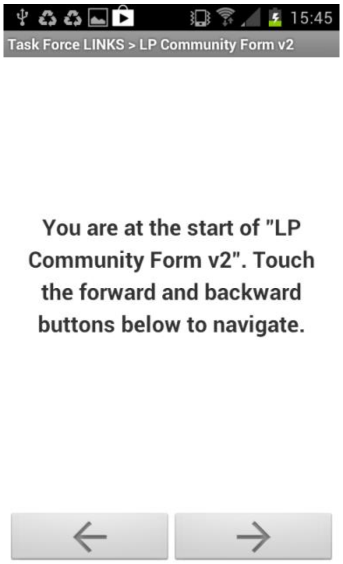

To start the survey the data collector will use the "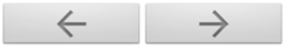" to go forward and back while they collect the information.

The following fields will appear in order as the record collector enters the information and used the navigational arrows:

1. **Record Taker's Name:**
   - The record taker needs to type in their Name into the provided field
     ie: **John Smith** would type in **John Smith** into the provided field
2. **Region:**
   - Type in the Region Name
3. **Zone Name:**
   - Type in the Zone Name
4. **Woreda Code (001-692)**
   - Enter the 3 digit code used to identify the District/Woreda; ensure that you enter the 0 or 00 prior to the district number if it is needed; districts 1-9 require 00 before the number while districts 10-99 require 0 before the district number.
     ie: the district code for first district is **001**
5. **Kebele Name:**
   - Type the kebele name in full
6. **Community Code (0001-1384)**
   - Enter the 4 digit code used to identify the Community; ensure that you enter the 0 or 00 prior to the community number if it is needed; communities 1-9 require 00 before the number while communities 10-99 require 0 before the district number.
     ie: the community code for first district is **0001**
7. **GPS Coordinates:**
   - Click the 'Record Location' button on the screen of the phone and wait for the GPS coordinates to be recorded.
8. **Name of Kebele Leader:**
   - Record the full name of the Kebele leader
9. **Kebele Leader's Contact Number:**
   - Record the mobile number of the Kebele leader
10. **Total Community Population:**
    - Record the total population number as provided by the community leader
11. **Has this community received treatment for LF in the last year?:**
    - Select one of the following option in accordance with the community leaders response: Yes, No, Don't Know
12. **Has this community received deworming treatment in the last year?:**
    - Select one of the following option in accordance with the community leaders response: Yes, No, Don't Know

Once all the information has been filled out a summary page will appear were you can double check all the information. If all the information is correct click the **'Save and Send Form'** button. This will finalize the form and return you to the home screen.

LYMPHATIC FILARIASIS INDENTIFICAION AND EXAM FORM

The 'LF identification and exam form' is used to collect all the relevant information pertaining to the mapping of LF throughout the county. This form will be filled in by one of the nurse on the field team and must be done for each participant.

To fill in this form the nurse will open the Task Force LINKS app on the android phone and click on the **'Fill Blank Form'** option and then the **'LF identification and exam form'**. This will open the community form with the following screen:

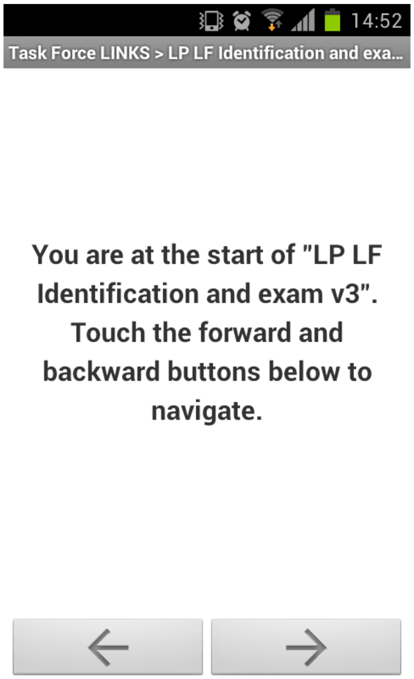

To start the survey the data collector will use the "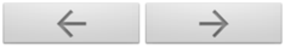" to go forward and back while they collect the information.
The following fields will appear in order as the record collector enters the information and used the navigational arrows:

1. **Record Taker's Name:**
   - The record taker needs to type in their Name into the provided field
     ie: **John Smith** would type in **John Smith** into the provided field
2. **District Code (001-692)**
   - Enter the 3 digit code used to identify the District; ensure that you enter the 0 or 00 prior to the district number if it is needed; districts 1-9 require 00 before the number while districts 10-99 require 0 before the district number.
     ie: the district code for first district is **001**
3. **Community Code (0001-1384)**
   - Enter the 4 digit code used to identify the Community; ensure that you enter the 0 or 00 prior to the community number if it is needed; communities 1-9 require 00 before the number while communities 10-99 require 0 before the district number.

ie: the community code for first district is **0001**

4. **Individual ID Number (001-100)**
    - Record the individual's personal identification number; this should be found written on their hand.
5. **Age (15-100)**
    - Record the age of the individual in years
6. **Gender**
    - Select the correct gender for the participant; Male or Female
7. **Religion**
    - Select the correct religion for the participant: Muslim, Christian, Animist or Other
8. **Ethnic Group**
    - Select the correct ethnic group that the individual belongs to from the provided list
        - Oromo, Amhara, Somali, Tigre, Afar or Other
    - **If Other selected:** type in response
9. **How long have you lived at your current location?**
    - Record the amount of time the individual has lived in location (in years)
10. **Years of School Completed**
    - Record the number of years that the participant went to school
11. **Did you use a bed net last night?**
    - Select the correct answer to the question: yes or no
12. **Was the net treated with insecticide when you obtained it?**
    - Select the correct answer to the question: Yes, No, Don't Know
13. **Presence of Pain**
    - Select the correct answer to the question: yes or no
14. **Presence of Swelling**
    - Select the correct answer to the question: yes or no
15. **Presence of Redness**
    - Select the correct answer to the question: yes or no
16. **Presence of Fever**
    - Select the correct answer to the question: yes or no
17. **Presence of Nausea**
    - Select the correct answer to the question: yes or no
18. **Presence of Vomiting**
    - Select the correct answer to the question: yes or no
19. **Presence of Lymphedema**
    - Select the correct answer to the question: yes or no
    - **Presence of Lymphedema – Upper Limb: will only appear if 'yes' is selected for question 19**
        - Select the correct answer to the question: yes or no
        - **Site: if Yes selected:** Select the correct answer; bilateral or unilateral
    - **Presence of Lymphedema – Lower Limb: will only appear if 'yes' is selected for question 19**
        - Select the correct answer to the question: yes or no
        - **Site: if Yes selected:** Select the correct answer; bilateral or unilateral

- **Presence of Lymphedema – Breast: will only appear if 'yes' is selected for question 19**
  - Select the correct answer to the question: yes or no
  - **Site: if Yes selected:** Select the correct answer; bilateral or unilateral
- **Presence of Lymphedema – Vulva/Penis: will only appear if 'yes' is selected for question 19**
  - Select the correct answer to the question: yes or no
  - **Site: if Yes selected:** Select the correct answer; bilateral or unilateral
- **Presence of Lymphedema – Hydrocele: will only appear if 'yes' is selected for question 19**
  - Select the correct answer to the question: yes or no
  - **Site: if Yes selected:** Select the correct answer; bilateral or unilateral

20. **Presence of Chyluria (Milky Urine)**
    - Select the correct answer to the question: yes or no
    - **Site/Note (Optional) if Yes selected:** Record any notes of interest, keep to a max of 10 words
21. **Presence of Haematuria**
    - Select the correct answer to the question: yes or no
    - **Site/Note (Optional) if Yes selected:** Record any notes of interest, keep to a max of 10 words
22. **Other?**
    - Select the correct answer to the question: yes or no
    - **Site/Note (Optional) if Yes selected fill out what the other is**

Once all the information has been filled out a summary page will appear were you can double check all the information. If all the information is correct click the **'Save and Send Form'** button. This will finalize the form and return you to the home screen.

ICT CARD RESULT FORM

The **'ICT card result form'** is used to collect all the relevant information pertaining to the mapping of LF throughout the county. This form will be filled in by one of the field team members at the end of the day after all the other forms have been filled in; this must be done for each ICT card.

To fill in this form the nurse will open the Task Force LINKS app on the android phone and click on the 'Fill Blank Form' option and then the **'ICT card result form'**. This will open the community form with the following screen:

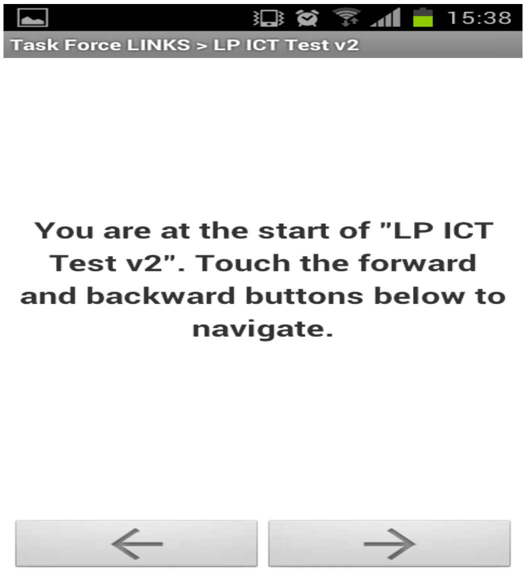

To start the survey the data collector will use the "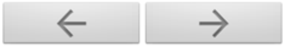" to go forward and back while they collect the information.
The following fields will appear in order as the record collector enters the information and used the navigational arrows:

1. **Record Taker's Initials:**
   - The record taker needs to type in the initials into the provided field
     ie: **J**ohn **M**athew **S**mith would type in **JMS** into the provided field
2. **Individual ID Number (001-100)**
   - Record the individual's personal identification number; this should be found written on their hand.
3. **ICT Results:**
   - Select the correct answer to the question: Positive, Negative or invalid
   - **Add a note about this test? (Optional):** Record any notes of interest, keep to a max of 10 words

Once all the information has been filled out a summary page will appear were you can double check all the information. If all the information is correct click the **'Save and Send Form'** button. This will finalize the form and return you to the home screen.

6. Checklist for Conducting survey

For each participant, it is essential to ensure the following:

ID Number – General

1. Allocate the ID number to each participant at the beginning
2. Ensure no name and/or address is collected at any point and matched with an ID number
3. The ID number should be written on ICT card prior to conducting test

4. ID number on ICT card must correspond to ID number on forms for podoconiosis and LF
5. Participant's ICT result must be entered onto participant's corresponding forms for podoconiosis and LF
6. ID numbers on podoconiosis form and LF form for one participant must identical

Laboratory Technician – Health & Safety

1. Ensure the work bench is cleaned with bleach periodically to keep the work area clean
2. Alcohol swabs after single use must be discarded in the general waste bag
3. Lancets, once used, must be discarded in the sharps bin
4. ICT cards, once results have been recorded, must be discarded in a waste bag specific for ICT cards
5. Capillary tubes, used to conduct ICT cards, must be discarded in sharps bins once used, irrespective of whether there is still blood in the tubes or not.
6. Broken capillary tubes must not be used and discarded in the sharps bins
7. Gloves must be worn at all times when conducting diagnostic tests
8. Ripped/torn gloves must be replaced immediately
9. Gloves should be replaced regularly throughout the testing
10. Use hand sanitizers regularly and use soap to wash hands before and after conducting ICT cards
11. Waste bins for biological samples should not be mixed with any other waste
12. Cotton wool balls should be provided to participants when requested, or when the wound is still bleeding
13. Biological samples in the waste bags must be incinerated before leaving the site
14. Sharps bins should be used until they are full. Once full they should be closed and stored until returning to central level for safe disposal

Laboratory Technician – Laboratory Procedures

1. Follow standard operating procedures for conducting ICT cards
2. Write the time when the blood reaches the pink portion of the test onto the card
3. Read ICT card after 10 minutes and write result on the test immediately
4. Results should only be shown/discussed to the Team Leader and not discussed with any participants in the survey

Nurses –Laboratory Procedures, Health & Safety

1. Answer all questions in the questionnaire
2. Ensure ID number entered accurately into mobile devices
3. Use hand sanitizers regularly
4. Wash hands with soap before and after examining patients for physical signs or symptoms of LF
5. Record ICT card result before storing data and moving onto the next participant

7. Frequently asked questions

1. **The community leaders are not happy to have the survey conducted at the site – what should we do?**

The Team Leader should discuss further why the Community Leaders are not happy for the survey to be conducted and highlight the benefits from participating. The Team Leader should also consult with the Translator if there are any language barriers to ensure the purpose of the survey is clear. If the Community Leaders are still not happy for the survey to be conducted there, FMOH should be informed and consulted on selecting another community to survey within the district.

2. **The community leaders have agreed to the survey, however the community are not happy to participate – what should we do?**

The Team Leader, Translator and Field Workers should sensitise the community fully and address all questions the community have. The Team Leader should also provide comprehensive information on the purpose of the survey and the benefits the community will receive; free treatment and knowledge on their disease status. The Community Leader should also be encouraged to speak to their community to engage and motivate them and emphasise why they should participate. If there is still significant resistance to the survey being conducted, FMOH should be informed and another site selected.

3. **Members of the community are spreading false rumours about the mapping survey – what should the mapping team do?**

The Team Leader, Translator, Field Workers and Community Leaders must control these rumours immediately and accurately inform the community about the mapping survey and the purpose. The FMOH will provide each team with a stamped and signed letter showing that the activity is being conducted by the government. It will be Team Leader's responsibility to ensure that such events are controlled and this does not affect the mapping survey.

4. **What do I do if participants get sick of waiting to be seen for the mapping survey?**

All participants agreeing to take part in the survey will have to wait. Unfortunately this is due to the time required to get a result from the ICT card and to fill in the questionnaires. The proposed Time Slot method should cut down on lengthy waiting times. The Field Workers should discuss this with the community members waiting to be seen and inform them that once they start in the survey process, it will be complete very quickly. Participation is voluntary and if individuals feel they no longer want to participate they are free to leave at any point. The field team should work together closely to monitor the number of people tested and if the required sample size is not met, Field Workers should recruit more individuals.

5. **What do I do if the participants do not return to their allocated time slot?**

Participation is voluntary and if members of the community do not return then the Field Workers should recruit new people to replace them. If participants return later than their time slot, they should be allowed to participate, providing the sample size has not yet been met. Timing is important and it is the Field Worker's responsibility to ensure that people are always waiting to be seen until the sample size is met. If team members are sitting idle, it is a waste of the field team's time and it will lengthen the time required to sample the site.

6. A participant originally said yes to taking part however has changed their mind during the survey – what do I do?

Participants are able to change their mind at any stage within the survey process. Field team members should speak to individuals who are considering leaving the study to ask why they are leaving and provide any additional information which may alter their decision to do so. It is essential that field team members are respectful to the participants and do not feel forced to take part in the survey. Team members should emphasize that participation is voluntary and leaving the survey will not have any impact on any health benefits they may have received prior to the or after the survey.

7. The GPS device is not finding our location – what do we do?

GPS devices are imbedded within the smartphones used to conduct the data collection.They provide location and time information when they are in unobstructed line of sight to a minimum of three GPS satellites. If the smartphone is not able to detect the location, this would suggest that enough satellites are not in sight and you should move to a better place. This could involve taking two steps forward, or it could mean changing where you take the GPS point entirely. For the purpose of this survey, a central location within the site with no obstructions (tall buildings or trees) would be ideal.

8. The mobile phone is not responsive, what should we do?

If the mobile device is not responsive for any reason, please wait five minutes and see if the phone recovers. If the phone does not start working again, it will need to be restarted. Any data forms that were not completed before the phone crashing will be lost and therefore data will have to be inserted once more. This should be a rare occurrence and should not be experienced regularly. If the phones are found to be problematic or not functioning for any reason, the Team Leader will be carrying paper copies of the questionnaire which should be filled out by the team instead.

Annex 6.
Training manual for Podoconiosis mapping in Ethiopia

I. Background

Podoconiosis (endemic non-filarial elephantiasis) is a non-infectious geochemical disease arising in barefoot subsistence farmers who are in long-term contact with irritant red clay soil of volcanic origins. The disease causes progressive bilateral swelling of the lower legs. Mineral particles absorbed through skin are taken up into macrophages into the lymphatic system and result in an inflammatory process leading to fibrosis and obstruction of the vessels. This leads initially to swelling of the foot and the lower leg, which progresses to elephantiasis: gross lymphoedema with mossy and nodular changes of the skin. Podoconiosis affects some 4 million people in Africa, Latin America, and a few areas of Asia. It is found in more than ten countries across tropical Africa where irritant soils have been generated by environmental conditions of high altitude (>1,000m) and high annual rainfall (>1,000mm), and are farmed by very poor people who cannot afford shoes or water.

The current control strategy for podoconiosis consists of integrated approach prevention among school age children through promotion and distribution of protective shoe and lymphedema management. The main challenge in facing podoconiosis is lack of adequate data to inform policy makers, program planners and donors about the distribution of podoconiosis. Therefore this project was planned to generate distribution map of podoconiosis in Ethiopia. The output would help to target resources, monitor progress, and advocate for investment in podoconiosis prevention control and ultimately elimination.

i. Introduction

Elephantiasis: Elephantiasis is a term commonly used to describe lymphedema affecting a limb so that it becomes swollen, resembling that of an elephant. Lymphedema is a condition in which sections of the lymphatic system malfunction and block proper drainage of lymph. This in turn results in tissue inflammation and enlargement of the affected limb or area. Elephantiasis is very uncomfortable and disfiguring. In the tropics, there are two major types of elephantiasis, and, while the diseases do have some similarities, there are also significant differences.

Lymphatic Filariasis (LF): The first type is Lymphatic filariasis (LF). This is the type of elephantiasis that first comes to most people’s minds when confronted with a swollen leg in the tropics. Lymphatic filariasis is a communicable disease transmitted between persons by mosquitoes that transmit filarial parasites. The parasites cause inflammation and subsequent malfunction of the lymphatic system, blocking normal lymph drainage. This blockage results in a vast enlargement of the affected limb. LF can occur in various

body parts and most commonly occurs in the affected individual's feet, legs, arms, scrotum, vulva, or breasts.

Non- Filarial Elephantiasis (Podoconiosis): The second type of elephantiasis is Non-Filarial Elephantiasis or podoconiosis. In lay terms, the disease is also called Mossy Foot because of the moss-like skin changes that often occur on the feet. Podoconiosis is a non-communicable disease that is acquired through prolonged exposure to red clay soils of volcanic origins when tiny silica crystals appear to be absorbed through the feet. The disease occurs primarily where these red clay soils are found in areas of high altitude (over 1000m), high seasonal rainfall (over 1000mm per year), where most people are subsistence farmers.

Podoconiosis has been described in ten countries across tropical Africa, and has also been reported in tropical areas of Central America and Northern India. The exact global burden is still to be measured, but it is estimated that at least 4 million people are affected worldwide.

Not only is the causal agent of podoconiosis different from that of LF, but it also differs in several other ways. The limb swelling is almost always restricted to the feet and lower legs below the knees. The first signs of disease are often in the foot, with lymph nodes in the groin affected later (in LF groin involvement is earlier). Podoconiosis is preventable through the proper regular use of footwear and simple foot hygiene. Although, there is no "cure" for podoconiosis, it can be controlled and reversed through a variety of simple means to be discussed later. If continually treated properly, podoconiosis patients are able to return to a normal way of life.

The disease typically manifests itself between the ages of 10 and 30, but individuals as young as 5 may experience early symptoms. In addition, genetic studies show high heritability of the trait that causes podoconiosis. Podoconiosis is typically observed in rural subsistence farmers whose trade, culture, and economic circumstance often make it uncomfortable, against cultural norms, or a financial burden to wear shoes. Further, there is often stigma associated with podoconiosis sufferers. They are often excluded from religious meetings, government meetings, school, and other community gatherings. Sufferers and/or sufferers' siblings are often barred from marrying into unaffected families.

Podoconiosis has significant economic impact; according to a study in Ethiopia it was found out that the disease results in productivity loss. It is estimated that 1 million cases are found in Ethiopia and 11 million people are at risk, living in 18% of the land of Ethiopia. Total direct costs of podoconiosis amounted to the equivalent of US$ 143 per patient per year. Total productivity loss for a patient amounted to 45% of total working days per year, and in a zone of 1.5 million people, the total overall annual cost of podoconiosis was calculated to exceed US$ 16. million per year. In addition the disease is known to be stigmatized with significant social exclusion. Podoconiosis is widely distributed in three continents; Africa,

Central America and Asia particularly India. In Africa, at least 10 countries with the disease have been identified. Previous studies have documented the association of the disease with irritant red clay soils, which are generated in areas at >1500 metres above sea level (masl), with >1000 mm annual rainfall.

In Ethiopia, prevalence estimates from 56 market counts ranged from 0.42% to 3.73% these rates are probably underestimates because podoconiosis is a stigmatized disease that may keep affected people from attending public gatherings and the debility the disease causes reduces their mobility. More recent studies in Ethiopia estimated a prevalence of 5.46% in Southern Ethiopia, 5.2% in western Ethiopia, 7.4% in central Ethiopia and 3.3% in northern Ethiopia.

ii. Prevention

- Education and Communication of Information
- Shoes for prevention among children

iii. Treatment

Basic Podoconiosis Treatment

There are six components to treatment of podoconiosis. These are:

SN	Treatment	Description
1	Foot hygiene	Foot hygiene is important because it removes soil particles from the foot, reduces bacterial load and restores the function of the skin. It is important that the feet are thoroughly washed and dried daily. The first step is to soak the feet for 15-20 minutes in a basin with cool, clean water and diluted antiseptic.
2	Skin care	After washing and drying the foot and leg, it is essential to rub the skin with local oil or ointment to keep it supple. In Wolaita, the MFTPA uses Whitfield ointment which is relatively inexpensive and easily attainable from Addis Ababa. In some other areas locally acquired, clean cooking oil with eucalyptus or neem extract is used.
3	Bandaging;	The leg should be bandaged from the toes towards the knee, ideally when the leg is elevated. The foot should be wrapped using a “V” shaped design and then bandaging continued in a spiral up the leg to 5cm above the upper limit of swelling, overlapping the previous layer by 50% each full turn around the leg. In Wolaita, MFTPA provides patients with two bandages per affected leg so that one may be washed while the other bandage is in use.
4	Socks and shoes	Socks and shoes are vital for both treatment and prevention of podoconiosis.
5	Elevation and movement	Movement or exercise and elevation of the leg can help improve lymph circulation and reduce swelling. Ankle circles and calf raises are recommended but should be suspended if they cause pain.
6	Surgery	Surgery is becoming increasingly uncommon among podoconiosis patients, since the vast majority of patients benefit from the simple treatment above. Very few patients benefit from surgery, which should be limited to removal of discrete nodules that are preventing the patient wearing shoes. Nodule removal may be followed by limited skin grafting.

iv. Clinical Staging of podoconiosis

Clinical staging is important for several reasons. First, it allows program planners to assess the burden of disease in an area. It also gives patients feedback during self-treatment, allowing a patient to see his or her progress. Staging also offers a means for health professionals to record the effectiveness of the treatment, and for researchers to document the effectiveness of an intervention or program.

There are several key words to define and understand before discussing the different stages of podoconiosis. For the sake of staging, these key words are defined as follows:

- Swelling a general increase in the size of the foot or leg.
- Knob/bump a discrete hard lump seen or felt to protrude.
- Moss tiny, rough lesions around the base of the foot that resemble moss.
- Ankle the level of the two ankle bones when standing.
- Knee the level of the top of the knee cap when standing.
- Circumference the greatest below the knee measurement in centimeters.

Staging of podoconiosis was developed from the Dreyer system for staging LF. The Dreyer system of staging did not fit the clinical picture of podoconiosis perfectly, thus the following system was developed to stage podoconiosis and is used widely in Ethiopia. The stages are:

- Stage 0 No disease is present.
- Stage 1 Swelling of the limb is reversible overnight. Swelling is not present when the patient first wakes up but sets in as the day progresses.
- Stage 2 Persistent below-the-knee swelling that is not reversible overnight. If present, knobs and/or bumps are below the ankle only.

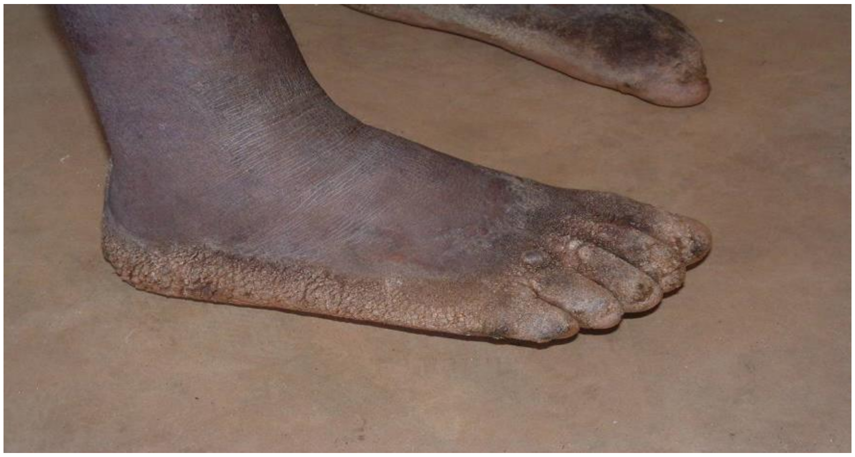

Stage 3 Persistent below-the-knee swelling that is not reversible overnight. Knobs/bumps are now present above the ankle.

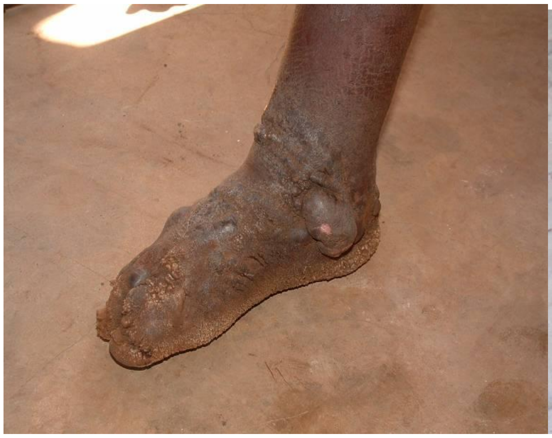
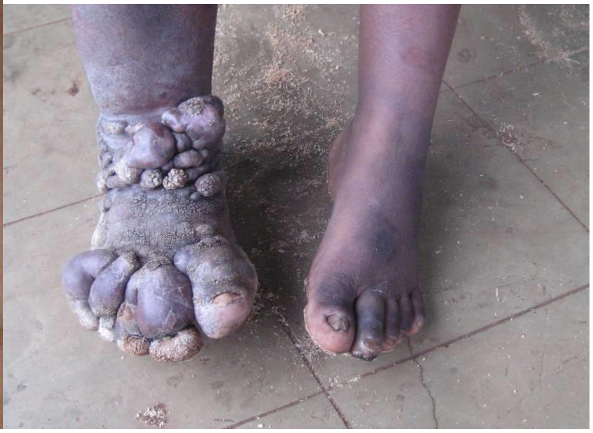

• Stage 4 Persistent above-knee swelling. Knobs/ bumps are present anywhere on the foot or leg.

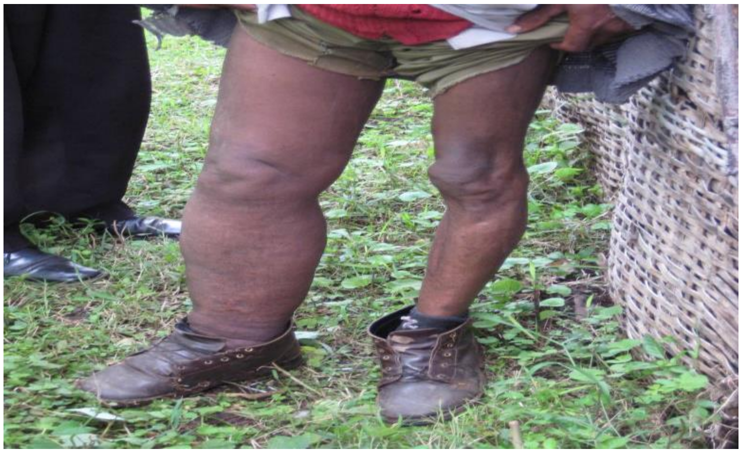

• Stage 5 Joint fixation and swelling at any place on the leg. The ankle and toe joints are fixed and difficult for the patient to flex.

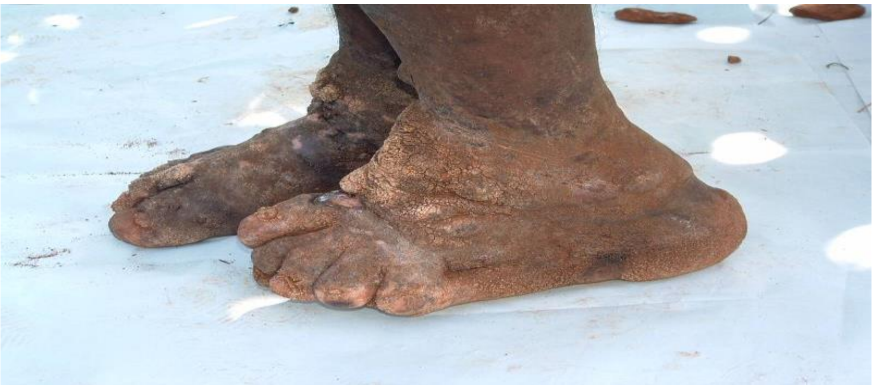

Generally each leg is staged independently of the other. First, the clinical stage of the leg is recorded using the above system. Next, presence or absence of moss is documented, using ‘M+’ if moss is present, or ‘M-‘ if absent. Wounds are checked for, and the observation is recorded as follows: ‘W+’ if

present and 'W-' if not. Finally, a flexible tape measure is used to measure the circumference of the largest point of swelling below the knee.

A patient with stage 3 podoconiosis, moss, no wound, and a 34cm greatest below the knee circumference would be recorded in the following way: 3, M+, W-, 34.

v. Differential Diagnosis

When diagnosing and treating podoconiosis, there are several other diseases causing swollen legs that must be considered:

SN	Disease	How to differentiate?
1	Filarial lymphoedema (LF);	Where is the patient from? LF only occurs at altitudes of less than 1000 meters, where the mosquitoes that transmit it can survive. It cannot be transmitted at higher altitudes (dega and woina dega) where podoconiosis occurs.<br>Where did the swelling start? The swelling of podoconiosis starts in the feet and proceeds up the leg; that of LF starts at the groin and proceeds down the leg.
2	Leprosy;	Leprosy is frequently found in podoconiosis areas, and one of the consequences of long-term leprosy may be leg or foot swelling. The most important way of distinguishing the two conditions is to test for sensation in the foot. The leprosy disease process causes decreased sensation in addition to swelling, whereas in podoconiosis, sensation remains intact. If examination for skin depigmentation and thickened nerves suggests leprosy, the patient must be referred for anti-leprotic therapy.
3	Onchocerciasis;	May sometimes be found in areas in which podoconiosis is also endemic. Skin changes (itchy skin rash and 1-2cm nodules or bumps) on the trunk and arms are common in onchocerciasis. In some countries, eye changes are common in onchocerciasis, but these changes are rare in Ethiopia. Patients suspected to have onchocerciasis must be referred on.
4	Malnutrition in children;	May present with swollen feet, and malnourished children have at times been brought to podoconiosis clinics. Other signs of malnutrition include 'rusty' hair discolouration, swollen belly, shiny taut skin and lack of energy. Malnourished children require referral for specialist management.
5	Rheumatic heart disease;	Rheumatic heart disease is a condition in which permanent damage to heart valves is caused by rheumatic fever. The heart valve is damaged by a disease process that generally begins with a strep throat caused by bacteria called Streptococcus, and may eventually cause rheumatic fever. Joint inflammation - including swelling, tenderness, and redness over multiple joints. The joints affected are usually the larger joints in the knees or ankles. The inflammation "moves" from one joint to another over several days.
6	Post surgery; and	

7	Milroy's disease.	Milroy's disease is a familial disease characterised by lymphedema, commonly in the legs, caused by congenital abnormalities in the lymphatic system. Disruption of the normal drainage of lymph leads to fluid accumulation and hypertrophy of soft tissues. The defect in Milroy's disease is present from birth and symptoms are usually first experienced in childhood. The most common problem is one-sided leg swelling, unilateral edema, which is progressive and can affect both legs.

II. Purpose of the mapping

The FMoH is looking to control and eliminate podoconiosis from Ethiopia. In order to initiate the control and elimination programme, the first step is to identify areas where the disease exists. This is assessed by mapping the disease distribution using different diagnostic tools and identifying physical signs and symptoms. Once this information has been collected, the second step is to deliver treatment to all podoconiosis patients in areas endemic for the disease. Currently the treatment is free and will consist of six components as described above.

This project aimed at mapping the distribution of podoconiosis in districts which were not mapped for LF. In the study a total of 692 districts will be covered. From each districts two Kebeles will be selected and 200 individuals involved in the study. The two villages will be selected according to WHO protocol. The study will involve individuals >=15 years of age, who lived in the area for at least 10 years and didn't leave the area for more than 6 months. People who have serious acute or chronic illnesses should be excluded from the study.

III. Guidelines for field mapping activities

Mapping will include collection of a variety of information including; community information, socio-demographic data, signs and symptoms caused by podoconiosis, disease stage, shoe wearing practice and housing conditions.

Equipment

The mapping surveys require minimal equipment and aim to be rapid. Each mapping team will be provided with the following items for field surveys:

- Immuno-chromatographic test(ICT) cards
- Single Use Sterile Disposable Safety Lancets
- Alcohol
- Cotton
- Gloves
- Immuno-chromatographic test(ICT) cards
- Single Use Sterile Disposable Safety
- Hand sanitizers
- Soap
- Bleach
- Insecticide
- Safety Box
- Plastic waste bags
- Plastic sheet
- Nunc tube
- Cold box
- Nunc tub rack
- Vacutainner
- Stationary
- Mobile Phones for Data Collection
- Informed Consent Forms

Lancets
- Syringe with needle

Method

In each district, the mapping survey will be conducted in two sites that are selected by the **FIELD TEAM** based on the district/Woreda health data and reports on lymphodemas. In **EACH OF THESE SELECTED SITES,** information must be collected for a total of **100 RANDOMLY SELECTED INDIVIDUALS (50 Female and 50 Male),** aged 15 years or above.

For true representation of the disease within the community and ethical reasons, not all individuals are eligible to participate in the mapping survey and it is important to adhere to the exclusion criteria below:

- Does not meet the minimum age requirements
- Has not lived in the community for a minimum of 10 years (as the individual may not be representative of the local population)
- Travelled out for greater than 6 months
- Has not provided consent
- Is severely sick

To collect the data, the field teams will be given mobile phones with a set of questions that the data collection members (Team Leader and Nurses) will have to enter information on.

Community and Demographic Information

COMMUNITY INFORMATION: This should be collected prior to the start of the field surveys by the Team Leader. This will collect basic information of the community in the following areas; such as the GPS location of the site, community name, population size etc. The unique coding and ID system used in these forms will be pre-set and provided to the Team Leader by FMOH.

DEMOGRAPHIC INFORMATION: This should be collected during the field survey by the Podo nurses. Each individual participating in the survey will be asked a number of questions about themselves such as gender, occupation, shoe wearing, foot hygiene practice and physical signs of Podo.

Please refer to the Data Collection Using Mobile Phones section for more information regarding all data collection and entry.

Diagnostics – Podoconiosis

Presence of the LF parasite within individuals will be determined by using the ICT card, which is a rapid diagnostic tool, used commonly in LF elimination programmes. The test will be conducted by the Laboratory Technician within the field team and the results entered into the phone by the Nurses.
In this study all lymphoedema cases are suspects for podoconiosis. Therefore different history, physical examination and diagnostic test will be conducted to exclude other causes and reach into the diagnosis of podoconiosis. First they will be tested for ICT and if found negative in areas where at least one ICT positive case is found additional blood sample will be taken for antibody test. Leprosy, onchocerciasis and rheumatic heart disease will be excluded as indicated above.

Algorithm for diagnosis of podoconiosis

Figure. Sampling framework

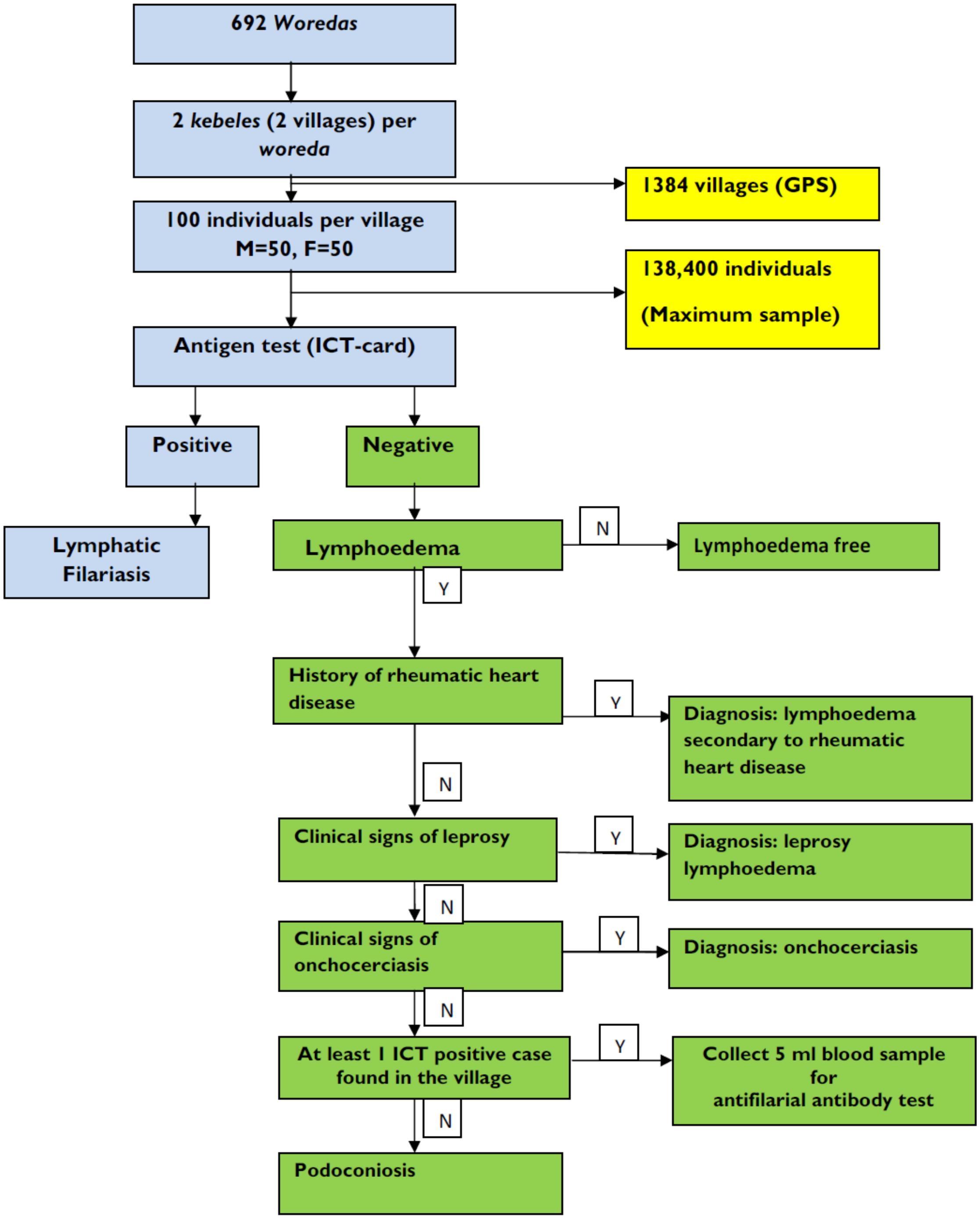

Ethical considerations

- Participation in the survey is VOLUNTARY and it is imperative that nobody within the community feels forced to take part or concerned that not participating may affect any future health benefits they receive.
- Selection of individuals to take part in the survey must be RANDOM and there should be no bias in selecting one individual over another.
- To ensure ANONYMITY, individuals will be identified by a unique identity number only and personal details such as name or address must not be requested during the mapping process.
- It is important to get written approval from the community leaders prior to starting the field surveys. This will confirm that they are informed about the survey happy for it to be conducted.
- All participants will be requested to provide WRITTEN CONSENT that they understand the purpose of the survey and voluntarily are happy to take part in the survey. Individuals who are under the age of 18 years, they will require additional consent from their parents or legal guardian.
- Individuals who are positive should be provided TREATMENT. Treatment will not be administered as part of the mapping survey and participants, who are tested positive, will be advised to visit their local health facility, where they will receive medications for free. The national programme will also distribute drugs through the MDA strategy shortly after the mapping results have been analysed.

Additional Notes:

(Write below any additional notes from the facilitator)

....

....

....

....

....

....

....

....

....

....

....

....

....

....

....

....

....

……………………………………………………………………………………
……………………………………………………………………………………
……………………………………………………………………………………
……………………………………………………………………………………
……………………………………………………………………………………
……………………………………………………………………………………
………………………

Field Teams

As an integral member of a small field team for conducting the mapping surveys, team work and coordination is essential. Field teams will be composed of individuals with a variety of skill sets each of who have specific responsibilities as outlined below:

<table>
<tr><th>Team Member</th><th>Quantity</th><th>Key Responsibilities for podoconiosis Mapping</th></tr>
<tr><td>Team Leader</td><td>1</td><td>Community
Communicate with community leaders to obtain support and consent for mapping
Communicate with community members to answer questions regarding LF, mapping and the LF elimination programme
Point of contact for community during mapping
Field Team
Lead the field team to conduct mapping surveys
Oversee surveys and supervise field team activities, ensuring all team members are following the mapping protocol
Point of contact for EPHI/FMOH
Monitor all field data collection
Manage time of field activities and time spent on each mapping site
Data Collection
Collect GPS points for mapping site
Record LF community form information
Responsible for safe keeping of mobile phones and data at all times
Inform participant of ICT result and record data into data collection form for result
Other
Advise on treatment and answer further questions regarding ICT result
Have knowledge on how to conduct all aspects of the mapping survey, including the ICT card
Activities associated with mapping e.g. assisting other field team members
Ensure local health facilities have drugs available for treatment referral or inform where FMOH where treatment cannot be provided</td></tr>
<tr><td>Nurse</td><td>2</td><td>Communication
Communicating with members of the community regarding LF, mapping and the LF elimination programme
Conducting physical examination of individuals participating in the mapping
Data Collection
Enter all information accurately into data collection systems using mobile phones</td></tr>
</table>

Team Member	Quantity	Key Responsibilities for podoconiosis Mapping
		Other<br>• Activities associated with mapping e.g. assisting other field team members<br>*(One nurse will focus on conducting the LF mapping, whist the other will focus on podoconiosis mapping.)*<br>*Podo nurse:*<br>• Collect blood sample from ICT negative lymphodema cases where the LF prevalence ≥1% using vacutainer<br>• Separate serum from the whole blood after one hour of collection<br>• Label using patient Worerd Village- individual ID number.<br>• Store in a cold box.
Laboratory Technician	1	Data Collection<br>• Prepare field lab for conducting LF diagnosis (ICT card)<br>• Collect blood sample from participant<br>• Ensuring correct allocation of ID number for participant and test<br>• Conduct ICT card test and determine ICT result<br>• Time management so ICT cards are read within the required time according to ICT card instructions<br>• Deliver ICT card results to LF Nurse and ensure capture correctly in data collection form<br>• Ensure good health and safety practices are followed for sample collection and disposal of all ICT card test material.<br>• Inform participant how the test will be conducted, what samples will be taken and what the result will show.<br>• Answer any questions associated with LF, mapping, the LF elimination programme and the ICT card test<br>Other<br>• Activities associated with mapping e.g. assisting other field team members
Translator	1	Communication<br>• Sensitise and inform the local community about LF, the mapping survey and the FMOH LF elimination programme<br>• Translate communication by mapping team member<br>• Translate communication by participant<br>• Ensure communication is sensitive to local cultural and behavioural settings<br>Other<br>• Activities associated with mapping e.g. assisting other field team members
Field Worker (Local)	2	Communication<br>• Obtain written consent from participant<br>• Sensitise and inform the local community about LF, the mapping survey and the FMOH LF elimination programme<br>• Motivate community members to participate in the survey<br>• Allocate identification (ID) number for participant<br>• Time slot allocation to ensure participants are not waiting for long periods of time unnecessarily<br>Other<br>• Activities associated with mapping e.g. assisting other field team members
Driver	1	• Safely transport all field team members and equipment from between mapping sites<br>Other

Team Member	Quantity	Key Responsibilities for podoconiosis Mapping
		• Activities associated with mapping e.g. assisting other field team members

IV. On the day activities

Generally it is expected that mapping surveys for one site should take no longer than 1.5 days. On day 1, it is anticipated that there will be no field work and main activities will include sensitising community leaders and obtaining written approval that the survey can take place - this should be half a day's work. Field work should usually be conducted on day 2; participant recruitment, data collection and diagnostic testing.

AT EACH NEW DISTRICT/WOREDA, the field team will need to visit the district/Woreda level health centre first to obtain health records and review lymphedema and hydrocele information; to identify **TWO** suitable sites for conducting the mapping survey.

Day 1

Activities require: **Team Leader, Translator, Field Workers** and **Driver**

9. Team members travel to site within district
10. Meet with Community Leaders and introduce team
11. Team leader to discuss with community leaders the following areas;
    a. **LF:** What Podoconiosis is and how it is caused?
    b. **FMOH Plans:** Initiation of national LF elimination programme
    c. **Mapping Podo Distribution:** Aim and purpose of mapping, methodology, information collected, diagnostics used, benefits from participation, ethics and time required to conduct mapping
    d. **Treatment for Podoconiosis:** If individuals are positive what they should do? What treatment should they seek?
    e. **Result of Podoconiosis Mapping:** What will happen next? Analysis of results, findings translated into treatment strategy for national elimination programme
    f. **Consent to Map:** Discuss conducting the mapping survey in their community and obtain written consent that the mapping survey can be conducted there
    g. **Location for Mapping:** Discuss where the mapping survey can be conducted (central meeting point)
    h. **Community Involvement:** Request and motivate leaders to conduct **Community Sensitisation And Social Mobilisation**; to inform their community to participate in the mapping survey and requirements from local health facilities
    i. **Time:** Discuss time schedule of mapping survey
    j. **Other:** Any further site specific requirements
12. Team to identify location for conducting the mapping with Community Leader
13. Team Leader to collect Community information on mobile
14. Team to inform local health facilities of mapping survey
    a. If no drugs are available the Team Leader should contact FMOH and inform them that treatment is required for individuals who are positive in the specific mapping site.
15. Put up sensitisation material in places where community will see to inform them of upcoming survey (e.g. schools, local health facilities and community meeting place)
16. Return to rest of field team and prepare for mapping survey the next day

Additional Notes:

(Write below any additional notes from the facilitator)

……
……
……
……
……
………

Day 2

Activities require **ALL MEMBERS OF THE FIELD TEAM**

8. Team members travel to site within district
9. Team meet with community leader and introduce team
10. Team members go to selected location and set up field mapping laboratory
    - c. **LaboratoryTechnician** to set up LF diagnosis station
    - d. **Nurses** to set up for data collection stations for LF and podoconiosis
11. **Community Leaders** assisted by local **FieldWorkers** round up community members to mapping laboratory
    - a. **Community Leaders** must lead the social mobilisation and community sensitisation showing their support of the mapping survey in the community and to highlight the benefits of participating in the survey
    - b. **Field Workers** should provide specific sensitisation on:
        - viii. LF – what it is and how it is transmitted
        - ix. Aim and purpose of mapping
        - x. Methodology and information collected
        - xi. Diagnostics used
        - xii. Benefits from participation (including availability of treatment from local health facility)
        - xiii. Ethical considerations and selection criteria
        - xiv. Time required to conduct mapping
    - c. Answer questions from community with support from **Team Leader, Translator**
    - d. Recruit and direct participants who voluntarily agree to take part to field laboratory
        - i. It may be time efficient to allocate time slots for individuals to come back to the mapping survey laboratory rather than waiting to be tested. **An example** of how the grouping system could work can be seen below:

> ***TIME SLOTS:*** *Participants are divided in 25 people groups. Eachgroup is given a time slot for when the participants should to return to the mapping survey laboratory.*
> *The first group should be individuals who are not requested to return but will be tested there and then. In the time slots below, 9am should be regarded as the group that are not requested to come back later.*
>
> - **Group 1 – 9am**
> - **Group 2 – 11am**
> - **Group 3 – 1pm**
> - **Group 4 – 3pm**

12. Conduct Mapping Survey (testing 100 eligible individuals)
13. End mapping
14. Inform community leaders that the mapping has been completed (if required)

Additional Notes:

(Write below any additional notes from the facilitator)

....
....
....
....
....
....
....
....
....
....
....
....
....
....
....
....
....
....

V. Mapping Survey

After the initial community sensitisation, the **Team Leader**, **Translator** and one **Field Worker** should return to the field laboratory to carry out their duties during the mapping survey.

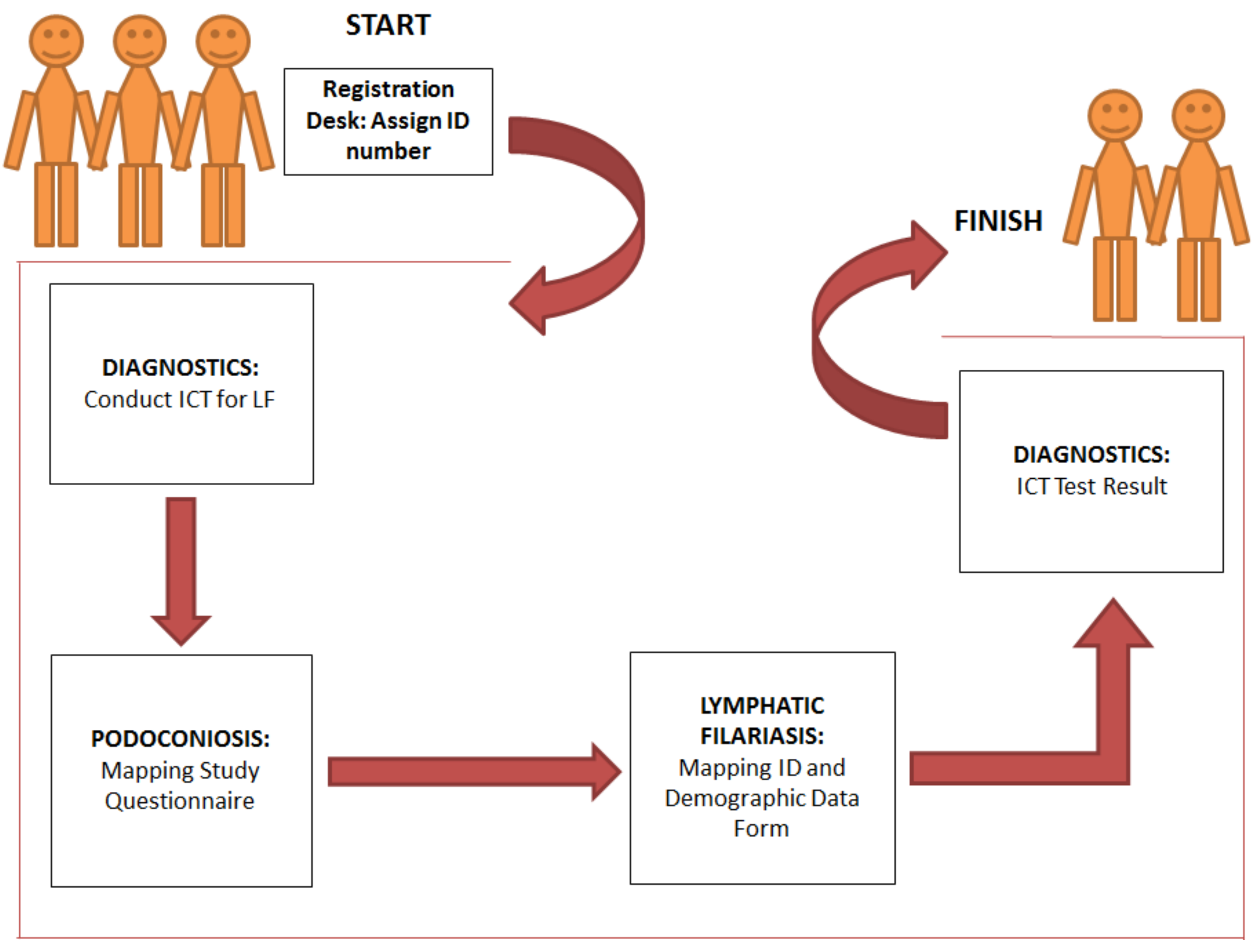

Figure 1: How the Mapping Survey should be set up

8. One**Field Worker** must be stationed at the registration desk as the first point of contact during the mapping survey.
   Their main role will be to ensure participants;
   - Meet the eligibility criteria
   - Are able to provide full signed consent (adult/ adult + assent)
   - Are allocated a unique id number
     - The last three numbers of the ID number should be on a piece of paper or the participant's hand for them to keep hold of and present at each data collection point (ICT card, LF mapping survey andpodoconiosis survey)
   - Further questions regarding the mapping are answered fully

   N.B. Participants shouldwait at registration until they are able to proceed in the mapping process. It is advisable not to register too many participants at once as this can increase the potential for errors in data collection. The second **Field Worker** should try to recruit individuals and encourage them to be patient whilst waiting to be seen.

9. After registration, the participants will visit the **Laboratory Technician**.
   It is essential that the following steps are followed:
   - Provide an overview of what the test is and how it will be conducted, showing all equipment that will be used to conduct the test

- Answer any questions regarding the test or the survey (participants will be fully informed about the survey prior to reaching the ICT card stage so general questions are not expected)
- Follow the standard operating procedure for ICT cards to collect the blood sample and conduct the test
- Write the ID number of the participant on the ICT card
- Provide a cotton wool to apply pressure on the puncture site (if required/requested by participant) after the blood has been collected
- Write on the ICT card the time when the test is started (when the blood reaches the pink part of the test). The test will be ready to be inspected after 10 minutes
- Inform the participant that they will be told the result by the **Team Leader**and it will take 10 minutes for the result to become clear

10. Participants will then move onto the**LF Nurse** to answer a series of pre-set questions. The Nurse must also conduct a physical assessment of the individual to detect physical signs and symptoms of LF.

11. Following the LF survey, the participant will move to the **podoconiosis Nurse** to answer a further series of pre-set questions. *(Further details on this section of the survey will be provided in the podoconiosis training).*

12. The ICT card result requires 10 minutes and it is anticipated that the test results will be available after **BOTH** questionnaires are complete. The **Laboratory Technician** conducting ICT cards should write the result of the test on the ICT card as soon as 10 minutes have passed. The **Team Leader** should collect the results and speak to all participants individually todeliver the results.

13. The **Team Leader** will discuss the results of the diagnostic test and provide further information if the test is positive.
    - Individuals who are **POSITIVE**should be given a standard letter which states their result. They should be directed to the local health facility where they can receive free treatment, upon presenting the letter.
    - Individuals who are **NEGATIVE** will require no further medical attention or treatment and are free to leave the mapping survey laboratory.

IN ABSENCE OF TREATMENT IN SITE: *If the local health centre does not have any treatment available for individuals who test positive, then the team leader must speak with the person in charge of the health centre to ensure individuals can be treated at a later date. The health centre will be requested to collect participants' names to ensure that treatment is allocated appropriately. If participants are not happy to provide their names, they should then be advised to bring the letter provided by the mapping survey team when they come to the health centre at a later date.*

The Team Leader should contact the LF Programme Manager at FMOH immediately and inform them that treatment is not available at the health centre and there are individuals who have tested positive by ICT card (Treatment is required if a minimum of 1 out of 100 people are found to be positive). The FMOH will be responsible for arranging transportation of drugs to the health facility and the individual in-charge of the health centre should be contacted by FMOH with further information regarding delivery.

The Team Leader must ensure the following information is provided to the FMOH LF Programme Manager:

- *Name of the Health Centre*
- *Province, Woreda and Kebele of the Health Centre*
- *Name of person in charge of the Health Centre*
- *Contact details of the person in charge of the Health Centre*

14. Once the participant knows their ICT card result, they are free to leave the mapping survey laboratory as their assessment has been complete.

Participants may have questions at any stage of the survey and all field team members must be prepared to answer them fully. If they do not know the answer, then the participant should be referred to the **Team Leader** who will be able to answer it.The **Team Leader** must also oversee all activities taking part in the mapping, ensuring the mapping protocol is followed and results are accurately captured.

The **Translator** must assist in all parts of the data collection where there is a language barrier. They may be required to translate disease specific information which must be translated accurately whilst remaining sensitive to cultural and language differences.

The **Field Workers** should work together; one bringing people from the community into the mapping survey laboratory, whilst the other registers them to conduct the testing. These roles are interchangeable and Field Workers may consider changing positions during the mapping.

Additional Notes:

(write below any additional notes from the facilitator)

VI. ………………………………………………………………

………………………………………………………………………
………………………………………………………………………
………………………………………………………………………
………………………………………………………………………
………………………………………………………………………
………………………………………………………………………
………………………………………………………………………
………………………………………………………………………
………………………………………………………………………
………………………………………………………………………
………………………………………………………………………
………………………………………………………………………
………………………………………………………………………
………………………………………………………………………
………………………………………………………………………
………………………………………………………………………
………………………………………………………………………
………………………………………………………………………

....
....
....
....
....
....
....
....
....
....
....
....
....
....
....
....
....
....
....
....
....
....
....
....
....
....

Data Collection using mobile Phones

SELECTING FORMS

To fill in each form the team leader/nurse will open the Task Force LINKS app on the android phone and click on the **'Fill Blank Form'** option and then the correct form; the community form, LF form, Podoconiosis form or the ICT result form.

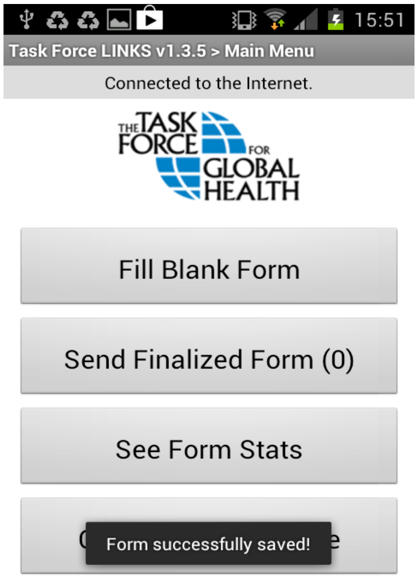

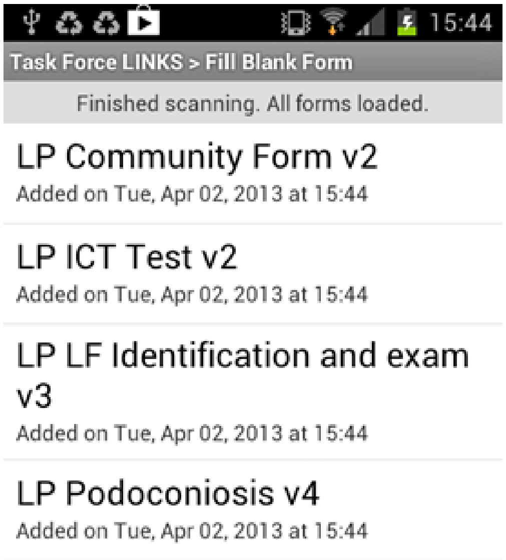

LP PODOCONIOSIS V4

The 'LP podoconiosis V4" is used to collect all the relevant information pertaining to the mapping of podoconiosis throughout the county. This form will be filled in by one of the nurse on the field team and must be done for each participant.

To fill in this form the nurse will open the Task Force LINKS app on the android phone and click on the **'Fill Blank Form'** option and then the **'**LP podoconiosis V4**'**. This will open the LP podoconiosis V4**'** form

The following fields will appear in order as the record collector enters the information and used the navigational arrows:

13. **Record Taker's Name:**
    - The record taker needs to type in their Name into the provided field
      ie: **John Smith** would type in **John Smith** into the provided field
14. **Woreda Code (001-692)**
    - Enter the 3 digit code used to identify the District/Woreda; ensure that you enter the 0 or 00 prior to the district number if it is needed; districts 1-9 require 00 before the number while districts 10-99 require 0 before the district number.
      ie: the district code for first district is **001**
15. **Community Code (0001-1384)**

- Enter the 4 digit code used to identify the Community; ensure that you enter the 0 or 00 prior to the community number if it is needed; communities 1-9 require 00 before the number while communities 10-99 require 0 before the district number. ie: the community code for first district is **0001**

16. **Individual ID Number (001-100)**
    - Record the individual's personal identification number; this should be found written on their hand.
17. **Age (15-100)**
    - Record the age of the individual in years
18. **Gender**
    - Select the correct gender for the participant; Male or Female
19. **Religion**
    - Select the correct religion for the participant: Muslim, Christian, Animist or Other
20. **Ethnic Group**
    - Select the correct ethnic group that the individual belongs to from the provided list
      - Oromo, Amhara, Somali, Tigre, Afar or Other
    - **If Other selected:** type in response
21. **Current occupation (Whatever the person does to earn money)?**
    - Select the correct occupation from the provided list
      - Employed, business man/woman, farmer, housewife etc
    - **If Other selected:** type in response
22. **How long have you lived at your current location?**
    - Record the amount of time the individual has lived in location (in years)
23. **Years of School Completed**
    - Record the number of years that the participant went to school
24. **What is the monthly income (on average) of your household including your own?**
    - Write the monthly income of the household in Birr.
25. **What type of floor does your house have?**
    - Select the correct response
      - Mud/earth, Wood, Cement , Other
    - **If Other selected:** type in response
26. **Current marital status?**
    - Select the correct response
      - Single, married , divorced, widowed
27. **Have you ever worn shoes?**
    - Select the correct answer to the question: yes or no
28. **How old were you when you first got shoes?**
    - Write age in years
29. **Is the person wearing shoes at the time of the interview?**
    - Select the correct answer to the question: yes or no
30. **Describe the shoes the person is wearing.**
    - Select the correct answer to the question: hard plastic, open sandal , Leather, Shera , other
      - **If Other selected:** type in response
31. When do you wear shoes?
    - Select the correct answer to the question: yes or no
    - At home**:**
      - Select the correct answer to the question: yes or no
    - During rainy
      - season Select the correct answer to the question: yes or no
    - On market days
      - Select the correct answer to the question: yes or no
    - On the field

    - Select the correct answer to the question: yes or no
- On Sundays
    - Select the correct answer to the question: yes or no
- When walking far
    - Select the correct answer to the question: yes or no

32. **How long (in min) does it take you to go to the nearest water source?**
    - Write distance in minutes
33. **When do you wash your feet?**
    - Select the correct answer to the question: whenever they are dirty, before sleeping, before prayer , Other
    - Select the correct answer to the question: yes or no
        - **If Other selected:** type in response
34. **How often do you wash your feet very carefully so that they are very clean?**
    - Select the correct answer to the question: more often than once a day , daily, less often than daily, but more often than weekly, weekly or less often.
35. **Do you have leg swelling?**
    - Select the correct answer to the question: yes or no
36. **Do you have any family member (living or dead) with history of leg swelling?**
    - Select the correct answer to the question: yes or no
37. **How many people in your family (living or dead) have leg swelling?**
    - Write in number
38. **How old were you when you first noticed this swollen leg?**
    - Write in number
39. **Where did the swelling start from?**
    - Select the correct answer to the question: from high up, from the foot or lower leg
40. **Do you have history of rheumatic heart disease?**
    - Select the correct answer to the question: yes or no
41. **Do you have swelling in the groin area?**
    - Select the correct answer to the question: yes or no
42. **Are you diagnosed as a leprosy patient?**
    - Select the correct answer to the question: yes or no
43. **Is there preservation of sensation in the toes? (Physical examination)**
    - Select the correct answer to the question: yes or no
44. **Podoconiosis diagnosis established**
45. **Podoconiosis disease stage**
    - Select the correct answer to the question:Stage 1, Stage 2, Stage 3 , Stage 4**,** Stage 5
46. **Antifilarial antibody test blood sample collected?**
    - Select the correct answer to the question: yes or no

Once all the information has been filled out a summary page will appear were you can double check all the information. If all the information is correct click the **'Save and Send Form'** button. This will finalize the form and return you to the home screen.

VII. Standard operating procedure for collecting blood sample

In areas where at least a case of ICT positive is found individuals with leg swelling will give 5 ml of blood for antibody test.

Universal precautions – handle all specimens as if they are capable of transmitting infectious agents.

Explain the procedure to the person and obtain informed consent.

Materials needed for blood sampling

- Disinfectant for skin (eg alcohol wipe)
- Sterile syringe and needle.
- Cotton wool or gauze
- vacutainers
- Nunc Tube
- Glove
- Micropipette and tip

Paperwork

1. Label the vacutainers with the individuals ID, Village ID and District ID. Use a ballpoint pen or other permanent marker directly on the nunk tube.

Sample collection

- Put on gloves, select the site of blood collection clean the selected area of skin with a skin disinfectant swab and allow to dry for 30 seconds.
- Using syringe and needle collect 5ml of blood.
- Filled the blood collection vacutainers should sit upright after the blood is filled at room temperature for a minimum of 30 to a maximum of 60 minutes to allow the clot to form.
- Use pipette to transfer the serum (Recommendation: do not pour!). Pipette serum into the labeled Nunck tube, filling the vials in sequential order. Close the caps on the vials tightly. This process should be completed within 1 hour of centrifugation.
- Store the serum in deep freezer.

VIII. Standard Operating procedure for ICT Cards

The ICT card test is a widely used sensitive tool for the detection of *W.bancrofti*antigen.

These tests are simple to use but require training to reduce the variability between observers and any misreading of the cards resulting in false positive results.

For the purpose of this mapping survey, only the Laboratory Technician in the field team will be conducting the LF diagnostic tests.

Guidelines
Storage and Transportation

3. At optimal storage conditions (4^0C), cards have an approximate shelf life of 9 months. However when stored at 30^0C the shelf life decreases to 3– 6 months.
4. When transporting cards to the study locations, it is advisable not to expose them to extreme heat for prolonged periods of time as this will rapidly decrease the shelf life.

Sample Collection

8. Put on a fresh pair of gloves.
9. Clean the site of the finger prick on the participant using a disinfectant wipe.
10. Using a lancet to prick the participant's finger, draw a small volume of blood.
11. From this prick, collect 100µL of blood using a capillary tube (supplied with ICT card).

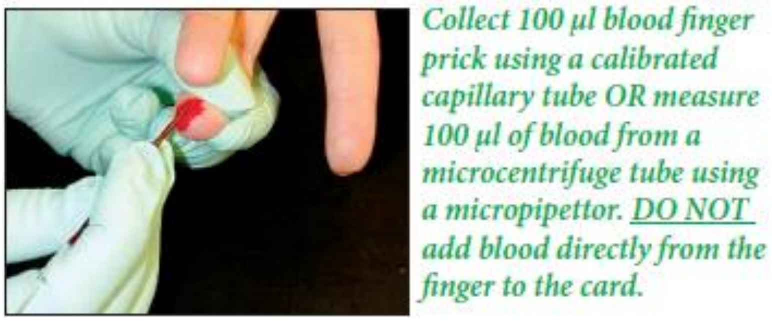

12. Add the collected blood sample to the white portion of the sample pad.
    - DO NOT add blood directly to the pink portion.
    - DO NOT close the card before the sample migrates to the pink portion; takes roughly 30 seconds.
    - Record the time when the blood reaches the pink portion on the card.
        - The test takes 10 minutes, starting from when the blood reaches the pink portion.

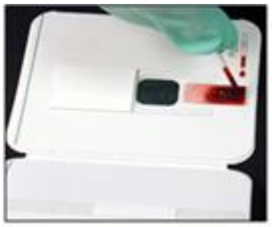

Add blood sample slowly to the white portion of the sample pad

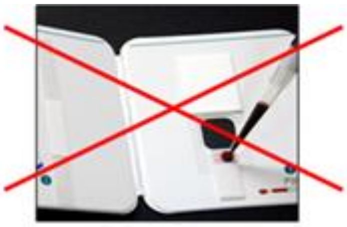

DO NOT add blood directly to the pink portion of the sample pad

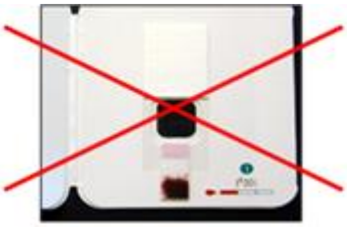

DO NOT close the card before the sample migrates to the pink portion of the sample pad (takes approximately 30 seconds after adding blood)

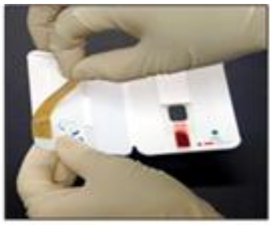

Remove adhesive liner and close card. Start timing.

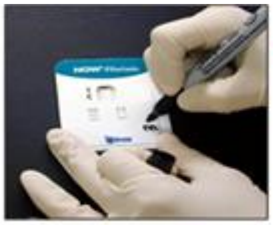

NOTE: It is helpful to record the starting time on the front of the card

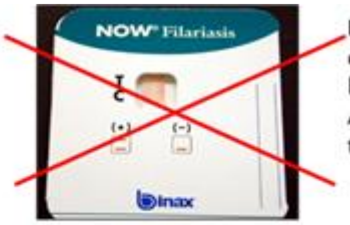

DO NOT read cards if the plasma has not flowed ALL the way down the strip.

NOTE: If plasma fails to migrate completely past the bottom of the window, a false positive result can occur

13. Read the results after 10 minutes and record the result by marking the card as positive or negative
    - DO NOT read the results at any other time as it can increase the chance of false positives
    - Ensure cards are read in a well-lit location, faint lines can be difficult to read if lighting is poor.
14. Safely dispose of the ICT card, capillary tube with any remaining blood found in the capillary tube
    - ICT card: ICT waste bag
    - Capillary Tube with any remaining blood: Sharps bin

Examples:
Below are example results from ICT cards showing the various results that can be seen on the card.

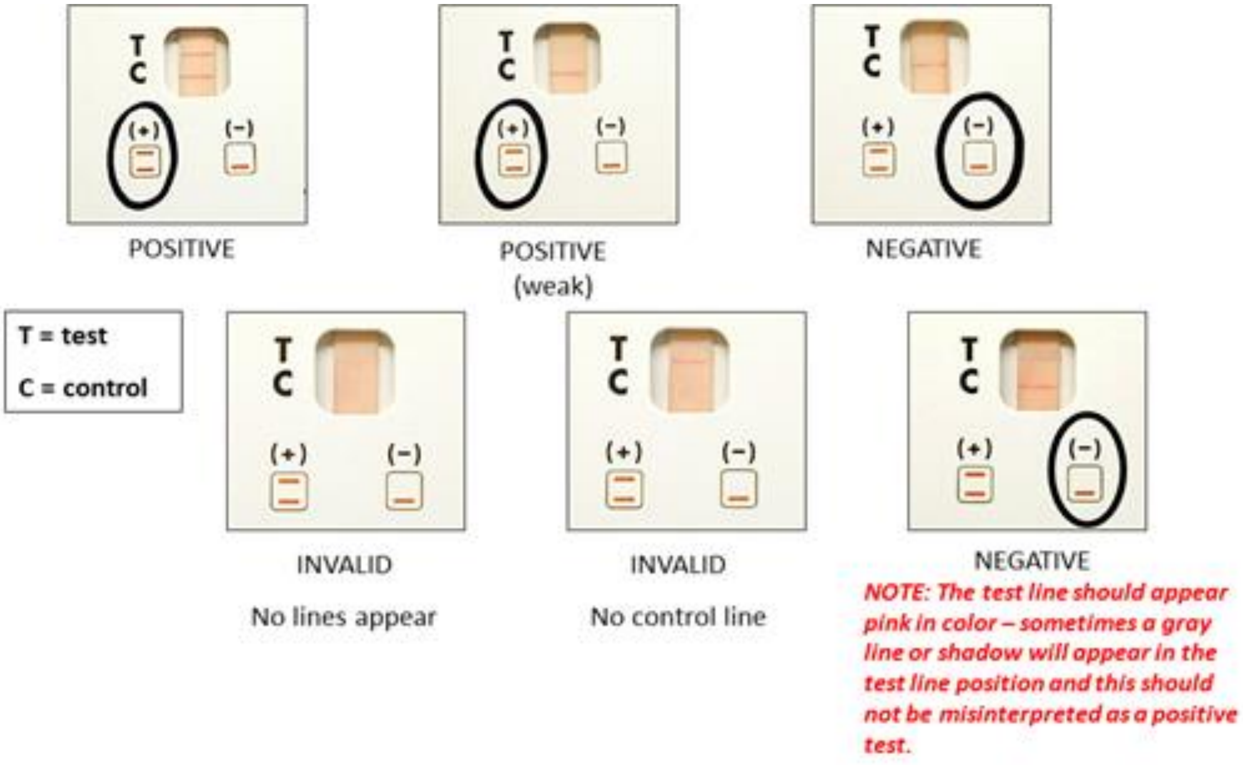

Reference:
World Health Organization, **2011**; *Global Programme to Eliminate Lymphatic Filariasis: Monitoring and Epidemiological Assessment of Mass Drug Administration*

IX. Checklist for Conducting survey

For each participant, it is essential to ensure the following:

ID Number – General

7. Allocate the ID number to each participant at the beginning
8. Ensure no name and/or address is collected at any point and matched with an ID number
9. The ID number should be written on ICT card prior to conducting test
10. ID number on ICT card must correspond to ID number on forms for podoconiosis and LF
11. Participant's ICT result must be entered onto participant's corresponding forms for podoconiosis and LF
12. ID numbers on podoconiosis form and LF form for one participant must identical

Laboratory Technician – Health & Safety

15. Ensure the work bench is cleaned with bleach periodically to keep the work area clean
16. Alcohol swabs after single use must be discarded in the general waste bag
17. Lancets, once used, must be discarded in the sharps bin
18. ICT cards, once results have been recorded, must be discarded in a waste bag specific for ICT cards

19. Capillary tubes, used to conduct ICT cards, must be discarded in sharps bins once used, irrespective of whether there is still blood in the tubes or not.
20. Broken capillary tubes must not be used and discarded in the sharps bins
21. Gloves must be worn at all times when conducting diagnostic tests
22. Ripped/torn gloves must be replaced immediately
23. Gloves should be replaced regularly throughout the testing
24. Use hand sanitizers regularly and use soap to wash hands before and after conducting ICT cards
25. Waste bins for biological samples should not be mixed with any other waste
26. Cotton wool balls should be provided to participants when requested, or when the wound is still bleeding
27. Biological samples in the waste bags must be incinerated before leaving the site
28. Sharps bins should be used until they are full. Once full they should be closed and stored until returning to central level for safe disposal

Laboratory Technician – Laboratory Procedures

5. Follow standard operating procedures for conducting ICT cards
6. Write the time when the blood reaches the pink portion of the test onto the card
7. Read ICT card after 10 minutes and write result on the test immediately
8. Results should only be shown/discussed to the Team Leader and not discussed with any participants in the survey

Nurses –Laboratory Procedures, Health & Safety

1. Answer all questions in the questionnaire
2. Ensure ID number entered accurately into mobile devices
3. Use hand sanitizers regularly
4. Wash hands with soap before and after examining patients for physical signs or symptoms of LF
5. Record ICT card result before storing data and moving onto the next participant
